# Signaling through the nicotinic acetylcholine receptor in the liver protects against the development of metabolic dysfunction-associated steatohepatitis

**DOI:** 10.1371/journal.pbio.3002728

**Published:** 2024-07-19

**Authors:** Heejin Jun, Shanshan Liu, Alexander J. Knights, Kezhou Zhu, Yingxu Ma, Jianke Gong, Ashley E. Lenhart, Xiaoling Peng, Yunying Huang, Jared P. Ginder, Christopher H. Downie, Erika Thalia Ramos, Klas Kullander, Robert T. Kennedy, X. Z. Shawn Xu, Jun Wu

**Affiliations:** 1 Life Sciences Institute, University of Michigan, Ann Arbor, Michigan, United States of America; 2 Department of Nutritional Sciences, College of Human Sciences, Texas Tech University, Lubbock, Texas, United States of America; 3 Department of Cardiology, The Second Xiangya Hospital, Central South University, Changsha, China; 4 International Research Center for Sensory Biology and Technology of MOST, Key Laboratory of Molecular Biophysics of MOE, and College of Life Sciences and Technology, and Huazhong University of Science and Technology, Wuhan, China; 5 Department of Chemistry, University of Michigan, Ann Arbor, Michigan, United States of America; 6 Department of Immunology, Genetics and Pathology, Uppsala University, Uppsala, Sweden; 7 Department of Pharmacology, University of Michigan, Ann Arbor, Michigan, United States of America; 8 Department of Molecular & Integrative Physiology, University of Michigan Medical School, Ann Arbor, Michigan, United States of America; Columbia University, UNITED STATES OF AMERICA

## Abstract

Metabolic dysfunction-associated steatohepatitis (MASH) is the progressive form of liver steatosis, the most common liver disease, and substantially increases the mortality rate. However, limited therapies are currently available to prevent MASH development. Identifying potential pharmacological treatments for the condition has been hampered by its heterogeneous and complex nature. Here, we identified a hepatic nonneuronal cholinergic signaling pathway required for metabolic adaptation to caloric overload. We found that cholinergic receptor nicotinic alpha 2 subunit (CHRNA2) is highly expressed in hepatocytes of mice and humans. Further, CHRNA2 is activated by a subpopulation of local acetylcholine-producing macrophages during MASH development. The activation of CHRNA2 coordinates defensive programs against a broad spectrum of MASH-related pathogenesis, including steatosis, inflammation, and fibrosis. Hepatocyte-specific loss of CHRNA2 signaling accelerates the disease onset in different MASH mouse models. Activation of this pathway via pharmacological inhibition of acetylcholine degradation protects against MASH development. Our study uncovers a hepatic nicotinic cholinergic receptor pathway that constitutes a cell-autonomous self-defense route against prolonged metabolic stress and holds therapeutic potential for combatting human MASH.

## Abstract 2

The complexity of metabolic dysfunction-associated steatohepatitis (MASH) has impaired the development of pharmacological treatments for the condition. This study shows that nicotinic acetylcholine receptor signaling serves as an intrinsic protective mechanism against extended metabolic stress in the liver.

## Introduction

The increasing global prevalence of metabolic dysfunction-associated steatohepatitis (MASH) and the subsequent high burden on healthcare systems have generated impetus for better understanding and therapeutically targeting this condition [1,2]. Since MASH is strongly associated with obesity and type 2 diabetes, the effects of insulin sensitizers, such as glucagon-like peptide-1 receptor agonists and pioglitazone, have been explored in MASH [3,4]. The first US Food and Drug Administration (FDA)-approved pharmacological therapy for MASH, resmetirom, has recently become available after long, intensive research efforts. This bolsters hopes for most drug targets still in Phase 2 or 3 trials and for further advances in MASH research and therapies [5,6]. However, despite recent discoveries highlighting the complexity and heterogeneity of MASH pathogenesis, which includes multiple parallel pathological insults, inter-organ crosstalk, and dynamic cell-to-cell interactions [7–11], key molecular factors driving the development of this disease, and their mechanisms, remain to be elucidated.

The nicotinic acetylcholine receptors (nAChRs) have received considerable attention as drug targets for many human diseases due to their broad distribution across neuronal and nonneuronal systems, their subunit-based unique structures, and their wide array of physiological and pathological functions [12]. Recent studies have revealed metabolic functions of nAChRs in the liver; however, these findings were primarily limited to immune cells, and thus distinct cell type-specific functions of nAChRs are mostly unknown in the liver under physiological and pathophysiological conditions [13–15]. Moreover, the source of acetylcholine that stimulates hepatic nAChR signaling is unclear due to debates over the presence of parasympathetic nerves and/or their significance in the liver [16–21].

Here, we identify high expression of cholinergic receptor nicotinic alpha 2 subunit (CHRNA2), an nAChR subunit, in mouse and human hepatocytes. We show that CHRNA2 is activated via paracrine signaling from local macrophage-derived acetylcholine during the onset of MASH. Such activation acts as an adaptive response to caloric overload to mitigate MASH-related pathogenesis, while hepatocyte-specific loss-of-function of CHRNA2 accelerates the disease development. Finally, pharmacological activation of this pathway in murine MASH models inhibits the disease progression. Together, our data identify a novel pathway involved in the response to hypercaloric hepatic stress that can be effectively targeted to attenuate MASH development.

## Results

### CHRNA2 signaling in hepatocytes is activated during metabolic adaptation to caloric overload

**Fig 1:**
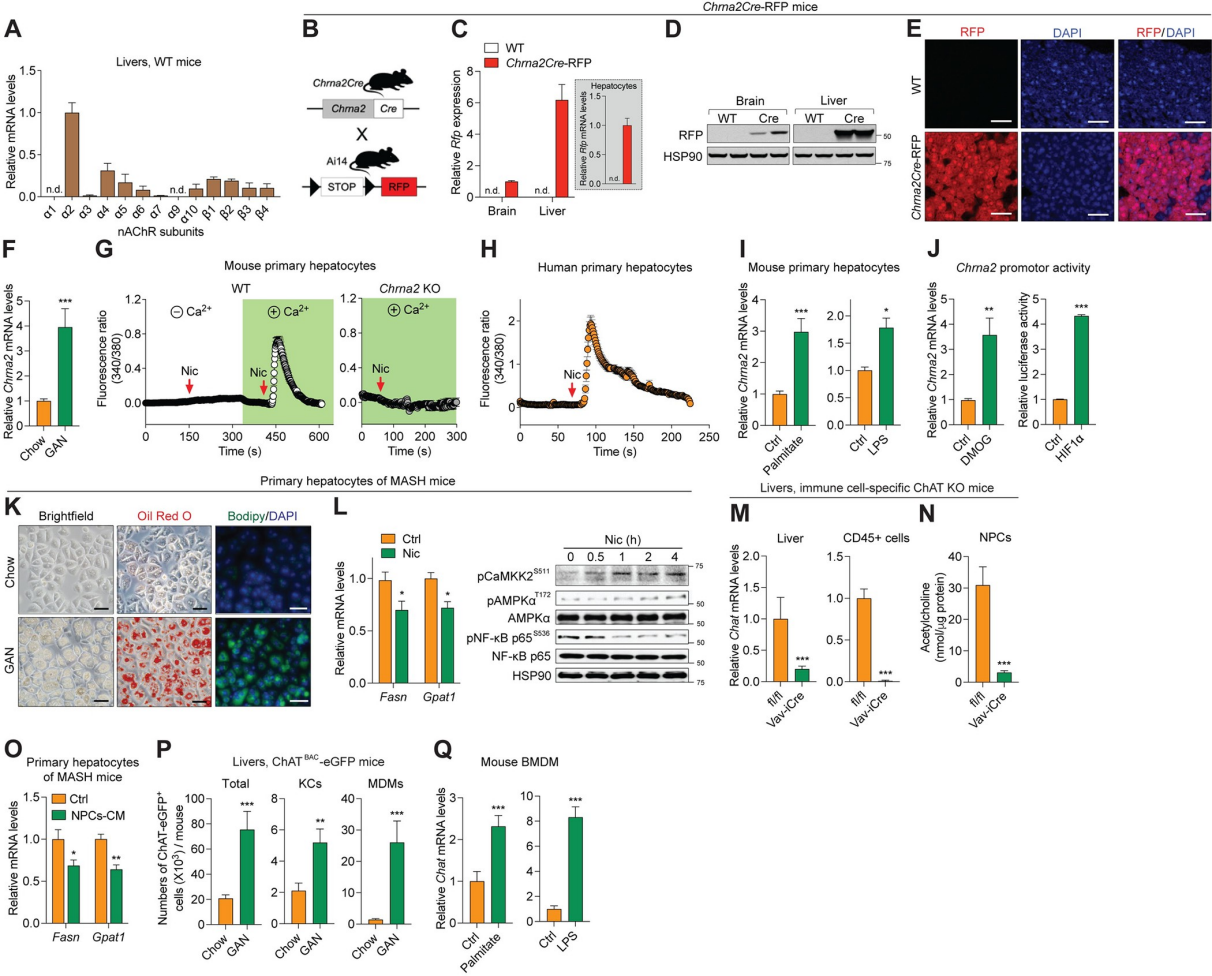
Hepatocyte CHRNA2 signaling is activated during metabolic adaptation to caloric overload. (A) qPCR analyses of genes encoding nAChR subunits in WT mouse livers (*n* = 9). (B) Schematic diagram describing the generation of *Chrna2Cre*-RFP mice by crossing *Chrna2Cre* and Ai14 mice. (C) qPCR analyses of *Rfp* expression in brains, livers, and primary hepatocytes of WT and *Chrna2Cre*-RFP mice (WT, *n* = 6; Cre, *n* = 6). (D) Immunoblot analyses for *Chrna2Cre*-mediated RFP and HSP90 (loading control) expression in brain and liver tissues of WT and *Chrna2Cre*-RFP mice. (E) RFP signals of liver sections of WT and *Chrna2Cre*-RFP mice. DAPI was used to stain nuclei. Scale bar, 50 μm. (F) qPCR analyses of *Chrna2* expression in liver tissues of WT mice fed with a chow diet or chronic Gubra-Amylin NASH (GAN) diet (Chow, *n* = 9; GAN, *n* = 5). (G) Intracellular calcium uptake mediated by the CHRNA2 agonist nicotine (Nic, 500 μm) in the presence or absence of calcium in primary hepatocytes from control WT and *Chrna2* KO mice (WT, *n* = 17; KO, *n* = 14). (H) Intracellular calcium uptake mediated by 500 μm Nic in human primary hepatocytes (*n* = 31). (I) qPCR analyses of *Chrna2* in primary WT mouse hepatocytes treated with vehicle (Ctrl), palmitate (0.25 mM for 16 h; left, *n* = 6 per group), or LPS (10 μg/ml for 4 h; right, *n* = 3 per group). (J) Left, qPCR analyses of *Chrna2* in primary WT mouse hepatocytes treated with vehicle (Ctrl) or 1 mM dimethyloxalylglycine (DMOG) for 20 h (*n* = 4 per group). Right, *Chrna2* transcriptional activity using a mouse *Chrna2*-promoter luciferase reporter construct with control vector or an *HIF1α*-expressing vector (*n* = 5 per group). (K) Morphology of primary hepatocytes isolated from chow or GAN diet-fed WT mice. Lipids droplets were stained with Oil Red O or Bodipy. DAPI was used to stain nuclei. Scale bar, 50 μm. Representative images are shown. (L) Left, qPCR analyses of hepatic lipogenic genes in vehicle (Ctrl) or Nic (2 mM for 6 h)-treated primary hepatocytes isolated from GAN diet-fed WT mice (Ctrl, *n* = 6; Nic, *n* = 5). Right, immunoblot analyses for CHRNA2-mediated signaling proteins and HSP90 (loading control) against MASH development in primary hepatocytes of GAN diet-fed WT mice after 2 mM Nic treatment for the indicated amount of time. (M) qPCR analyses of *Chat* in liver tissues (*Chat*^fl/fl^, *n* = 10; *Chat*^fl/fl^;*Vav-iCre*, *n* = 17) and sorted hepatic CD45^+^ cells (*Chat*^fl/fl^, *n* = 5; *Chat*^fl/fl^;*Vav-iCre*, *n* = 3). (N) Quantification of acetylcholine secreted from liver NPCs (*n* = 6 per group). (O) qPCR analyses of hepatic lipogenic genes in primary hepatocytes isolated from GAN diet-fed WT mice following treatment with control (Ctrl) or NPCs CM for 4 h (Ctrl, *n* = 5; NPCs-CM, *n* = 6). (P) Flow cytometric analyses for the abundance of total cells, Kupffer cells (KCs) and MDMs that express ChAT in liver NPCs from ChAT^BAC^-eGFP mice fed with chow diet or GAN diet (Chow, *n* = 10; GAN, *n* = 6). (Q) qPCR analyses of *Chat* in primary BMDMs stimulated with vehicle, 0.5 mM palmitate for 7 h (left; *n* = 6 per group) or 50 ng/ml LPS for 4 h (right; *n* = 6 per group). The data underlying the graphs in this figure can be found in S1 Data and S1 Raw Images. Mean ± SEM. n.d., not detected. *p < 0.05, **p < 0.01, ***p < 0.005 by an unpaired two-sample Student’s *t* test or Mann–Whitney U test. BMDM, bone marrow-derived macrophage; CM, conditioned medium; KO, knockout; LPS, lipopolysaccharides; MASH, metabolic dysfunction-associated steatohepatitis; MDM, monocyte-derived macrophage; nAChR, nicotinic acetylcholine receptor; NPC, non-parenchymal cell; WT, wild-type.

To identify the role of nAChRs and their therapeutic potential in liver disease, we first measured the expression levels of nAChRs in mouse liver under homeostatic conditions. In chow diet-fed wild-type (WT) mice, among all nAChR subunits, *Chrna2* was the most highly expressed in the liver (Figs 1A, S1A and S1B). A transgenic animal model, *Chrna2Cre*-RFP (red fluorescent protein) reporter mouse, visualized and confirmed robust expression of hepatocyte *Chrna2* in vivo (Fig 1B–1E). Interestingly, compared to chow-fed controls, hepatic *Chrna2* dynamically responded to metabolic stress and was up-regulated approximately 4-fold in livers from mice challenged with a Gubra-Amylin non-alcoholic steatohepatitis diet (GAN diet), which has been shown to cause hepatic steatosis, inflammation, and fibrosis, similar to morphological characteristics in human MASH [22] (Figs 1F and S1C). Consistent with this observation, publicly available human transcriptomic data revealed that *CHRNA2* was readily detectable in healthy livers and that individuals with obesity or type 2 diabetes, which commonly appear with MASH as its risk factors and comorbidities, tended to have higher hepatic *CHRNA2* expression than healthy control subjects (S1D–S1F Fig).

Fractionation of mouse liver tissue revealed that *Chrna2* was enriched in hepatocytes compared to non-parenchymal cells (NPCs) (S1G and S1H Fig). As a subunit of the nAChRs, CHRNA2 forms a functional pentameric ion channel in response to agonist signaling [23]. In WT mouse hepatocytes stimulated with agonists for nAChRs, nicotine or acetylcholine, intracellular calcium (Ca^2+^) levels were induced, but not in *Chrna2* knockout (KO) cells, indicating that functional CHRNA2 protein is present and no other nAChR subunit is compensating for the absence of CHRNA2 in hepatocytes (Figs 1G, S1I and S1J). No response to either ligand was observed in the absence of extracellular Ca^2+^ (Figs 1G and S1J), consistent with an ionotropic rather than a metabotropic mechanism. Importantly, functional CHRNA2 signaling was conserved in human hepatocytes, including primary cells and the HepG2 cell line (Figs 1H, S1K and S1L).

We tested whether CHRNA2 has a distinct metabolic function in hepatocytes responding to metabolic stresses stemming from caloric overload. Pathological mechanisms of high calorie-induced liver diseases, including steatotic liver (SL) and MASH, encompass hepatic accumulation of excess free fatty acids (FFAs) from liver lipogenesis or circulation released from adipose tissues and hepatic exposure to gut-derived endotoxin, primarily lipopolysaccharides (LPS) [1,9,24]. In the presence of palmitate or LPS, we observed a significant induction of *Chrna2* expression in primary WT mouse hepatocytes and in HepG2 cells (Figs 1I and S1M). In addition, cellular oxygen sensing and hypoxia-inducible factor (HIF) signaling have been implicated in the pathogenesis of diet-induced metabolic liver diseases [25,26]. *Chrna2* expression was significantly increased in primary hepatocytes treated with dimethyloxalylglycine (DMOG) that increases the expression levels of HIF1 subunit alpha (HIF1α) via inhibition of HIF-prolylhydroxylase (Fig 1J). The direct activating effect of HIF1α on *Chrna2* promotor activity was further confirmed via a luciferase assay (Fig 1J). Further downstream analyses suggested that CHRNA2 may serve as a self-defense mechanism during metabolic adaptation to hypercaloric stress. To test the hepatocyte-autonomous role of CHRNA2 signaling, we adopted a previously reported in vitro system that employs primary hepatocyte culture from chronic GAN diet-fed mice, in which alive but injured hepatocytes exhibit lipid accumulation as seen in vivo (Fig 1K) [27,28]. In primary hepatocytes, treatment with the CHRNA2 agonist nicotine suppressed mRNA expression of de novo lipogenesis markers involved in the development of diet-induced liver diseases (Fig 1L). At the cell-autonomous level, the CHRNA2-dependent protective effects were mechanistically initiated by Ca^2+^-triggered phosphorylation of Ca^2+^/calmodulin-dependent protein kinase kinase 2 (CaMKK2) and mediated, at least in part, through phosphorylation of adenosine monophosphate (AMP)-activated protein kinase (AMPK), an energy sensor known to attenuate diet-induced SL [29–32], and through inactivation of its downstream target nuclear factor kappa B (NF-κB), a regulator of hepatic inflammation and injury (Figs 1L and S1N) [33].

We next identified the source of the agonist for hepatic CHRNA2 activation. The presence of parasympathetic nerves in the liver has been debated [16–21], and acetylcholine-synthesizing nonneuronal cells have been reported to participate in cholinergic regulation in peripheral tissues [34–36]. Therefore, we investigated whether a specific nonneuronal cell type is a part of the hepatic cholinergic system. Choline acetyltransferase (ChAT) is the enzyme responsible for acetylcholine biosynthesis, and thus its activity is regarded as a specific biomarker for the functional state of acetylcholine-producing cells [37–40]. We found that *Chat* expression was enriched in NPCs relative to hepatocytes in WT mouse livers (S2A Fig). Among total hepatic NPCs isolated from ChAT^BAC^-eGFP reporter mice, about 1.5% were GFP^+^, with CD45^+^ B cells (19.7% B220^+^, 34% B220^-^), T cells (17.7% CD4^+^, 1.1% CD8^+^), and macrophages (9.5% Kupffer cells, KCs, 6.2% monocyte-derived macrophages excluding neutrophils, MDMs) accounting for the majority of GFP^+^ cells (S2B and S2C Fig and S1 Table).

*Chat* expression was greatly lower in livers from *Chat*^fl/fl^;*Vav-iCre* mice upon hematopoietic lineage-specific *Chat* deletion, compared to floxed controls, demonstrating that a significant portion of hepatic *Chat* expression was derived from immune cells (Fig 1M). Ablation of *Chat* expression in immune cells was further confirmed in sorted CD45^+^ cells (Fig 1M). We next used mass spectrometry analysis to confirm that at a functional level, secretion of acetylcholine from hepatic NPCs was strikingly lower in *Chat*-deficient immune cells, compared to NPCs from floxed control mice (Fig 1N). Hepatic NPC-conditioned medium (CM) reduced transcriptional levels of lipogenic markers in injured primary hepatocytes from chronic GAN diet-fed mice, suggesting an interaction between NPCs and hepatocytes through acetylcholine (Fig 1O). In line with evidence from prior studies, these data emphasize the contribution of immune cells in regulating liver function at the physiological level [41,42].

We next examined whether hepatic ChAT-expressing immune cells respond to hypercaloric stress, leading to mobilization of the hepatic acetylcholine reservoir. After challenging ChAT^BAC^-eGFP mice with a GAN diet, the abundance of hepatic ChAT^+^ immune cells was greater than in chow-fed control mice, in part via dynamic changes in the macrophage pool, including resident KCs and recruited MDMs (Figs 1P, S2D and S2E). GAN diet feeding increased ChAT-eGFP expression, as analyzed by median fluorescence intensity (MFI), in hepatic ChAT^+^ KCs and MDMs (S2F Fig). We further confirmed the direct influence of metabolic stresses in the regulation of *Chat* expression in primary KCs and bone marrow-derived macrophages (BMDM) from WT mice. Treatment with palmitate or with LPS significantly induced *Chat* expression in primary mouse macrophages (Figs 1Q, S2G and S2H). Similar activation of the ChAT-CHRNA2 axis was observed in the liver and hepatocytes isolated from obese mice with high-fat diet (HFD) feeding (S3A–S3C Fig). These results collectively suggest that a functional acetylcholine-CHRNA2 signaling axis exists in the liver, and that it may serve as a defense mechanism in response to metabolic stresses that promote liver injury.

### Deficiency of hepatic CHRNA2 signaling accelerates the onset of diet-induced MASH

**Fig 2:**
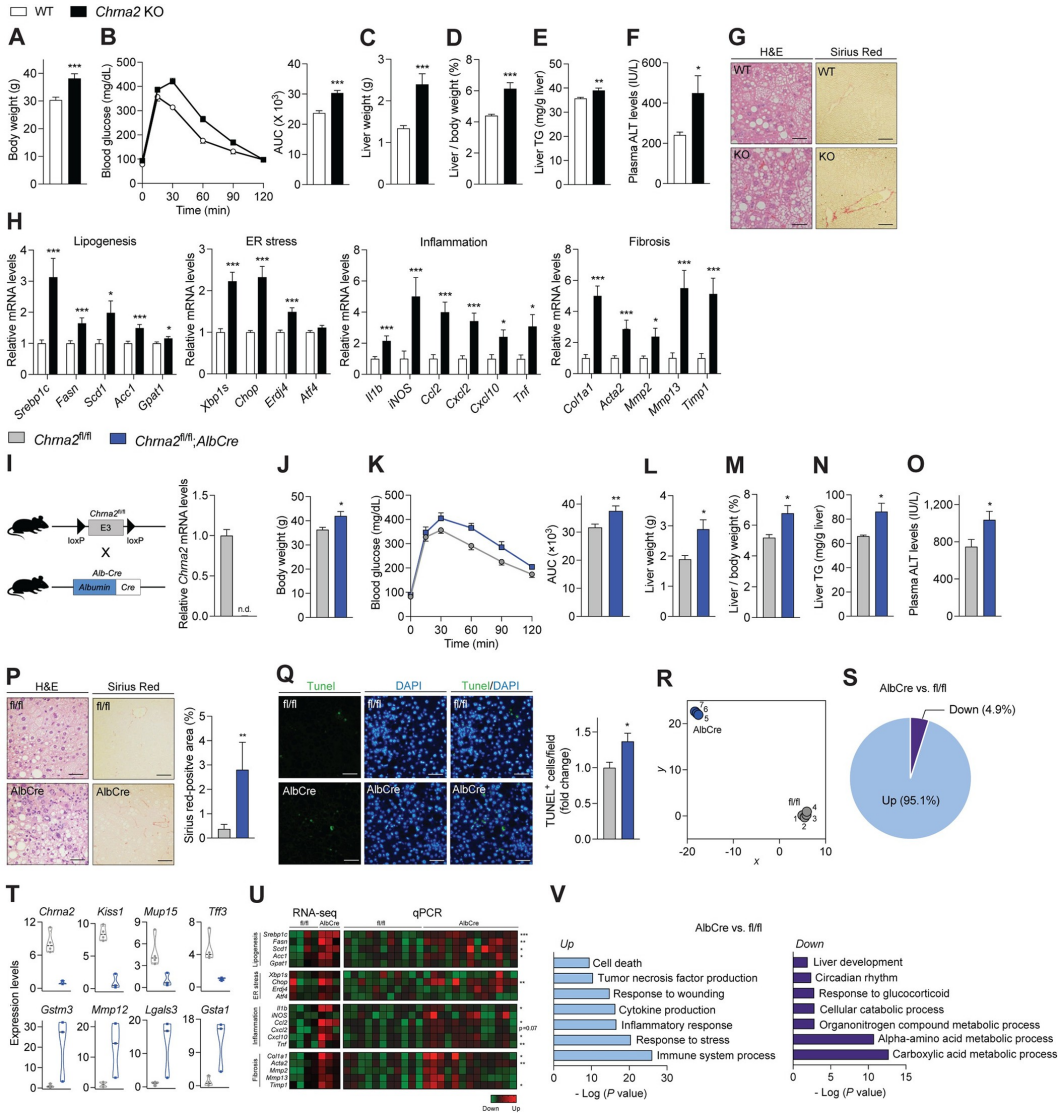
Deficiency of hepatic CHRNA2 signaling accelerates the onset of diet-induced MASH. (A–H) Control WT and whole-body *Chrna2* KO mice following 13-week Gubra-Amylin NASH (GAN) diet feeding. (A) Body weight (WT, *n* = 12; KO, *n* = 11). (B) GTT (*n* = 10 per group). AUC, area under the curve. (C) Liver weight (WT, *n* = 12; KO, *n* = 11). (D) Liver per body weight ratio (WT, *n* = 12; KO, *n* = 11). (E) Liver triglyceride (TG; *n* = 15 per group). (F) Plasma alanine aminotransferase (ALT; *n* = 10 per group). (G) HE and Sirius Red staining of liver sections. Scale bars, 50 μm. Representative images are shown. (H) qPCR analyses of MASH pathogenic genes in livers (WT, *n* = 12; KO, *n* = 11). (I–V) Control *Chrna2*^fl/fl^ and *Chrna2*^fl/fl^;*AlbCre* mice following prolonged GAN diet feeding. (I) Left, schematic diagram illustrating the generation of liver-specific *Chrna2* deleted mice by crossing *Chrna2*^fl/fl^ with *AlbCre* mice. Right, qPCR analyses of *Chrna2* mRNA expression in livers (*Chrna2*^fl/fl^, *n* = 10; *Chrna2*^fl/fl^;*AlbCre*, *n* = 7). (J) Body weight (*n* = 6 per group). (K) GTT (*Chrna2*^fl/fl^, *n* = 14; *Chrna2*^fl/fl^;*AlbCre*, *n* = 12). AUC, area under the curve. (L) Liver weight (*n* = 6 per group). (M) Liver per body weight ratio (*n* = 6 per group). (N) Liver TG (*n* = 12 per group). (O) Plasma ALT (*Chrna2*^fl/fl^, *n* = 15; *Chrna2*^fl/fl^;*AlbCre*, *n* = 12). (P) Left, HE and Sirius Red staining of liver sections. Scale bars, 50 μm. Representative images are shown. Right, quantification of Sirius Red-positive area (*Chrna2*^fl/fl^, *n* = 8; *Chrna2*^fl/fl^;*AlbCre*, *n* = 10). (Q) Left, TUNEL staining of liver sections. Scale bars, 50 μm. Representative images are shown. Right, the relative frequencies of TUNEL-positive cells per field (*Chrna2*^fl/fl^, *n* = 8; *Chrna2*^fl/fl^;*AlbCre*, *n* = 10). (R) Uniform Manifold Approximation and Projection analysis of gene expression profile from liver RNA sequencing (RNA-seq) (*Chrna2*^fl/fl^, *n* = 4; *Chrna2*^fl/fl^;*AlbCre*, *n* = 3). (S) The relative frequencies of differentially expressed genes from liver RNA-seq (*Chrna2*^fl/fl^, *n* = 4; *Chrna2*^fl/fl^;*AlbCre*, *n* = 3). (T) Significantly differentially expressed genes in *Chrna2*^fl/fl^;*AlbCre* mice relative to controls (*Chrna2*^fl/fl^, *n* = 4; *Chrna2*^fl/fl^;*AlbCre*, *n* = 3). (U) The mRNA expression of selected MASH pathogenic genes from RNA-seq (*Chrna2*^fl/fl^, *n* = 4; *Chrna2*^fl/fl^;*AlbCre*, *n* = 3) and validation using qPCR (*Chrna2*^fl/fl^, *n* = 11; *Chrna2*^fl/fl^;*AlbCre*, *n* = 13). (V) Biological pathway analysis of up- or down-regulated genes in *Chrna2*^fl/fl^;*AlbCre* mice relative to controls from (R). The data underlying the graphs in this figure can be found in S2 Data. Mean ± SEM. n.d., not detected. *p < 0.05, **p < 0.01, ***p < 0.005 by an unpaired two-sample Student’s *t* test or Mann–Whitney U test. CHRNA2, cholinergic receptor nicotinic alpha 2 subunit; GTT, glucose tolerance test; HE, hematoxylin-eosin; KO, knockout; MASH, metabolic dysfunction-associated steatohepatitis; WT, wild-type.

To test the physiological impact of activated hepatic acetylcholine-CHRNA2 signaling during metabolic adaptation upon calorie overload, we adopted an early disease stage of the diet-induced MASH model with reversible pathologies. Therefore, mice were exposed to a GAN diet for 18 weeks that developed mild MASH phenotypes with steatosis, hepatocellular ballooning, and modest fibrosis [43]. We examined the animals for the development of diet-induced MASH, including steatosis, hepatocyte injury, inflammation, and fibrosis, which are known to cause and characterize the disease phenotypes [1,11]. Under normal housing conditions and chow diet feeding, whole-body *Chrna2* KO mice showed no gross abnormalities in liver morphology and function (S4A–S4E Fig). However, after GAN diet feeding, *Chrna2* KO mice showed higher body weight and worse glucose tolerance without food intake difference than WT control mice (Figs 2A, 2B, S4F and S4G). The absence of CHRNA2 signaling resulted in significant hepatomegaly with elevated hepatic triglyceride (TG) levels and plasma alanine aminotransferase (ALT) levels compared to controls after GAN diet feeding (Fig 2C–2F). HE and Sirius Red staining suggested aggravated liver morphology with lipid accumulated or fibrotic areas increased in *Chrna2* KO mice relative to controls (Fig 2G). Consistently, qPCR analysis revealed that key genes that drive the onset and development of MASH, such as regulators of lipogenesis (*Srebp1*, sterol regulatory element-binding transcription factor 1; *Fasn*, fatty acid synthase; *Scd1*, stearoyl-Coenzyme A desaturase 1; *Acc1*, acetyl-Coenzyme A carboxylase alpha; *Gpat1*, glycerol-3-phosphate acyltransferase), endoplasmic reticulum (ER) stress (*Xbp1s*, X-box binding protein 1, spliced form; *Chop*, DNA-damage inducible transcript 3; *Erdj4*, DnaJ heat shock protein family member B9; *Atf4*, activating transcription factor 4), inflammation [*Il1b*, interleukin 1 beta; *iNOS*, nitric oxide synthase 2, inducible; *Ccl2*, chemokine (C-C motif) ligand 2; *Cxcl2*, chemokine (C-X-C motif) ligand 2; *Cxcl10*, chemokine (C-X-C motif) ligand 10; *Tnf*, tumor necrosis factor] and fibrogenesis (*Col1a1*, collagen, type I, alpha 1; *Acta2*, actin alpha 2, smooth muscle, aorta; *Mmp2*, matrix metallopeptidase 2; *Mmp13*, matrix metallopeptidase 13; *Timp1*, tissue inhibitor of metalloproteinase 1), were expressed at a higher level in *Chrna2* KO livers relative to WT when challenged with a GAN diet (Fig 2H). The *Chrna2* deficiency-associated liver damages were concomitant with increased SREBP1 maturation and phosphorylation of c-Jun NH2-terminal kinase (JNK) and NF-κB [33,44,45] (S4H Fig).

We then expanded our investigation to explore the potential contribution of non-hepatic CHRNA2 signaling to GAN diet-induced liver disease phenotype and related metabolic dysfunction. Our previous study discovered a functional CHRNA2 signaling responsible for beige fat thermogenesis in subcutaneous adipose tissue [37,46]. We found minimal transcriptional changes in the thermogenic response to MASH-inducing GAN diet in the inguinal white adipose tissue (iWAT) of *Chrna2* KO mice compared with controls (S4I Fig). Lipolysis-liberated fatty acids from adipose tissue can exacerbate hepatic steatosis through increased uptake in the liver [47]. Yet, little differences in gene expression levels of lipolysis markers were observed in the iWAT of KO mice compared to those of controls after GAN diet feeding (S4I Fig). Additionally, no changes in the adaptive thermogenic transcriptional regulation of brown adipose tissue (BAT) and skeletal muscle with *Chrna2* deficiency consistently supported the negligible contribution of thermogenic defects in MASH development upon a GAN diet challenge (S4J–S4L Fig).

We further probed the hepatocyte-specific effects of CHRNA2 signaling in the onset of MASH using a hepatocyte-restricted *Chrna2* knockout mouse model (*Chrna2*^fl/fl^*;AlbCre*) (Fig 2I). *Chrna2* deletion in the liver was confirmed by qPCR, with no deletion of *Chrna2* seen in other tissues (Figs 2I and S5A). On a chow diet, body weight, food intake, liver mass and morphology, and hepatic functional gene expression were comparable between *Chrna2*^fl/fl^*;AlbCre* mice and *Chrna2*^fl/fl^ mice (S5B–S5F Fig). Additionally, iWAT with functional CHRNA2 signaling for beige thermogenesis was normal in its mass and thermogenic gene expression profile in hepatocyte-specific *Chrna2*-deleted mice under the basal condition (S5G Fig). After GAN diet challenge, *Chrna2*^fl/fl^;*AlbCre* mice demonstrated exacerbated systemic metabolic abnormality with higher body weight gain and glucose intolerance compared to *Chrna2*^fl/fl^ mice without food intake difference between the genotypes (Figs 2J, 2K and S5H–S5J). Advanced diet-induced MASH-related phenotypes were seen in *Chrna2*^fl/fl^;*AlbCre* mice with enlarged liver, increased liver to body weight ratio, increased plasma ALT levels, hepatic steatosis, fibrosis, and cell death relative to controls (Fig 2L–2Q). As determined by thermogenic gene expression levels in iWAT, BAT, skeletal muscle, and core body temperature, no obvious differences in adaptive thermogenesis were found between the 2 genotypes after GAN diet feeding (S5K–S5N Fig). Therefore, these data using *Chrna2*^fl/fl^;*AlbCre* mice suggest both the hepatic and the systemic defects are most likely to be direct consequence of hepatocyte-specific *Chrna2* deletion in response to a GAN diet. Furthermore, moderately higher blood TG levels in *Chrna2*^fl/fl^;*AlbCre* mice relative to controls after GAN diet feeding suggested that activated hepatic lipogenesis may play a causative role in exacerbated body weight gain and disease phenotypes in *Chrna2* deficiency (S5O Fig).

The liver tissues were collected for RNA sequencing (RNA-seq) to further test the consequences of hepatocyte *Chrna2* deficiency in diet-induced MASH development at the transcriptome level. An unbiased molecular landscape, generated by Uniform Manifold Approximation and Projection analysis, revealed a clear distinction between global gene expression profiles of *Chrna2*^fl/fl^ and *Chrna2*^fl/fl^;*AlbCre* (Fig 2R). The majority of differentially expressed genes (DEGs) were transcriptionally up-regulated in mutant livers relative to controls (Fig 2S). Among the DEGs, many highly up-regulated genes in the mutant livers have been implicated in susceptibility to metabolic stress and disease development, such as glutathione S-transferase, mu 3 (*Gstm3*), matrix metallopeptidase 12 (*Mmp12*), lectin, galactose binding, soluble 3 (*Lgals3*) and glutathione S-transferase, alpha 1 (*Gsta1*) [48–51] (Fig 2T). In addition to *Chrna2*, kisspeptin (*Kiss1*), major urinary protein 15 (*Mup15*) and trefoil factor 3, intestinal (*Tff3*) were identified among genes with the most decreased expression levels in mutant livers compared to control livers, which have been shown to be related to metabolic protection [52–54] (Fig 2T). We further validated that expression levels of selected MASH pathogenic markers were higher in mutant livers than controls using both RNA-seq and qPCR analyses (Fig 2U). Pathway analysis identified MASH-related signaling pathways, including cell death, cytokine production, and inflammatory response were enriched in *Chrna2*-deficient livers during MASH development. In contrast, fundamental biological processes, such as amino acid metabolism, cellular catabolic process, and response to glucocorticoid, were suppressed in livers without CHRNA2 (Fig 2V). Therefore, unbiased assessment of liver gene expression in GAN diet-fed control and *Chrna2*^fl/fl^;*AlbCre* mice revealed that hepatocyte-specific *Chrna2* deletion affected the hepatic molecular landscape, rendering an aggravated liver damage response during diet-induced MASH development.

### Loss of hepatocyte CHRNA2 signaling increases susceptibility to liver injury

**Fig 3:**
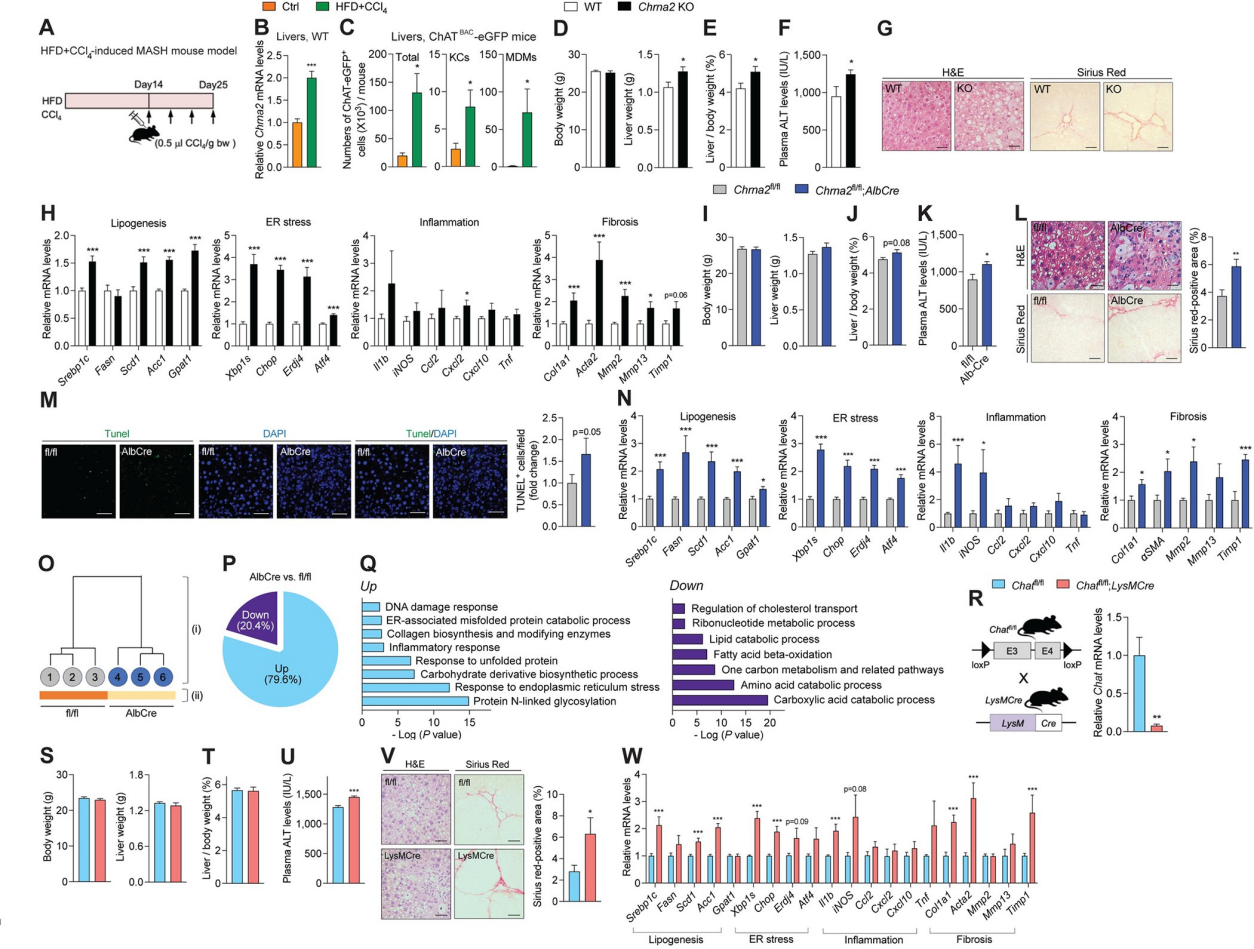
The loss of hepatic CHRNA2 signaling increases susceptibility to liver injury. (A) Schematic diagram illustrating the experimental outline of the development of mouse MASH using HFD feeding combined with carbon tetrachloride (HFD+CCl_4_) administration. (B) qPCR analyses of *Chrna2* expression in liver tissues of WT mice challenged with HFD+CCl_4_ and with control treatment (Ctrl, *n* = 9; HFD+CCl_4_, *n* = 5). (C) Flow cytometric analyses of the abundance of total cells, Kupffer cells (KCs) and MDMs that express ChAT in liver NPCs from ChAT^BAC^-eGFP mice challenged with HFD+CCl_4_ or control treatment (Ctrl, *n* = 6; HFD+CCl_4_, *n* = 4). (D–H) Control WT and whole-body *Chrna2* KO mice treated with HFD+CCl_4_. (D) Left, body weight (WT, *n* = 9; KO, *n* = 7). Right, liver weight (WT, *n* = 9; KO, *n* = 7). (E) Liver per body weight ratio (WT, *n* = 9; KO, *n* = 7). (F) Plasma alanine aminotransferase (ALT; WT, *n* = 10; KO, *n* = 13). (G) HE and Sirius Red staining of liver sections. Scale bars, 50 μm. Representative images are shown. (H) qPCR analyses of MASH pathogenic genes in livers (WT, *n* = 10; KO, *n* = 8). (I–Q) Control *Chrna2*^fl/fl^ and *Chrna2*^fl/fl^;*AlbCre* mice treated with HFD+CCl_4_. (I) Left, body weight (*n* = 6 per group). Right, liver weight (*n* = 6 per group). (J) Liver per body weight ratio (*n* = 6 per group). (K) Plasma ALT (*Chrna2*^fl/fl^, *n* = 13; *Chrna2*^fl/fl^;*AlbCre*, *n* = 8). (L) Left, HE and Sirius Red staining of liver sections. Scale bars, 50 μm. Representative images are shown. Right, quantification of Sirius Red-positive area (*Chrna2*^fl/fl^, *n* = 11; *Chrna2*^fl/fl^;*AlbCre*, *n* = 13). (M) Left, TUNEL staining of liver sections. Scale bars, 50 μm. Right, the relative frequencies of TUNEL-positive cells per field (*Chrna2*^fl/fl^, *n* = 14; *Chrna2*^fl/fl^;*AlbCre*, *n* = 12). Representative images are shown. (N) qPCR analyses of MASH pathogenic genes in livers (*Chrna2*^fl/fl^, *n* = 10; Cre, *n* = 8). (O) Hierarchical (i) and k-means (ii) clustering of hepatic transcriptome assessed with RNA-seq (*n* = 3 per group). The horizontal distance in (i) indicates similarities among each cluster. (P) The relative frequencies of differentially expressed genes from liver RNA-seq (*n* = 3 per group). (Q) Biological pathway analysis of up- or down-regulated genes in *Chrna2*^fl/fl^;*AlbCre* mice relative to controls from liver RNA-seq (*n* = 3 per group). (R) Left, schematic diagram illustrating the generation of macrophage-specific *Chat* deleted mice by crossing *Chat*^fl/fl^ with *LysMCre* mice. Right, qPCR analyses of *Chat* mRNA expression in isolated liver macrophages (*n* = 4 per group). (S–W) Control *Chat*^fl/fl^ and *Chat*^fl/fl^;*LysMCre* mice treated with HFD+CCl_4_. (S) Left, body weight (*Chat*^fl/fl^, *n* = 7; *Chat*^fl/fl^;*LysMCre*, *n* = 6). Right, liver weight (*Chat*^fl/fl^, *n* = 7; *Chat*^fl/fl^;*LysMCre*, *n* = 6). (T) Liver per body weight ratio (*Chat*^fl/fl^, *n* = 7; *Chat*^fl/fl^;*LysMCre*, *n* = 6). (U) Plasma ALT (*Chat*^fl/fl^, *n* = 7; *Chat*^fl/fl^;*LysMCre*, *n* = 6). (V) Left, HE and Sirius Red staining of liver sections. Scale bars, 50 μm. Representative images are shown. Right, quantification of Sirius Red-positive area (*Chat*^fl/fl^, *n* = 7; *Chat*^fl/fl^;*LysMCre*, *n* = 6). (W) qPCR analyses of MASH pathogenic genes in livers (*Chat*^fl/fl^, *n* = 7; *Chat*^fl/fl^;*LysMCre*, *n* = 6). The data underlying the graphs in this figure can be found in S3 Data. Mean ± SEM. *p < 0.05, **p < 0.01, ***p < 0.005 by an unpaired two-sample Student’s *t* test or Mann–Whitney U test. ChAT, choline acetyltransferase; CHRNA2, cholinergic receptor nicotinic alpha 2 subunit; HE, hematoxylin-eosin; HFD, high-fat diet; KO, knockout; MASH, metabolic dysfunction-associated steatohepatitis; MDM, monocyte-derived macrophage; NPC, non-parenchymal cell; WT, wild-type.

One of the key hallmarks of MASH is the development of hepatic fibrosis based on liver cell damage [10]. Although the GAN diet is commonly used to induce SL/MASH, similar to the pathophysiology of human SL/MASH with obesity and insulin resistance, it has been reported to have limitations in inducing liver injuries [22,55]. Additionally, chronic hypercaloric GAN diet-induced significant fat expansion and body weight gain may be confounding factors for MASH development and progression in some cases. Thus, WT mice were next challenged with another MASH-inducing regimen involving exposure to both HFD-feeding and carbon tetrachloride (CCl_4_) treatment, which combines the brief obesogenic metabolic effects of an HFD and the accelerated ramification of CCl_4_ on the induction of liver injury and fibrosis without significant fat and body weight gain (Figs 3A and S6A) [56,57]. We first confirmed activated hepatic acetylcholine-CHRNA2 signaling in this MASH model, indicating its role as a self-defense mechanism against metabolic and injury insult. Increased hepatic *Chrna2* expression levels were observed in the livers of WT mice challenged with an HFD plus CCl_4_ compared to that from control-treated WT mice (Fig 3B). Similarly, the abundance of ChAT-expressing NPCs was significantly higher in livers from ChAT^BAC^-eGFP mice with HFD plus CCl_4_-induced MASH, with a particular contribution of macrophages, including KCs and MDMs, compared to control-treated mice (Figs 3C, S6B and S6C). The expression levels of ChAT in hepatic NPCs, as analyzed by MFI of GFP, were also found to be higher in HFD plus CCl_4_ treatment than in control (S6D Fig). Another reporter model for ChAT, the ChAT-eGFP;*ChATCre*-RFP mouse, confirmed the presence of acetylcholine-producing immune cells, including KCs and MDMs, and their induction in response to HFD plus CCl_4_ by exhibiting overlap between presently active ChAT-eGFP^+^ cells and permanently labeled RFP^+^ cells (S6E Fig). These results are consistent with the notion that the ChAT-CHRNA2 axis is activated during the onset of MASH.

When challenged with the HFD plus CCl_4_ regimen, significantly higher liver-to-body weight ratio and ALT levels were observed in *Chrna2* KO mice compared to control mice with no difference in body weights and food intake (Figs 3D–3F and S6F). Consistent with these findings, histological analyses suggested more lipid accumulation and fibrotic fibers in the livers of *Chrna2* KO mice compared to those of control mice given HFD plus CCl_4_ (Fig 3G). Gene expression analysis revealed that key regulators in lipogenesis, ER stress, inflammation, and fibrogenesis was expressed at a higher level in livers from *Chrna2* KO mice compared to control mice after HFD plus CCl_4_ challenge (Fig 3H). Furthermore, our data using hepatocyte-specific CHRNA2-deficient mice confirmed the direct contribution of hepatic CHRNA2 signaling in HFD plus CCl_4_-induced MASH development. *Chrna2*^fl/fl^*;AlbCre* mice treated with this regimen rendered a more severe MASH-associated phenotype compared to controls, similar to what was observed in global *Chrna2* KO mice. This manifested as increased ALT levels, worsened liver steatosis, Sirius Red-positive area for fibrotic fibers, cell death, and increased MASH-related gene expression without body weight differences, compared to those of the littermate control *Chrna2*^fl/fl^ mice (Fig 3I–3N and S6G). Moreover, our unbiased transcriptome analysis identified a distinct hepatic molecular landscape between the 2 genotypes following HFD plus CCl_4_ treatment, with the majority of DEGs up-regulated in mutant livers relative to controls (Fig 3O and 3P). Biological pathway analysis consistently supported our observation of exacerbated MASH-related phenotypes in the absence of *Chrna2* by identifying down-regulated energy catabolic pathways and up-regulated pathological pathways, such as ER stress and collagen biosynthesis, in mutant livers compared with controls (Fig 3Q). These data generated using the HFD plus CCl_4_ regimen indicate that the loss of hepatocyte CHRNA2 signaling increases susceptibility to liver injury, a major pathogenic feature of MASH, without potential confounding influence due to fat expansion and body weight gain.

Our study identified a subpopulation of hepatic macrophages synthesizing acetylcholine and communicating with hepatocyte CHRNA2 through paracrine signaling. Therefore, we next investigated the physiological significance of acetylcholine-synthesizing macrophages in MASH development. ChAT-eGFP;*LysMCre*-RFP double reporter mice revealed that a significant portion of ChAT-eGFP+ macrophages were labeled with *LysMCre*-driven RFP (S7A Fig). In line with this observation, *Chat*^fl/fl^;*LysMCre* mice demonstrated a robust reduction of *Chat* expression in liver macrophages compared to control *Chat*^fl/fl^ mice, indicating enrichment of *Chat* expression and highly effective *LysMCre*-mediated *Chat* deletion in hepatic macrophages (Fig 3R). In the basal condition, mutant mice did not differ in body weight or liver profiles from controls (S7B–S7F Fig). However, in response to HFD plus CCl_4_ treatment, *Chat*^fl/fl^;*LysMCre* mice exhibited worsened MASH-related parameters, including blood ALT levels, liver steatosis, collagen deposition, and hepatic gene expression levels for lipogenesis and injury, compared to controls, although differences between the 2 genotypes were not as profound as *Chrna2-*deficient animals relative to their corresponding controls (Fig 3S–3W, S7G and S7H). These findings highlight that defective hepatocyte CHRNA2 signaling due to a lack of macrophage-derived acetylcholine increases susceptibility to liver injury.

### Pharmacological inhibition of acetylcholine degradation alleviates MASH phenotypes

**Fig 4:**
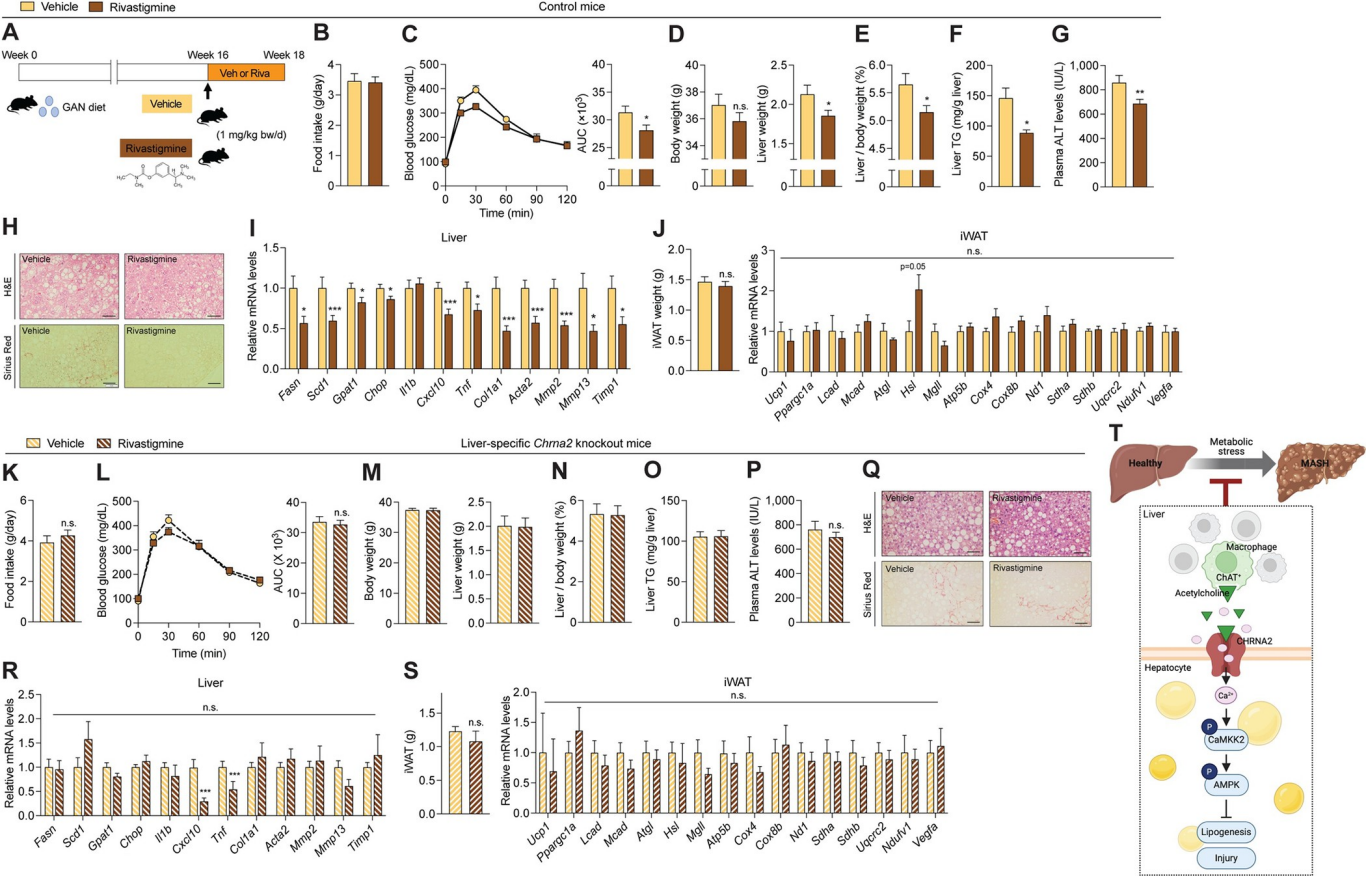
Pharmacological inhibition of acetylcholine degradation ameliorates MASH phenotypes. (A) Schematic diagram illustrating the experimental outline. Gubra-Amylin NASH (GAN) diet-fed mice were treated with vehicle (Veh) or rivastigmine (Riva, 1 mg/kg body weight/day) for 2 weeks. (B–J) GAN diet-fed control mice treated with Veh or Riva. (B) Daily food intake (*n* = 7). (C) GTT (*n* = 8). AUC, area under the curve. (D) Left, body weight (Veh, *n* = 21; Riva, *n* = 26). Right, liver weight (Veh, *n* = 21; Riva, *n* = 26). (E) Liver per body weight ratio (Veh, *n* = 21; Riva, *n* = 26). (F) Liver triglyceride (TG; Veh, *n* = 15; Riva, *n* = 12). (G) Plasma alanine aminotransferase (ALT; Veh, *n* = 27; Riva, *n* = 21). (H) HE and Sirius Red staining of liver sections. Scale bar, 50 μm. Representative images are shown. (I) qPCR analyses of MASH pathogenic genes in the liver (Veh, *n* = 21; Riva, *n* = 22). (J) Left, iWAT weight (Veh, *n* = 8; Riva, *n* = 6). Right, qPCR analyses of adaptive thermogenic genes in iWAT (Veh, *n* = 8; Riva, *n* = 6). (K–S) GAN diet-fed liver-specific *Chrna2* KO mice treated with Veh or Riva. (K) Daily food intake (*n* = 16). (L) GTT (Veh, *n* = 7; Riva, *n* = 8). AUC, area under the curve. (M) Left, body weight (Veh, *n* = 15; Riva, *n* = 14). Right, liver weight (Veh, *n* = 15; Riva, *n* = 14). (N) Liver per body weight ratio (Veh, *n* = 15; Riva, *n* = 14). (O) Liver TG (Veh, *n* = 16; Riva, *n* = 17). (P) Plasma ALT (Veh, *n* = 27; Riva, *n* = 39). (Q) HE and Sirius Red staining of liver sections. Scale bars, 50 μm. Representative images are shown. (R) qPCR analyses of MASH pathogenic genes in the liver (Veh, *n* = 13; Riva, *n* = 11). (S) iWAT weight (*n* = 7 per group). Right, qPCR analyses of adaptive thermogenic genes in iWAT (*n* = 7 per group). (T) A proposed model of CHRNA2-mediated liver-protective effects against MASH. The data underlying the graphs in this figure can be found in S4 Data. Mean ± SEM. n.s., not significant. *p < 0.05, **p < 0.01, ***p < 0.005 by an unpaired two-sample Student’s *t* test or Mann–Whitney U test. GTT, glucose tolerance test; HE, hematoxylin-eosin; iWAT, inguinal white adipose tissue; MASH, metabolic dysfunction-associated steatohepatitis.

The above data suggest that hepatocyte CHRNA2 signaling offers at least partial protection against diet-induced MASH development. Thus, we wanted to test if further activation of this defense pathway would offer greater protection against MASH. Thus, we next adopted a gain-of-function approach using rivastigmine, a pharmacological inhibitor of acetylcholine degradation, to activate cholinergic signaling in diet-induced MASH animal models (Fig 4A). Such inhibitors, including rivastigmine, have been used clinically for treating neurodegenerative diseases for decades [58,59]. While no significant differences in food intake were observed, suggesting no appetite change (Fig 4B), improved glucose tolerance after rivastigmine treatment demonstrated the drug treatment has metabolically beneficial effects on mice fed a GAN diet (Fig 4C). Trending lower body weights and statistically significantly lower liver mass, independent of the body weight change, were seen in rivastigmine-treated mice compared to control-treated mice (Fig 4D and 4E). Rivastigmine treatment resulted in significantly lower hepatic TG content and plasma ALT levels in the animals developing MASH, compared to vehicle-control mice, suggesting that drug treatment ameliorated MASH-associated steatosis and liver damage (Fig 4F and 4G). Consistent with these results, liver morphology examination suggested less lipid-laden hepatocytes and fibrosis in MASH mice after rivastigmine treatment compared to controls (Fig 4H). Likewise, rivastigmine treatment was associated with significantly lower hepatic transcriptional activation of major genes involved in MASH pathogenesis (Fig 4I). However, no significant changes in iWAT weight or thermogenic gene expression were observed after 2 weeks of rivastigmine treatment (Fig 4J), suggesting that acute inhibition of acetylcholine degradation is insufficient to fully activate adipose CHRNA2 signaling, particularly in obesity, when beige adipocyte activity is repressed. We observed similar beneficial outcomes from rivastigmine treatment in WT mice fed a chronic HFD, suggesting that rivastigmine may be efficacious as both a treatment for MASH development and as an early intervention to mitigate the risk of MASH development (S8A–S8D Fig). In contrast, in chow diet-fed mice, when the acetylcholine-CHRNA2 adaptive signaling is not activated under healthy conditions, the effects of rivastigmine were almost absent in liver (S8E–S8H Fig).

In contrast to WT mice, when GAN diet-fed hepatocyte-specific *Chrna2* KO (*Chrna2*^fl/fl^;*AlbCre*) mice were treated with rivastigmine, minimal beneficial effects of rivastigmine on both systemic metabolism and hepatic function were observed (Fig 4K–4Q). Liver gene expression analyses showed that except for some inflammatory genes, most of the genes that drive MASH-related phenotypes had similar expression patterns between vehicle and rivastigmine groups (Fig 4R). Consistent with the observation in control animals, rivastigmine treatment for hepatocyte CHRNA2 activation did not alter iWAT mass and thermogenic activation at the transcriptional level in mutant animals fed the GAN diet (Fig 4S). In addition, BAT, where there is no functional CHRNA2 signaling, did not show any unexpected thermogenic responses to rivastigmine in both control and *Chrna2*^fl/fl^;*AlbCre* mice fed the GAN diet (S9 Fig). We further confirmed that hepatocyte CHRNA2-dependent liver-protective effects of rivastigmine were reproduced in HFD plus CCl_4_-induced MASH models (S10 Fig). These results indicate that the protective effects of rivastigmine against MASH development are primarily mediated via hepatocyte CHRNA2 signaling. Collectively, these results show that hepatocyte CHRNA2 signaling is manipulable and hence, may hold therapeutic potential to treat MASH.

## Discussion

The nAChRs have received recent attention as drug targets for many human diseases due to their rich diversity in expression patterns and biological function [12]. Our study suggests that a hepatic acetylcholine-CHRNA2 signaling axis comprised of local acetylcholine-secreting macrophages and hepatocytes, senses hypercaloric metabolic stress and is stimulated through CaMKK2-AMPK signaling to defend against the development of MASH (Fig 4T). Importantly, this newly identified hepatic signaling pathway is physiologically significant, as the loss of hepatic CHRNA2 expression exacerbated MASH pathophysiology in 2 different mouse models. In contrast, enhancing this signaling pathway via rivastigmine treatment ameliorated hepatic pathologies and whole-body metabolic dysfunction in mice developing MASH.

We adopted 2 different MASH models in the study: GAN and HFD plus CCl_4_. The GAN diet mimics fast food style diets with high-fructose, high-fat, and high-cholesterol [60]. In preclinical animal studies, the GAN diet is widely used to develop SL/MASH presenting similar pathophysiology of human SL/MASH with obesity and insulin resistance. Although WT mice (e.g., C57Bl/6 J) need to be fed with the GAN diet for ≥38 weeks to develop advanced form of fibrosing MASH, ≤20 weeks have been reported to show MASH features and be implicated in the MASH study [22,43,61]. We further examined the roles of hepatocyte CHRNA2 signaling in a diet-induced MASH model with accelerated liver injury and no significant fat expansion using brief HFD feeding combined with CCl_4_ challenge [57]. CCl_4_ has been used for decades to induce liver fibrosis and injury in animals [56]. Multiple injections of CCl_4_ with an HFD cause oxidative stress that induces apoptosis and inflammation, leading to the development of fibrosis in mice [62]. In both GAN diet- and HFD plus CCl_4_-induced MASH models, *Chrna2*-deficient mice exhibited higher fibrogenic gene expression in livers than control counterparts. It is of note that in *Chrna2* KO mice, more anti-fibrogenic *Mmp2* expression levels were observed, which may reflect and result from abnormally increased collagen synthesis and MMP inhibitor activation, as indicated by elevated *Col1a1* and *Timp1* expression, contributing to simultaneous degradation and turnover of the extracellular matrix [63–65].

The cell type- and/or subunit-specific biological roles of nAChRs have not been fully defined, particularly in nonneuronal systems. In the liver, CHRNA7 has been relatively well studied among nAChRs. Similar to *Chrna2*, *Chrna7* expression is up-regulated in murine fatty livers, and its genetic deletion aggravates MASH phenotypes via exacerbating hepatic nerve activated inflammation in KCs, in mice fed MASH-inducing diets, such as HFD and methionine-choline-deficient diet [13–15]. In hepatocytes, muscarinic acetylcholine receptors, specifically M3, showed protective effects against metabolic stress by reducing lipid accumulation in an in vitro system [31]. However, its up-regulation in stellate cells is associated with fibrosis in the liver, indicating a cell type-dependent role of hepatic cholinergic signaling in MASH [66]. Our data revealed that CHRNA2 signaling is functional in the parenchymal cells (specifically, hepatocytes) underlying metabolic adaptation in response to excess calories and MASH development. The findings were verified using newly generated hepatocyte-restricted CHRNA2 KO mice. While rivastigmine treatment improved the MASH pathophysiology in mice fed an obesogenic diet, the effects were minimal when CHRNA2 was absent in hepatocytes. However, transcriptional activation of hepatic pro-inflammatory genes remained suppressed by rivastigmine treatment regardless of hepatocyte *Chrna2* deletion, suggesting this effect may occur via other nAChRs, such as CHRNA7 in KCs [15].

Although previous studies have shown the crucial role of parasympathetic tone in the regulation of hepatic metabolism [67,68], the presence of parasympathetic nerves, the neuronal source of acetylcholine, in liver, has been debated due to conflicting experimental results using histochemistry or 3D imaging approaches [16–18]. Moreover, hepatic denervation or liver transplantation did not lead to serious metabolic aberration in animals or humans, at least without metabolic challenges [19–21]. It is conceivable that acetylcholine secreted from a local nonneuronal source serves as an agonist for nAChRs expressed in neighboring cells to maintain homeostasis in peripheral tissues [34,69]. Recent evidence strengthens our finding of acetylcholine-producing immune cells within the liver and their role in activating hepatocyte nAChR signaling [70]. However, it highlights the need for dissecting subunit-specific biological roles of nAChRs in response to the local agonist, considering the complexity and heterogeneity of MASH pathogenesis in different conditions, by indicating hepatic CHRNA4 as an accelerator of MASH progression [70].

Accumulating evidence indicates the therapeutic potential of the nonneuronal cholinergic system for many disorders as its dysregulation is associated with the pathogenesis of various diseases [35,36]. Here, our study revealed that a population of macrophages that express ChAT secrete acetylcholine to activate hepatocyte CHRNA2 signaling and that the ChAT-CHRNA2 axis serves as a defense mechanism to dampen the progression of MASH and may hold therapeutic potential.

## Materials and methods

### Animals

All animal studies were reviewed and approved by the Institutional Animal Care and Use Committee at the University of Michigan (protocol number, PRO00010791). Ai14 (007914), C57BL/6J (000664), *Chrna2* KO (005797), ChAT^BAC^-eGFP (007902), *Chat*^fl/fl^ (016920), *Chat*Cre (031661), *LysM*Cre (004718), *Vav-iCre* (008610*)*, and *AlbCre* (003574) mice were obtained and genotyped using the PCR protocols from the Jackson Laboratory. *Chrna2*Cre mice were originally generated by Dr. Klas Kullander (Uppsala University, Sweden) [71]. *Chrna2*^fl/fl^ mice were generated using CRISPR/Cas9 gene editing and genotyped using the PCR primers designed to detect the inserted LoxP sites as described previously [46]. Littermate floxed mice were controls for co-housed Cre-loxP system-mediated conditional gene knockout animals. As control animals for *Chrna2* KO mice, either WT C57BL/6J or littermate WT controls were used. Mice were maintained under 12 h light/12 h dark cycle on a standard rodent chow diet (5L0D, PicoLab). Seven- to ten-week-old mice were fed a western diet containing 40% fat, 20% fructose, 10% sucrose, 2% cholesterol (GAN diet; D09100310, Research Diets) for 18 weeks or otherwise indicated, similar to previously reported protocols shown to induce MASH [22,43,55,60,61]. In a separate MASH-inducing protocol, 7- to 10-week-old mice were fed an HFD containing 60% of calories from fat (D12492, Research Diets) for 4 weeks with intraperitoneal injections of CCl_4_ at a dose of 0.5 μl/g of body weight in the third and fourth weeks of HFD feeding (twice per week), combining metabolic challenges from HFD and chemical damage from CCl_4_ treatment, adapted from previous studies [56,57,62]. Mice were also fed an HFD for 11 or 18 weeks to develop obesity and MASH.

### Isolation of primary cells

Primary mouse hepatocytes were isolated and cultured as previously reported [72]. In brief, livers were perfused with washing buffer (HBSS buffer containing 0.5 mM EGTA and 25 mM HEPES (pH 7.4)) and digestion medium (DMEM-low glucose containing 200 mg/L CaCl_2_, 1% penicillin/streptomycin, 15 mM HEPES and 100 U/mL collagenase IV). After digestion, livers were excised, dissociated in digestion medium, and filtered through a 70-μm cell strainer. The cell suspension was centrifuged, and the pellet and supernatant were separated to isolate hepatocytes and NPCs, respectively. The pellet, containing hepatocytes, was washed twice with isolation medium (DMEM/F12 GlutaMAX supplemented with 10% FBS, 1% penicillin/streptomycin, 1 μm dexamethasone, and 0.1 μm insulin). Isolated hepatocytes were stained with trypan blue to count the number of live cells. The cells then were plated onto collagen-coated culture plates and incubated in isolation medium for an hour. Non-adherent cells were removed, and culture medium (DMEM-low glucose containing 10% FBS, 1% penicillin/streptomycin, 0.1 μm dexamethasone, and 1 nM insulin) was added. After 3 h, hepatocytes were maintained in culture medium without FBS until further treatment with nicotine, palmitate or LPS. Nicotine or LPS was dissolved in PBS; thus, PBS was used as a control vehicle. Palmitate was conjugated with BSA (6:1 molecular ratio), and thus control group was incubated with BSA [73]. Mouse liver NPCs were isolated according to the method reported previously with minor modifications [74,75]. The supernatant collected from fractionation containing NPCs was centrifuged at 500 × *g* for 10 min. The NPC pellet was suspended in 10 ml NPC washing buffer (PBS supplemented with 0.5% FBS) and centrifuged using Percoll gradient media (25% and 50%, v/v) at 1,800 × *g* for 20 min with the brake off. NPCs located within the middle layers between 25% and 50% Percoll gradient were collected and subjected to erythrocytes lysis using eBioscience RBC Lysis Buffer. NPCs were washed with NPC washing buffer for culture or with PBS for acetylcholine quantification. To generate NPCs-CM, NPCs isolated from chronic GAN diet-fed WT mice were cultured with rivastigmine for 16 h, and the medium was collected and centrifuged. Primary Kupffer cells from NPCs were cultured as previously described in RPMI 1640 supplemented with 10% FBS and 1% penicillin/streptomycin [74,75].

BMDMs were isolated and cultured as previously reported [76,77]. Briefly, cells were flushed from the femur and tibia of 6- to 10-week-old mice using cold PBS then centrifuged at 500 × *g* to pellet bone marrow cells. Erythrocytes were lysed using Red Blood Cell Lysis Buffer (Sigma). Cells were grown on non-tissue culture-treated sterile petri dishes in 80% v/v DMEM/F-12 GlutaMAX medium supplemented with 20% v/v conditioned medium from L929 cells. After 5 to 7 days, cells were seeded into 6-well plates for experiments.

### Human cell cultures

Human hepatoma cell line HepG2 and human embryonic kidney 293T cells were obtained from ATCC. HepG2 and 293T cells were grown in DMEM containing 10% FBS and 1% penicillin/streptomycin. Primary human hepatocytes and culture media were purchased from XenoTech. The cells were thawed and plated into 12-well plates for experiments according to the instruction provided.

### Lipid droplet staining

To stain lipid droplets, cultured hepatocytes isolated from the chow diet-fed control mice and GAN diet-fed MASH mice were fixed in 10% formalin for 30 min at room temperature. Fixed cells were stained with Oil Red O staining working solution as described previously [78]. Or the fixed cells were washed with PBS twice before incubated with 1 μg/ml fluorescent neutral lipid dye, 4,4-Difluoro-1,3,5,7,8-Pentamethyl-4-Bora-3a,4a-Diaza-s-Indacene (BODIPY 493/503). After 30-min incubation, cells were washed with PBS, counterstained with DAPI. Images were taken using an inverted Leica DMIRB microscope.

### Quantitative real-time PCR analysis (qPCR)

Total RNA was isolated using the TRIzol Reagent (Sigma) following the manufacturer’s instructions. An equal amount of RNA from each sample was reverse-transcribed using for M-MLV Reverse Transcriptase (Life Technologies). The synthesized cDNA was subjected to real-time PCR using SYBR Green (Thermo Fisher Scientific). Relative mRNA expression was calculated with the *ΔΔ*C_T_ method and normalized to internal control *Tbp* that showed comparable expression levels between genotypes or conditions. The qPCR primer sequences are listed in S2 Table.

### Calcium imaging assay

Primary mouse hepatocytes, primary human hepatocytes, or HepG2 cells were plated on collagen-coated glass bottom culture plates and maintained as described. The cells were incubated with 10 μm Fura 2-AM at 37°C for 30 min and then washed with standard Tyrode’s solution (135 mM NaCl, 4 mM KCl, 10 mM glucose, 10 mM HEPES, 2 mM CaCl_2_, and 1 mM MgCl_2_ (pH 7.4)). Calcium imaging assays were conducted using an Olympus IX73 invert microscope under a 40× objective. Fluorescence signals were recorded upon sequential excitation with 340 nm and 380 nm using an ORCA-Flash 4.0 sCMOS camera (Hammatsu). After establishing a baseline 340/380 nm ratio, the CHRNA2 agonist (500 μm nicotine or 100 μm acetylcholine) was perfused onto the cells with or without EGTA. Data were processed with MetaFluor software (Molecular Devices).

### Luciferase reporter assay

*Chrna2* reporter plasmid was constructed as previous reported [37]. Briefly, 5 kb of mouse *Chrna2* promoter region was amplified by PCR. The PCR product was digested by *MluI* and *XhoI* and then cloned into pGL3 basic vector (Promega) upstream of firefly luciferase coding sequence. The *Chrna2* reporter plasmid was confirmed by DNA sequencing, and 293T cells were seeded in 24-well plate and then transfected with 400 ng of c-Flag pcDNA3 (Addgene) or pcDNA3 *HIF1A*, 90 ng of *Chrna2* reporter plasmid and 10 ng of *Renilla* luciferase plasmid using linear polyethylenimine (Thermo Fisher Scientific). Two days post-transfection, luciferase activity was determined with Dual-Luciferase Report Assay Kit (Promega) according to the manufacturer’s instruction. Firefly luciferase activity was determined by a PerkinElmer 2300 EnSpire microplate reader and normalized to *Renilla* luciferase activity.

### Immunoblotting analysis

Total cell lysates were prepared with ice-cold RIPA buffer (50 mM Tris-HCl (pH 7.5), 1% Triton X-100, 1% sodium deoxycholate, 0.1% SDS, 150 mM NaCl, 1 mM phenylmethylsulfonyl fluoride) containing a protease inhibitor cocktail (Roche) and phosphatase inhibitors (10 mM NaF, 60 mM β-glycerophosphate (pH 7.5), 2 mM sodium orthovanadate, and 10 mM sodium pyrophosphate). Similarly, liver tissues were homogenized in ice-cold RIPA buffer containing phosphatase and protease inhibitors. Proteins were separated on SDS-PAGE and immunoblotted with the following primary antibodies (S3 Table): phospho-AMPKα^T172^ (1:1,000), AMPKα (1:1,000), phospho-CaMKK2^S511^ (1:1,000), phospho-JNK1/2^T183/Y185^ (1:1,000), JNK1/2 (1:1,000), phospho-NF-κB p65^S536^ (1:1,000), NF-κB p65 (1:1,000), RFP (1:1,000), SREBP1 (1:1,000), α-Tubulin (1:2,000), β-Actin (1:5,000), and HSP90 (1:1,000).

### Flow cytometry and sorting

To isolate hepatic NPCs, livers were mechanically homogenized in FACS buffer (PBS, 2% FCS, 1 mM EDTA), then passed through a 100-μm strainer to remove large debris. Homogenates were centrifuged twice at 50 × *g* and the supernatant carried over each time, to remove hepatocytes. Supernatants were then spun at 500 × *g* and the pellet retained. NPCs were isolated by density centrifugation at 1,500 × *g* with the brake off, using 25% OptiPrep Density Gradient Medium, then washed in FACS buffer ready for downstream use. For flow cytometric analysis of BMDM, cells were gently lifted using TrypLE Express (Thermo Fisher Scientific), then washed with FACS buffer prior to staining. Cells for flow cytometry were preincubated with TruStain FcX PLUS (Biolegend) to block nonspecific binding then cells were incubated with a cocktail of fluorescently conjugated antibodies (S3 Table) at 4°C in the absence of light for 30 min. TO-PRO-3 (Thermo Fisher Scientific) was used as a viability stain. Fluorescence-minus-one (FMO) controls were used to determine true positive populations and to identify ChAT-eGFP+ or *Chat*Cre;RFP+ cells, while autofluorescence was gated out using an open channel (excitation 488 nm, emission 710/50 nm). UltraComp eBeads (Invitrogen) were used for single-stained compensation controls. A BD LSR Fortessa was used for flow cytometry analysis and a BD FACS Aria III (100 μm nozzle) was used for cell sorting, in which cells were sorted into FACS buffer before transfer to TRIzol for RNA isolation. Magnetic sorting of liver macrophages from a single cell suspension of NPCs was performed using mouse anti-F4/80-microbeads (Miltenyi Biotec) through an LS column. Magnetically sorted cells were collected in TRIzol for RNA isolation. Data were acquired with FACSDiva software (BD Biosciences) and analyzed using FlowJo v10.6.1 (TreeStar/BD Biosciences).

### Acetylcholine quantification

Freshly isolated hepatic NPCs from mice were incubated in PBS containing 150 μm rivastigmine for 30 min at room temperature. Acetylcholine (Ach) was measured using liquid chromatography-tandem mass spectrometry (LC-MS/MS) similar to previously described [37]. Standard solutions of acetylcholine were prepared in 250 μm ascorbic acid in water to create a calibration range of 0.25 to 125 nM. Calibration curves were prepared based on the peak area ratio of the standard to the internal standard by linear regression. A deuterium-labeled internal standard [d4Ach (C/D/N isotopes, Pointe-Claire, Canada)] was added to samples and standards, diluted 1:3 (v/v) in water, and centrifuged for 10 min at 12,100 × *g*. The supernatant was transferred to an HPLC vial and analyzed as described below. All samples and standards were analyzed in triplicate using a Phenomenex Kinetex C18 chromatography column (100 × 2.1 mm, 1.7 μm, 100Å) on a Vanquish ultrahigh-pressure liquid chromatograph (Thermo Fisher Scientific, Gemering, Germany) interfaced to a TSQ Quantum Ultra triple quadrupole mass spectrometer (Thermo Fisher Scientific, San Jose, California, United States of America). Mobile phase A was 10 mM ammonium formate with 0.15% (v/v) formic acid in water. Mobile phase B was acetonitrile. The gradient used was as follows: initial, 5% B; 0.60 min, 8% B; 0.68 min, 26% B; 1.05 min, 75% B; 1.8 min, 100% B; 2.0 min, 100% B; 2.2 min, 5% B; 3.0 min, 5% B at 600 μl/min. The sample injection volume was 5 μl. The autosampler was kept at ambient temperature, and the column was held at 30°C in still air mode. Electrospray ionization was used in positive mode at 4 kV. The capillary temperature was 400°C, the vaporizer temperature was 350°C, the sheath gas was 10, and the auxiliary gas was 5. Ach ions were detected in MS/MS mode with the following transitions (*m/z*): (Ach) product: 146, precursor: 87; (d4-Ach) product: 150, precursor: 91. Tube lens and collision energy was 53 and 13, respectively. Automated peak integration was performed using XCalibur 3.0 MS software. All peaks were visually inspected to ensure proper integration.

### Metabolic analyses

Plasma alanine aminotransferase (ALT) concentrations and plasma/liver triglyceride (TG) levels were determined using ALT Colorimetric Activity Assay Kit (Cayman) and Triglyceride Colorimetric Assay Kit (Cayman), respectively, according to the provided protocols. Plasma insulin levels were measured using an insulin ELISA kit (CrystalChem). For glucose tolerance tests (GTT), mice were fasted for 16 h and intra-peritoneally injected with a glucose solution at 1.5 g/kg body weight. For oral glucose tolerance tests (OGTTs), mice were fasted for 6 h and orally given a glucose solution at 1.5 g/kg body weight. Glucose levels were measured from tail blood before and 15, 30, 60, 90, and 120 min after glucose injection/gavage using the OneTouch Ultra Glucometer (LifeScan). Body temperature was measured using an RT-3 mouse rectal probe (World Precision Instruments).

### HE and Sirius Red staining

Liver tissues were dissected and fixed immediately in 10% formalin at 4°C overnight. Routine paraffin-embedding and hematoxylin-eosin (HE) staining were conducted by the University of Michigan Rogel Cancer Center Research Histology and Immunohistochemistry Core. To examine liver fibrosis, the formalin-fixed and paraffin-embedded liver sections were stained with Picrosirius (Sirius) Red using Picrosirius Red Stain Kit (Polysciences) according to the manufacturer’s instruction. Images were captured using a LEICA DM2000. Quantification of the Sirius Red-stained collagen was done using ImageJ software by staff scientists in a blinded fashion.

### Fluorescence microscopy

Fixed liver tissue was washed by PBS and put into 30% sucrose at 4°C overnight. Then, the samples were frozen in Tissue-Tek O.C.T. compound (Fisher). The frozen liver was cut into 5-μm sections and mounting with ProLong Gold Antifade Reagent with 4′,6-diamidino-2-phenylindole (DAPI, Invitrogen). Fluorescent signal was visualized using a Leica SP8 confocal microscope.

### Terminal deoxynucleotidyl transferase dUTP nick end labeling (TUNEL) assay

Detection of individual apoptotic cells in formalin-fixed liver sections was performed using In Situ Cell Death Detection Kit, Fluorescein (Roche). Briefly, paraffin-embedded liver sections were deparaffinized, rehydrated, and antigen retrieved. The DNA strand breaks labeling was conducted following the manufacturer’s instructions. Sections were counterstained with DAPI (Vector Laboratories). The pictures of TUNEL-positive cells were obtained by using a LEICA DM2000 fluorescence microscope and analyzed using the Olympus cellSens Standard software. At least 10 different areas per sample were counted for TUNEL-positive cells by 2 staff scientists in a blinded fashion.

### RNA sequencing (RNA-seq)

RNA-seq library preparation, high-throughput sequencing, and the bioinformatics pipeline were conducted by the Advanced Genomics Core at the University of Michigan. Total RNA was isolated from livers of GAN diet fed or HFD plus CCl_4_ challenged *Chrna2*^fl/fl^ and *Chrna2*^fl/fl^;*AlbCre* mice and used to generate sequencing libraries with NEBNext Poly(A) mRNA Magnetic Isolation Module (NEB, E7490) and NEBNext Ultra II Directional RNA Library Prep Kit (NEB, E7760). The quality of prepared libraries was validated using Agilent TapeStation. Paired-end (151 bp) reading was performed by an Illumina NovaSeq (S4). Bcl2fastq2 Conversion Software (Illumina) was used to generate de-multiplexed Fastq files. Raw reads were mapped to the reference genome GRCm38 (ENSEMBL) using STAR v2.7.8a and converted to FPKM (fragments per kilobase of exon per million fragments mapped) using RSEM v1.3.3. Unbiased group clustering was achieved and visualized by Uniform Manifold Approximation and Projection (UMAP) (BioVinci), Hierarchical Clustering or k-means Clustering (Morpheus software, Broad Institute). To identify differentially expressed genes (*P* < 0.05) and calculate relative gene expression between *Chrna2*^fl/fl^ and *Chrna2*^fl/fl^;*AlbCre* mice, a pseudocount of 0.1 was added to FPKM of all genes. Differentially up-regulated (a fold change of >3 or a fold change of >1) or down-regulated (a fold change of <1) genes in *Chrna2*^fl/fl^;*AlbCre* mice relative to controls were analyzed for pathway enrichment using the Database for Annotation, Visualization and Integrated Discovery (DAVID Bioinformatics) or Metascape [79].

### Statistics

All data are presented as means ± standard error of the mean (SEM). The results shown are representative of at least 2 independent experiments. Sample sizes are biological replicates and were determined based on preliminary data or previously published literature. Statistical differences between 2 groups were analyzed by normality tests, including the Shapiro–Wilk test and the D’Agostino–Pearson omnibus test, followed by an unpaired two-sample Student’s *t* test or Mann–Whitney U test. Prism 8 software (GraphPad) or Office Excel (Microsoft) was used to graph and statistically analyze the data.

## Supporting information

S1 Fig Expression and regulation of *Chrna2* in the liver. Related to Fig 1. (A) qPCR analyses of *Chrna2* expression in various murine tissues (*n* = 4 per group). (B) Expression levels of nicotinic and muscarinic acetylcholine receptor subunits (nAChRs and mAChRs) in mouse livers from transcriptome data from NCBI Gene Expression Omnibus dataset GSE199121 (*n* = 3, normal control group from this study). *Chrna8* is not listed in the figures since it has only been detected in chicken. (C) qPCR analyses of hepatic MASH pathogenic genes in wild-type (WT) mice fed a chronic GAN or chow diet (*n* = 5 per group). (D–F) Human tissue transcriptome data of *CHRNA2* from the NCBI Gene Expression Omnibus database. (D) GSE2004 dataset [CNH (Children’s National Medical Center); positive control (P-ctrl, universal human reference RNA), *n* = 3; kidney, *n* = 4; liver, *n* = 3; spleen, *n* = 3; TGRI (Translational Genomics Research Institute), *n* = 3]. (E) GSE15653 dataset (livers; healthy lean subjects, *n* = 5; obese subjects, *n* = 4). (F) GSE23343 dataset (livers; normoglycemic participants, *n* = 7; type 2 patients with diabetes, *n* = 10). (G) qPCR analyses of *Chrna2* and hepatocyte marker *Ck18* in primary liver non-parenchymal cells (NPCs) and hepatocytes from WT mice (NPCs, *n* = 5; hepatocytes, *n* = 6). (H) qPCR analyses of genes encoding nAChR subunits in mouse primary hepatocytes (*n* = 6). (I) qPCR analyses of *Chrna2* expression in liver tissues and primary hepatocytes from control WT and whole-body *Chrna2* knockout (KO) mice (liver, *n* = 4; hepatocytes, *n* = 6). (J) Intracellular calcium uptake mediated by CHRNA2 agonist acetylcholine (Ach, 100 μm) in the presence or absence of calcium in primary hepatocytes from control WT and *Chrna2* KO mice (WT, *n* = 12; KO, *n* = 14). (K) Intracellular calcium uptake mediated by 100 μm Ach in primary human hepatocytes (*n* = 23). (L) Intracellular calcium levels in human hepatoma cell line HepG2 with 500 μm nicotine (Nic) (*n* = 13) or 100 μm Ach (*n* = 15) stimulation. (M) qPCR analyses of *CHRNA2* expression in HepG2 cells treated with vehicle (Ctrl), palmitate (0.2 mM for 16 h; *n* = 5 per group), or lipopolysaccharides (LPS, 100 ng/ml for 16 h; *n* = 4 per group). (N) Left, qPCR analyses of lipogenic markers in vehicle (Ctrl) or Nic (2 mM for 6 h)-treated primary hepatocytes isolated from GAN diet-fed *Chrna2* KO mice (Ctrl, *n* = 5; Nic, *n* = 6). Right, immunoblot analyses for phosphorylated and total AMPK in primary hepatocytes of GAN diet-fed *Chrna2* KO mice after 2 mM nicotine (Nic) treatment for the time indicated or 1 mM 5-aminoimidazole-4-carboxamide riboside (AICAR) treatment for 30 min as a positive control (AMPK activator). β-Actin was used as the loading control. The data underlying the graphs in this figure can be found in S5 Data and S2 Raw Images. Mean ± SEM. n.d., not detected. n.s., not significant. *p < 0.05, **p < 0.01, ***p < 0.005 by an unpaired two-sample Student’s *t* test.
(TIF)

S2 Fig Identification and activation of ChAT-expressing hepatic immune cells. Related to Fig 1. (A) qPCR analyses of *Chat* and immune marker *Cd45* in primary hepatocytes and non-parenchymal cells (NPCs) from wild-type (WT) mice (NPCs, *n* = 5; hepatocytes, *n* = 6). (B) Immune profiles of ChAT-expressing cells in mouse hepatic NPCs using flow cytometric analysis. Gating strategy for surface marker-based immunophenotyping of ChAT-eGFP^+^ NPCs. Live singlets (TOPRO3^-^) were gated on for ChAT-eGFP^+^ cells. ChAT-eGFP^+^ cells were defined as CD45^+^ or CD45^-^ (i), and CD45^+^ cells gated on for monocyte-derived macrophages (MDMs; ii, CD11b^hi^ F4/80^lo^) and Kupffer cells (KCs; iii, CD11b^lo^ F4/80^hi^). Non-myeloid cells (CD11b^-^ F4/80^-^) were defined broadly as CD3^+^ T cells or CD19^+^ B cells. CD3^+^ T cells were then defined as TCRγδ^+^ (γδ T cells, iv) or TCRβ^+^ (αβ T cells), which were further broken down into CD8^+^ (v), CD4^+^ (vi), or double negative (DN) αβ T cells (vii). CD19^+^ B cells were defined as B220^+^(viii) or B220^-^ (ix). The frequencies of each subpopulation are listed in S1 Table. (C) Representative gating strategy for identification of neutrophils (CD45^+^ CD11b^+^ Ly6G^+^) and non-neutrophils in livers from chow diet-fed and Gubra-Amylin NASH (GAN) diet-fed mice. No significant presence of ChAT-eGFP^+^ neutrophils was detected in the liver. (D) Flow cytometric analyses of the abundance of total cells, KCs and MDMs that express ChAT in liver NPCs from ChAT^BAC^-eGFP mice fed with chow diet or GAN diet. Cell numbers were normalized with liver weights (chow, *n* = 10; GAN, *n* = 6). (E) Flow cytometric analyses of normalized abundance of total KCs and MDMs with liver weights from WT mice fed with chow diet or GAN diet (chow, *n* = 8; GAN, *n* = 10). (F) ChAT-eGFP median fluorescence intensity (MFI) for ChAT-eGFP^+^ cells (chow, *n* = 10; GAN, *n* = 6). (G) qPCR analyses of *Chat* in primary WT mouse KCs treated with vehicle, 0.5 mM palmitate for 18 h (left; *n* = 6 per group) or 10 ng/ml LPS for 4 h (right; Ctrl, *n* = 8; LPS, *n* = 10). (H) Flow cytometry histograms of ChAT-eGFP^+^ bone marrow-derived macrophages (BMDM). Left, BMDM treated with vehicle (blue) or 0.5 mM palmitate for 7 h (orange) or 18 h (red). Right, BMDM treated for 4 h with vehicle (Ctrl; blue) or lipopolysaccharides (LPS) at 50 ng/ml (orange) or 100 ng/ml (red). The data underlying the graphs in this figure can be found in S6 Data. Mean ± SEM. *p < 0.05, **p < 0.01, ***p < 0.005 by an unpaired two-sample Student’s *t* test or Mann–Whitney U test.
(TIF)

S3 Fig Hepatic CHRNA2 signaling in livers of high fat diet-fed mice. Related to Fig 1. (A) qPCR analyses of hepatic *Chrna2* expression in wild-type (WT) mice fed with a control chow diet or a high-fat diet (HFD, 11 weeks; chow, *n* = 9; *n* = 8, HFD). (B) Flow cytometric analyses for the abundance of total non-parenchymal cells that express ChAT in livers from ChAT^BAC^-eGFP mice fed chow diet or HFD for 11 weeks (chow; *n* = 5; HFD, *n* = 3). (C) Primary mouse hepatocytes with elevated metabolic stress signaling related to MASH were isolated from WT mice fed with HFD for 18 weeks. Left, qPCR analyses of lipogenic genes in vehicle (Ctrl) or Nic (2 mM for 6 h)-treated hepatocytes (*n* = 6 per group). Right, immunoblot analyses for phosphorylated and total AMPK in hepatocytes exposed to 2 mM Nic for the time indicated. The data underlying the graphs in this figure can be found in S7 Data and S3 Raw Images. Mean ± SEM. *p < 0.05, ***p < 0.005 by an unpaired two-sample Student’s *t* test.
(TIF)

S4 Fig Phenotype of whole-body CHRNA2-deficient mice under basal or MASH condition. Related to Fig 2. (A–E) Control wild-type (WT) and whole-body *Chrna2* KO mice fed a chow diet. (A) Body weight (WT, *n* = 6; KO, *n* = 5). (B) Daily food intake (WT, *n* = 20; KO, *n* = 8). (C) Liver weight (WT, *n* = 6; KO, *n* = 5). (D) HE staining of liver sections. Scale bar, 100 μm. Representative images are shown. (E) qPCR analyses of MASH pathogenic genes in livers (WT, *n* = 10; KO, *n* = 8). (F–L) Control WT and whole-body *Chrna2* KO mice following prolonged GAN diet feeding. (F) Daily food intake (WT, *n* = 13; KO, *n* = 11). (G) Oral glucose tolerance test (WT, *n* = 8; KO, *n* = 6). AUC, area under the curve. (H) Immunoblot analyses for MASH pathogenic signaling proteins in livers of WT and *Chrna2* KO mice following prolonged GAN diet feeding (WT, *n* = 4; KO, *n* = 5). (I) Left, inguinal white adipose tissue (iWAT) weight (WT, *n* = 8; KO, *n* = 6). Right, qPCR analyses of adaptive thermogenic genes in iWAT (WT, *n* = 8; KO, *n* = 6). (J) Left, brown adipose tissue (BAT) weight (WT, *n* = 8; KO, *n* = 6). Right, qPCR analyses of adaptive thermogenic genes in BAT (WT, *n* = 8; KO, *n* = 6). (K) qPCR analyses of non-shivering thermogenic genes in muscles (WT, *n* = 8; KO, *n* = 6). (L) Core body temperature at room temperature (WT, *n* = 8; KO, *n* = 6). The data underlying the graphs in this figure can be found in S8 Data and S4 Raw Images. Mean ± SEM. n.s., not significant. *p < 0.05, **p < 0.01 by an unpaired two-sample Student’s *t* test or Mann–Whitney U test.
(TIF)

S5 Fig Phenotype of liver-specific CHRNA2-deficient mice under basal or MASH condition. Related to Fig 2. (A–G) Control *Chrna2*^fl/fl^ and *Chrna2*^fl/fl^;*AlbCre* mice fed a chow diet. (A) qPCR analyses of *Chrna2* mRNA expression in various tissues (*n* = 4 per group). (B) Body weight (fl/fl, *n* = 5; Cre, *n* = 4). (C) Liver weight (fl/fl, *n* = 5; Cre, *n* = 4). (D) Daily food intake (*n* = 8 per group). (E) HE staining of liver sections. Scale bar, 100 μm. Representative images are shown. (F) qPCR analyses of MASH pathogenic genes in livers (*n* = 8 per group). (G) Left, inguinal white adipose tissue (iWAT) mass (fl/fl, *n* = 10; Cre, *n* = 6). Right, qPCR analyses of adaptive thermogenic genes in iWAT (fl/fl, *n* = 10; Cre, *n* = 6). (H–O) Control *Chrna2*^fl/fl^ and *Chrna2*^fl/fl^;*AlbCre* mice fed a Gubra-Amylin NASH (GAN). (H) Daily food intake (fl/fl, *n* = 10; Cre, *n* = 9). (I) Oral glucose tolerance test (fl/fl, *n* = 12; Cre, *n* = 9). AUC, area under the curve. (J) Plasma insulin levels (*n* = 6 per group). (K) Left, inguinal white adipose tissue (iWAT) mass (*n* = 6 per group). Right, qPCR analyses of adaptive thermogenic genes in iWAT (*n* = 6 per group). (L) Left, brown adipose tissue (BAT) mass (*n* = 6 per group). Right, qPCR analyses of adaptive thermogenic genes in BAT (*n* = 6 per group). (M) qPCR analyses of non-shivering thermogenic genes in muscles (*n* = 6 per group). (N) Core body temperature at room temperature (fl/fl, *n* = 12; Cre, *n* = 9). (O) Plasma triglyceride levels (TG; *n* = 6 per group). The data underlying the graphs in this figure can be found in S9 Data. Mean ± SEM. n.d., not detected; n.s., not significant. *p < 0.05 by an unpaired two-sample Student’s *t* test or Mann–Whitney U test.
(TIF)

S6 Fig Response of ChAT-expressing hepatic immune cells to liver injury. Related to Fig 3. (A–G) Mice were challenged with high-fat diet feeding combined with carbon tetrachloride (HFD+CCl_4_) administration for MASH development or control (chow+vehicle) treatment. (A) qPCR analyses of MASH pathogenic genes in liver tissues of wild-type (WT) mice (Ctrl, *n* = 9; HFD+CCl_4_, *n* = 3). (B) Flow cytometric analyses of the abundance of total cells, Kupffer cells (KCs), and monocyte-derived macrophages (MDMs) that express ChAT in liver non-parenchymal cells (NPCs) from ChAT^BAC^-eGFP mice. Cell numbers were normalized with liver weights (*n* = 4 per group). (C) Flow cytometric analyses of normalized abundance of total KCs and MDMs with liver weights from WT mice (*n* = 4 per group). (D) Flow cytometric analyses of GFP median fluorescence intensity (MFI) for total cells, KCs and MDMs that express ChAT-eGFP in liver NPCs from ChAT^BAC^-eGFP mice (Ctrl, *n* = 4; HFD+CCl_4_, *n* = 4). (E) Flow cytometric analysis of RFP+ CD45+ ChAT-eGFP+ cells in liver NPCs from ChAT-eGPF;*ChATCre-*RFP double reporter mice (GFP marks cells actively expressing ChAT). (F) Daily food intake of WT and *Chrna2* KO mice (WT, *n* = 16; KO, *n* = 13). (G) Daily food intake of control *Chrna2*^fl/fl^ and *Chrna2*^fl/fl^;*AlbCre* mice (*n* = 6 per group). The data underlying the graphs in this figure can be found in S10 Data. Mean ± SEM. *p < 0.05, **p < 0.01, **p < 0.005 by an unpaired two-sample Student’s *t* test or Mann–Whitney U test.
(TIF)

S7 Fig Phenotype of macrophage-specific ChAT-deleted mice under basal or MASH condition. Related to Fig 3. (A) Left, schematic diagram illustrating the generation of ChAT-eGFP;*LysMCre*-RFP mice by crossing ChAT-eGFP, *LysMCre*, and Ai14 animals. Right, flow cytometric analyses of RFP+ macrophages (MΦ) in hepatic ChAT-eGFP^+^ cells of ChAT-eGFP;*LysMCre*-RFP mice. (B–F) *Chat*^fl/fl^ and *Chat*^fl/fl^;*LysMCre* mice at the basal condition. (B) Body weight (fl/fl, *n* = 6; Cre, *n* = 8). (C) Daily food intake (fl/fl, *n* = 6; Cre, *n* = 8). (D) Liver weight (fl/fl, *n* = 6; Cre, *n* = 8). (E) HE of liver sections. Scale bars, 50 μm. Representative images are shown. (F) qPCR analyses of MASH-related molecular markers (fl/fl, *n* = 6; Cre, *n* = 8). (G) Daily food intake and (H) core body temperature of *Chat*^fl/fl^ and *Chat*^fl/fl^;*LysMCre* mice with HFD+CCl4-induced MASH (fl/fl, *n* = 7; Cre, *n* = 6). The data underlying the graphs in this figure can be found in S11 Data. Mean ± SEM. n.s., not significant by an unpaired two-sample Student’s *t* test or Mann–Whitney U test.
(TIF)

S8 Fig Effects of pharmacological inhibition of acetylcholine degradation in chow or hypercaloric diet-fed mice. Related to Fig 4. (A–D) Wild-type (WT) mice fed a high-fat diet (HFD) were treated with vehicle (Veh) or rivastigmine (Riva, 1 mg/kg body weight/day) for 2 weeks. (A) Body weight (Veh, *n* = 10; Riva, *n* = 11). (B) Liver weight (Veh, *n* = 10; Riva, *n* = 11). (C) Liver per body weight ratio (Veh, *n* = 10; Riva, *n* = 11). (D) qPCR analyses of MASH pathogenic genes in the liver (Veh, *n* = 10; Riva, *n* = 11). (E–H) WT mice fed a chow diet were treated with Veh or Riva (1 mg/kg body weight/day) for 2 weeks. (E) Body weight (*n* = 5 per group). (F) Liver weight (*n* = 5 per group). (G) Liver per body weight ratio (*n* = 5 per group). (H) qPCR analyses of MASH pathogenic genes in the liver (Veh, *n* = 8; Riva, *n* = 9). The data underlying the graphs in this figure can be found in S12 Data. Mean ± SEM. n.s., not significant. *p < 0.05, **p < 0.01, ***p < 0.005 by an unpaired two-sample Student’s *t* test or Mann–Whitney U test.
(TIF)

S9 Fig Effects of pharmacological inhibition of acetylcholine degradation in brown adipose tissues of GAN diet-fed mice. Related to Fig 4. Gubra-Amylin NASH (GAN) diet-fed mice were treated with vehicle (Veh) or rivastigmine (Riva, 1 mg/kg body weight/day) for 2 weeks. (A) Control mice. Left, brown adipose tissue (BAT) mass (*n* = 10 per group). Right, qPCR analyses of adaptive thermogenic genes in BAT (*n* = 10 per group). (B) Liver-specific *Chrna2* KO mice. Left, BAT mass (*n* = 9 per group). Right, qPCR analyses of adaptive thermogenic genes in BAT (*n* = 9 per group). The data underlying the graphs in this figure can be found in S13 Data. Mean ± SEM. n.s., not significant by an unpaired two-sample Student’s *t* test or Mann–Whitney U test.
(TIF)

S10 Fig Effects of pharmacological inhibition of acetylcholine degradation in HFD plus CCl4-induced MASH mice. Related to Fig 4. (A) Schematic diagram illustrating the experimental outline. Mice with HFD+CCl_4_-induced MASH were treated with vehicle (Veh) or rivastigmine (Riva, 1 mg/kg body weight/day) for the last 2 weeks on HFD+CCl_4_ diet. (B–D) Control mice. (B) Body weight (Veh, *n* = 17; Riva, *n* = 14). (C) Liver per body weight ratio (Veh, *n* = 17; Riva, *n* = 14). (D) qPCR analyses of MASH pathogenic genes in livers (Veh, *n* = 17; Riva, *n* = 14). (E–G) Liver-specific *Chrna2* KO mice. (E) Body weight (Veh, *n* = 15; Riva, *n* = 13). (F) Liver per body weight ratio (Veh, *n* = 15; Riva, *n* = 13). (G) qPCR analyses of MASH pathogenic genes in livers (Veh, *n* = 15; Riva, *n* = 13). The data underlying the graphs in this figure can be found in S14 Data. Mean ± SEM. n.s., not significant. *p < 0.05, ***p < 0.005 by an unpaired two-sample Student’s *t* test or Mann–Whitney U test.
(TIF)

S1 Table Related to Fig 1. Relative frequencies of ChAT-eGFP^+^ hepatic non-parenchymal cell types.
(DOCX)

S2 Table qPCR primer sequences. (DOCX)

S3 Table Antibodies used in the study. (DOCX)

S1 Data Related to Fig 1. Source data underlying the graphs in Fig 1.
(XLSX)

S2 Data Related to Fig 2. Source data underlying the graphs in Fig 2.
(XLSX)

S3 Data Related to Fig 3. Source data underlying the graphs in Fig 3.
(XLSX)

S4 Data Related to Fig 4. Source data underlying the graphs in Fig 4.
(XLSX)

S5 Data Related to S1 Fig. Source data underlying the graphs in S1 Fig.
(XLSX)

S6 Data Related to S2 Fig. Source data underlying the graphs in S2 Fig.
(XLSX)

S7 Data Related to S3 Fig. Source data underlying the graphs in S3 Fig.
(XLSX)

S8 Data Related to S4 Fig. Source data underlying the graphs in S4 Fig.
(XLSX)

S9 Data Related to S5 Fig. Source data underlying the graphs in S5 Fig.
(XLSX)

S10 Data Related to S6 Fig. Source data underlying the graphs in S6 Fig.
(XLSX)

S11 Data Related to S7 Fig. Source data underlying the graphs in S7 Fig.
(XLSX)

S12 Data Related to S8 Fig. Source data underlying the graphs in S8 Fig.
(XLSX)

S13 Data Related to S9 Fig. Source data underlying the graphs in S9 Fig.
(XLSX)

S14 Data Related to S10 Fig. Source data underlying the graphs in S10 Fig.
(XLSX)

S1 Raw Images Related to Fig 1. Source images underlying the graphs in Fig 1.
(PDF)

S2 Raw Images Related to S1 Fig. Source images underlying the graphs in S1 Fig.
(PDF)

S3 Raw Images Related to S3 Fig. Source images underlying the graphs in S3 Fig.
(PDF)

S4 Raw Images Related to S4 Fig. Source images underlying the graphs in S4 Fig.
(PDF)

## Abbreviations

Acc1: acetyl-Coenzyme A carboxylase alpha
Ach: acetylcholine
Acta2: actin alpha 2, smooth muscle, aorta
AICAR: 5-aminoimidazole-4-carboxamide riboside
ALT: alanine aminotransferase
AMPK: adenosine monophosphate-activated protein kinase
Atf4: activating transcription factor 4
Atgl: patatin-like phospholipase domain containing 2
Atp5b: H+ transporting mitochondrial F1 complex, beta ATP synthase
BAT: brown adipose tissue
BMDM: bone marrow-derived macrophages
BODIPY: 4,4-Difluoro-1,3,5,7,8-Pentamethyl-4-Bora-3a,4a-Diaza-s-Indacene
CaMKK2: Ca2+/calmodulin-dependent protein kinase kinase 2
Ccl2: C-C motif chemokine ligand 2
CCl4: carbon tetrachloride
Cd45: Protein tyrosine phosphatase receptor type C
ChAT: Choline acetyltransferase
CHRNA2: cholinergic receptor nicotinic alpha 2 subunit
Chop: DNA-damage inducible transcript 3
Ck18: Keratin 18
CM: conditioned medium
Cox1: Cytochrome c oxidase subunit I
Cox4: cytochrome c oxidase subunit IV
Cox8b: cytochrome c oxidase subunit 8B
Col1a1: collagen, type I, alpha 1
Cxcl2: chemokine (C-X-C motif) ligand 2
Cxcl10: chemokine (C-X-C motif) ligand 10
DAPI: 6-diamidino-2-phenylindole
DEGs: differentially expressed genes
DMOG: dimethyloxalylglycine
ER: endoplasmic reticulum
Erdj4: DnaJ heat shock protein family (Hsp40) member B9
Fasn: fatty acid synthase
FDA: Food and Drug Administration
FFAs: free fatty acids
FMO: fluorescence-minus-one
GAN: Gubra-Amylin non-alcoholic steatohepatitis (NASH)
Gpat1: glycerol-3-phosphate acyltransferase, mitochondrial
Gsta1: glutathione S-transferase, alpha 1
Gstm3: glutathione S-transferase, mu 3
GTT: glucose tolerance test
HE: hematoxylin-eosin
HFD: high-fat diet
HIF: hypoxia-inducible factor
HIF1α: hypoxia-inducible factor 1 subunit alpha
Hsl: lipase, hormone sensitive
Il1b: interleukin 1 beta
iNOS: nitric oxide synthase 2, inducible
iWAT: inguinal white adipose tissue
JNK: c-Jun NH2-terminal kinase
KCs: Kupffer cells
Kiss1: kisspeptin
KO: knockou
LC-MS/MS: liquid chromatography-tandem mass spectrometry
Lcad: acyl-Coenzyme A dehydrogenase, long-chain
Lgals3: lectin, galactose binding, soluble 3
LPS: lipopolysaccharides
MASH: metabolic dysfunction-associated steatohepatitis
Mcad: acyl-Coenzyme A dehydrogenase, medium chain
MDMs: monocyte-derived macrophages
MFI: median fluorescence intensity
Mgll: monoglyceride lipase
Mmp2: matrix metallopeptidase
Mmp12: matrix metallopeptidase 12
Mmp13: matrix metallopeptidase 13
Mup15: major urinary protein 15
nAChRs: nicotinic acetylcholine receptors
Nd1: NADH dehydrogenase subunit 1
Ndufv1: NADH:ubiquinone oxidoreductase core subunit V1
NF-κB: nuclear factor kappa B
Nic: nicotine
NPCs: non-parenchymal cells
OGTT: oral glucose tolerance test
Ppargc1a: peroxisome proliferative activated receptor, gamma, coactivator 1 alpha
RFP: red fluorescent protein
Scd1: stearoyl-Coenzyme A desaturase 1
Sdha: succinate dehydrogenase complex, subunit A, flavoprotein (Fp)
Sdhb: succinate dehydrogenase complex, subunit B, iron sulfur (Ip)
SL: steatotic liver
Srebp1: sterol regulatory element binding transcription facto
Tfam: Transcription factor A, mitochondrial
Tff3: trefoil factor 3, intestinal
TG: triglyceride
Timp1: tissue inhibitor of metalloproteinase 1
Tnf: tumor necrosis factor
TUNEL: Terminal deoxynucleotidyl transferase dUTP nick end labeling
RNA-seq: RNA sequencing
Ucp1: Uncoupling protein 1
Ucp3: Uncoupling protein 3
Uqcrc2: ubiquinol-cytochrome c reductase core protein 2
Vegfa: vascular endothelial growth factor A
WT: wild-type
Xbp1s: X-box binding protein 1, spliced form
