# Supplementary figures and images for: Signaling through the nicotinic acetylcholine receptor in the liver protects against the development of metabolic dysfunction-associated steatohepatitis
Source: PLoS Biol. 2024 Jul 19;22(7):e3002728. doi: 10.1371/journal.pbio.3002728 (PMC11290650; doi:10.1371/journal.pbio.3002728)

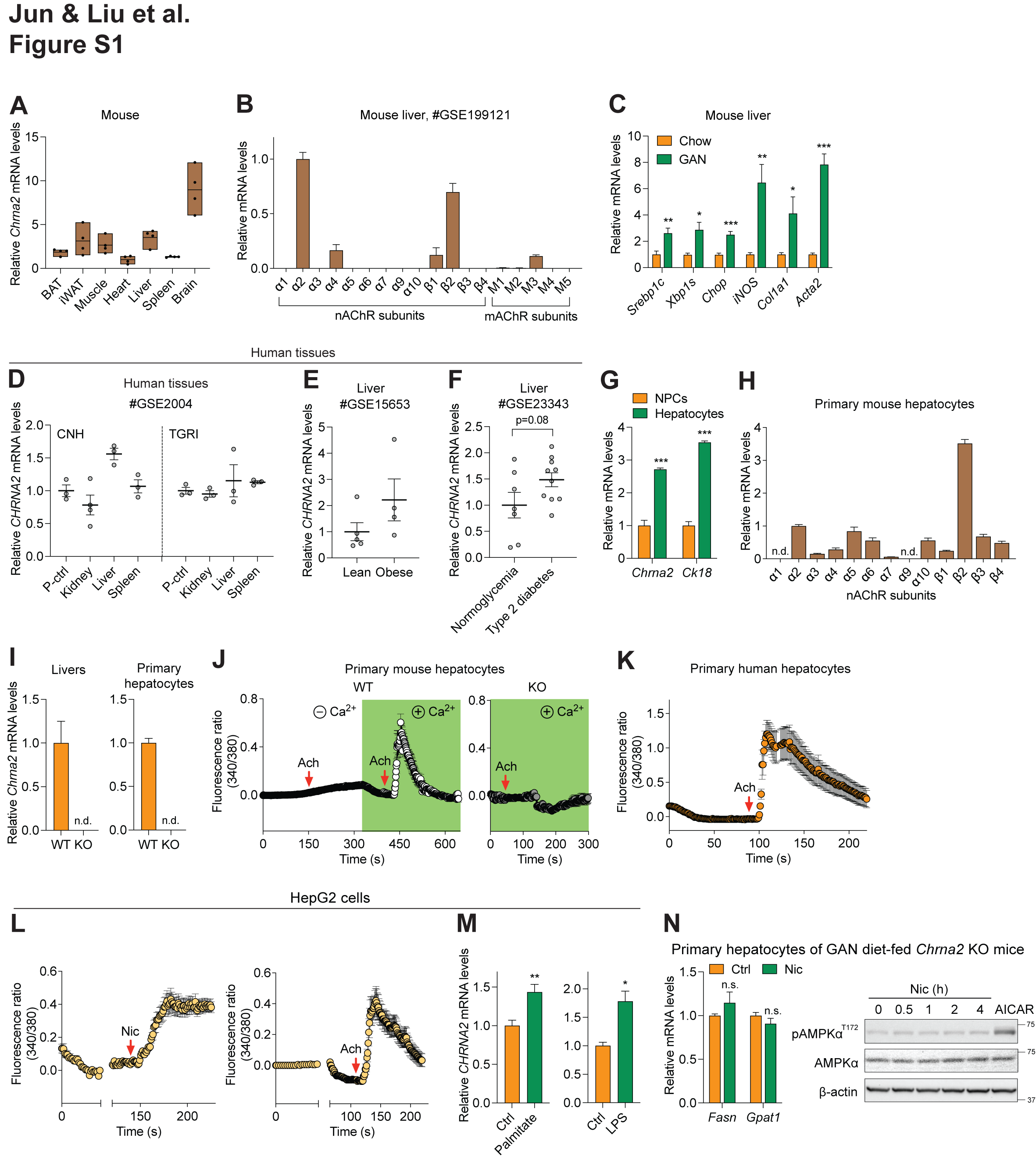

Supplement: S1 Fig — (A) qPCR analyses of Chrna2 expression in various murine tissues (n = 4 per group). (B) Expression levels of nicotinic and muscarinic acetylcholine receptor subunits (nAChRs and mAChRs) in mouse livers from transcriptome data from NCBI Gene Expression Omnibus dataset GSE199121 (n = 3, normal control group from this study). Chrna8 is not listed in the figures since it has only been detected in chicken. (C) qPCR analyses of hepatic MASH pathogenic genes in wild-type (WT) mice fed a chronic GAN or chow diet (n = 5 per group). (D–F) Human tissue transcriptome data of CHRNA2 from the NCBI Gene Expression Omnibus database. (D) GSE2004 dataset [CNH (Children’s National Medical Center); positive control (P-ctrl, universal human reference RNA), n = 3; kidney, n = 4; liver, n = 3; spleen, n = 3; TGRI (Translational Genomics Research Institute), n = 3]. (E) GSE15653 dataset (livers; healthy lean subjects, n = 5; obese subjects, n = 4). (F) GSE23343 dataset (livers; normoglycemic participants, n = 7; type 2 patients with diabetes, n = 10). (G) qPCR analyses of Chrna2 and hepatocyte marker Ck18 in primary liver non-parenchymal cells (NPCs) and hepatocytes from WT mice (NPCs, n = 5; hepatocytes, n = 6). (H) qPCR analyses of genes encoding nAChR subunits in mouse primary hepatocytes (n = 6). (I) qPCR analyses of Chrna2 expression in liver tissues and primary hepatocytes from control WT and whole-body Chrna2 knockout (KO) mice (liver, n = 4; hepatocytes, n = 6). (J) Intracellular calcium uptake mediated by CHRNA2 agonist acetylcholine (Ach, 100 μm) in the presence or absence of calcium in primary hepatocytes from control WT and Chrna2 KO mice (WT, n = 12; KO, n = 14). (K) Intracellular calcium uptake mediated by 100 μm Ach in primary human hepatocytes (n = 23). (L) Intracellular calcium levels in human hepatoma cell line HepG2 with 500 μm nicotine (Nic) (n = 13) or 100 μm Ach (n = 15) stimulation. (M) qPCR analyses of CHRNA2 expression in HepG2 cells treated with vehicle (Ctrl), pa [file pbio.3002728.s001.tif]

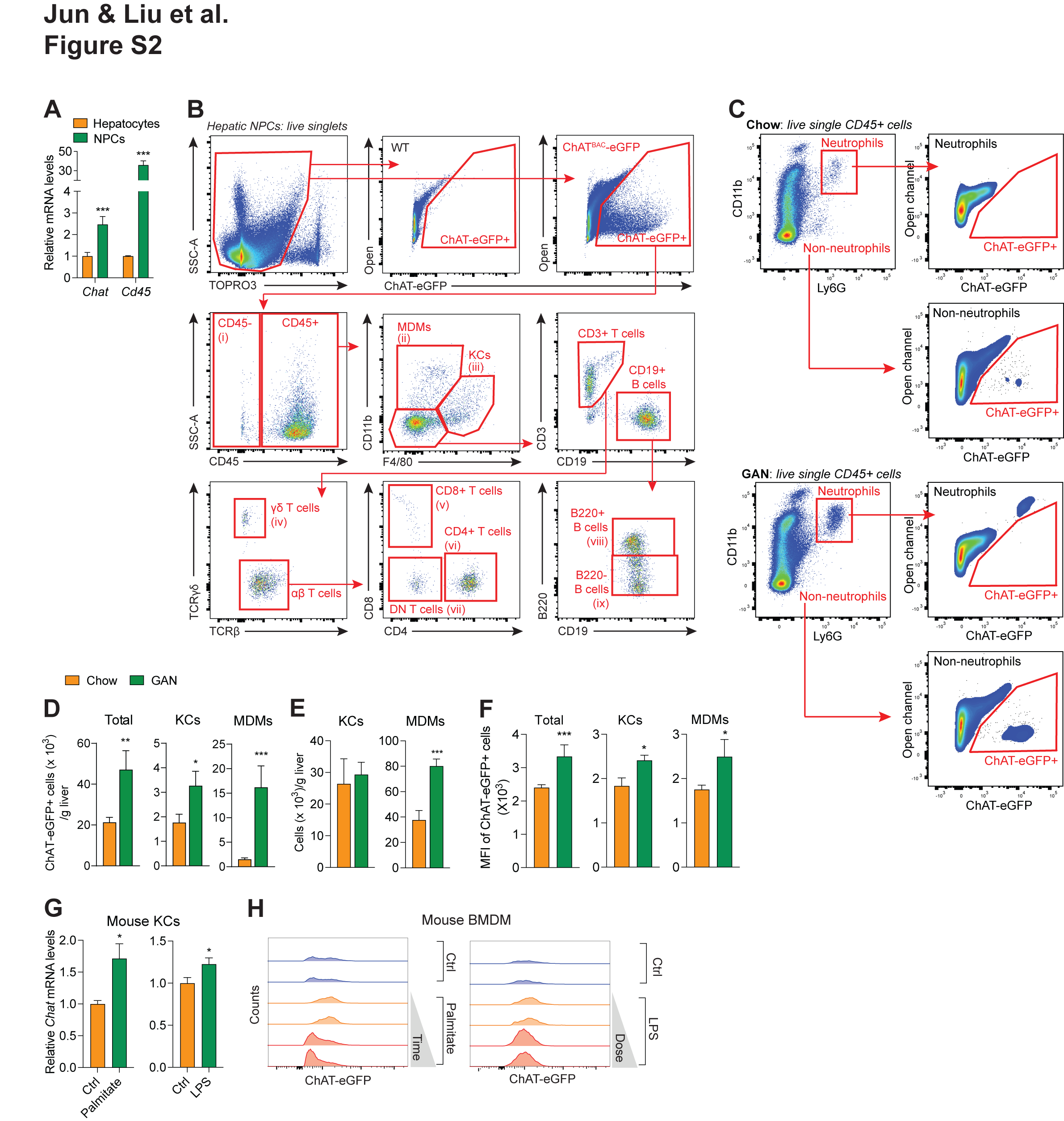

Supplement: S2 Fig — (A) qPCR analyses of Chat and immune marker Cd45 in primary hepatocytes and non-parenchymal cells (NPCs) from wild-type (WT) mice (NPCs, n = 5; hepatocytes, n = 6). (B) Immune profiles of ChAT-expressing cells in mouse hepatic NPCs using flow cytometric analysis. Gating strategy for surface marker-based immunophenotyping of ChAT-eGFP+ NPCs. Live singlets (TOPRO3-) were gated on for ChAT-eGFP+ cells. ChAT-eGFP+ cells were defined as CD45+ or CD45- (i), and CD45+ cells gated on for monocyte-derived macrophages (MDMs; ii, CD11bhi F4/80lo) and Kupffer cells (KCs; iii, CD11blo F4/80hi). Non-myeloid cells (CD11b- F4/80-) were defined broadly as CD3+ T cells or CD19+ B cells. CD3+ T cells were then defined as TCRγδ+ (γδ T cells, iv) or TCRβ+ (αβ T cells), which were further broken down into CD8+ (v), CD4+ (vi), or double negative (DN) αβ T cells (vii). CD19+ B cells were defined as B220+(viii) or B220- (ix). The frequencies of each subpopulation are listed in S1 Table. (C) Representative gating strategy for identification of neutrophils (CD45+ CD11b+ Ly6G+) and non-neutrophils in livers from chow diet-fed and Gubra-Amylin NASH (GAN) diet-fed mice. No significant presence of ChAT-eGFP+ neutrophils was detected in the liver. (D) Flow cytometric analyses of the abundance of total cells, KCs and MDMs that express ChAT in liver NPCs from ChATBAC-eGFP mice fed with chow diet or GAN diet. Cell numbers were normalized with liver weights (chow, n = 10; GAN, n = 6). (E) Flow cytometric analyses of normalized abundance of total KCs and MDMs with liver weights from WT mice fed with chow diet or GAN diet (chow, n = 8; GAN, n = 10). (F) ChAT-eGFP median fluorescence intensity (MFI) for ChAT-eGFP+ cells (chow, n = 10; GAN, n = 6). (G) qPCR analyses of Chat in primary WT mouse KCs treated with vehicle, 0.5 mM palmitate for 18 h (left; n = 6 per group) or 10 ng/ml LPS for 4 h (right; Ctrl, n = 8; LPS, n = 10). (H) Flow cytometry histograms of ChAT-eGFP+ bone marrow-derived macrophages (BMD [file pbio.3002728.s002.tif]

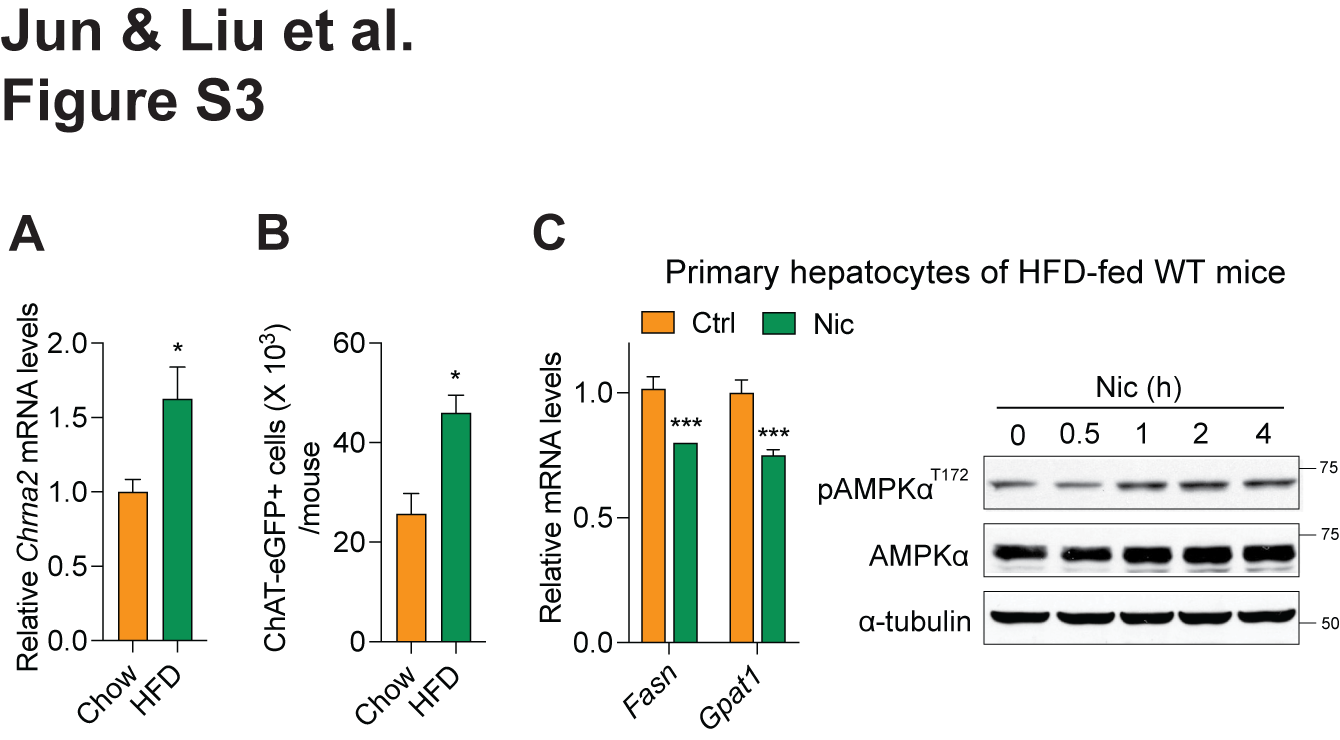

Supplement: S3 Fig — (A) qPCR analyses of hepatic Chrna2 expression in wild-type (WT) mice fed with a control chow diet or a high-fat diet (HFD, 11 weeks; chow, n = 9; n = 8, HFD). (B) Flow cytometric analyses for the abundance of total non-parenchymal cells that express ChAT in livers from ChATBAC-eGFP mice fed chow diet or HFD for 11 weeks (chow; n = 5; HFD, n = 3). (C) Primary mouse hepatocytes with elevated metabolic stress signaling related to MASH were isolated from WT mice fed with HFD for 18 weeks. Left, qPCR analyses of lipogenic genes in vehicle (Ctrl) or Nic (2 mM for 6 h)-treated hepatocytes (n = 6 per group). Right, immunoblot analyses for phosphorylated and total AMPK in hepatocytes exposed to 2 mM Nic for the time indicated. The data underlying the graphs in this figure can be found in S7 Data and S3 Raw Images. Mean ± SEM. *p < 0.05, ***p < 0.005 by an unpaired two-sample Student’s t test. (TIF) [file pbio.3002728.s003.tif]

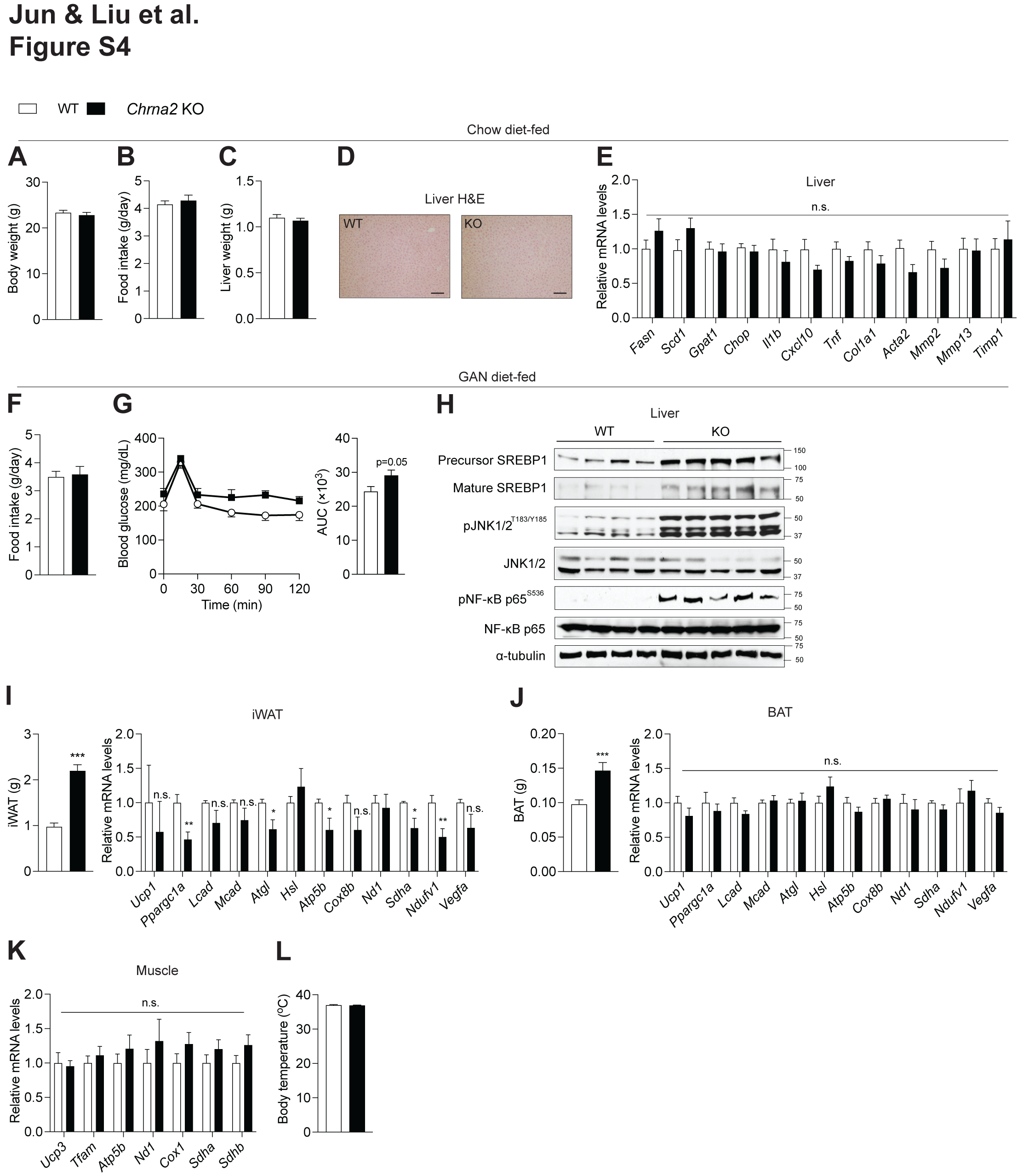

Supplement: S4 Fig — (A–E) Control wild-type (WT) and whole-body Chrna2 KO mice fed a chow diet. (A) Body weight (WT, n = 6; KO, n = 5). (B) Daily food intake (WT, n = 20; KO, n = 8). (C) Liver weight (WT, n = 6; KO, n = 5). (D) HE staining of liver sections. Scale bar, 100 μm. Representative images are shown. (E) qPCR analyses of MASH pathogenic genes in livers (WT, n = 10; KO, n = 8). (F–L) Control WT and whole-body Chrna2 KO mice following prolonged GAN diet feeding. (F) Daily food intake (WT, n = 13; KO, n = 11). (G) Oral glucose tolerance test (WT, n = 8; KO, n = 6). AUC, area under the curve. (H) Immunoblot analyses for MASH pathogenic signaling proteins in livers of WT and Chrna2 KO mice following prolonged GAN diet feeding (WT, n = 4; KO, n = 5). (I) Left, inguinal white adipose tissue (iWAT) weight (WT, n = 8; KO, n = 6). Right, qPCR analyses of adaptive thermogenic genes in iWAT (WT, n = 8; KO, n = 6). (J) Left, brown adipose tissue (BAT) weight (WT, n = 8; KO, n = 6). Right, qPCR analyses of adaptive thermogenic genes in BAT (WT, n = 8; KO, n = 6). (K) qPCR analyses of non-shivering thermogenic genes in muscles (WT, n = 8; KO, n = 6). (L) Core body temperature at room temperature (WT, n = 8; KO, n = 6). The data underlying the graphs in this figure can be found in S8 Data and S4 Raw Images. Mean ± SEM. n.s., not significant. *p < 0.05, **p < 0.01 by an unpaired two-sample Student’s t test or Mann–Whitney U test. (TIF) [file pbio.3002728.s004.tif]

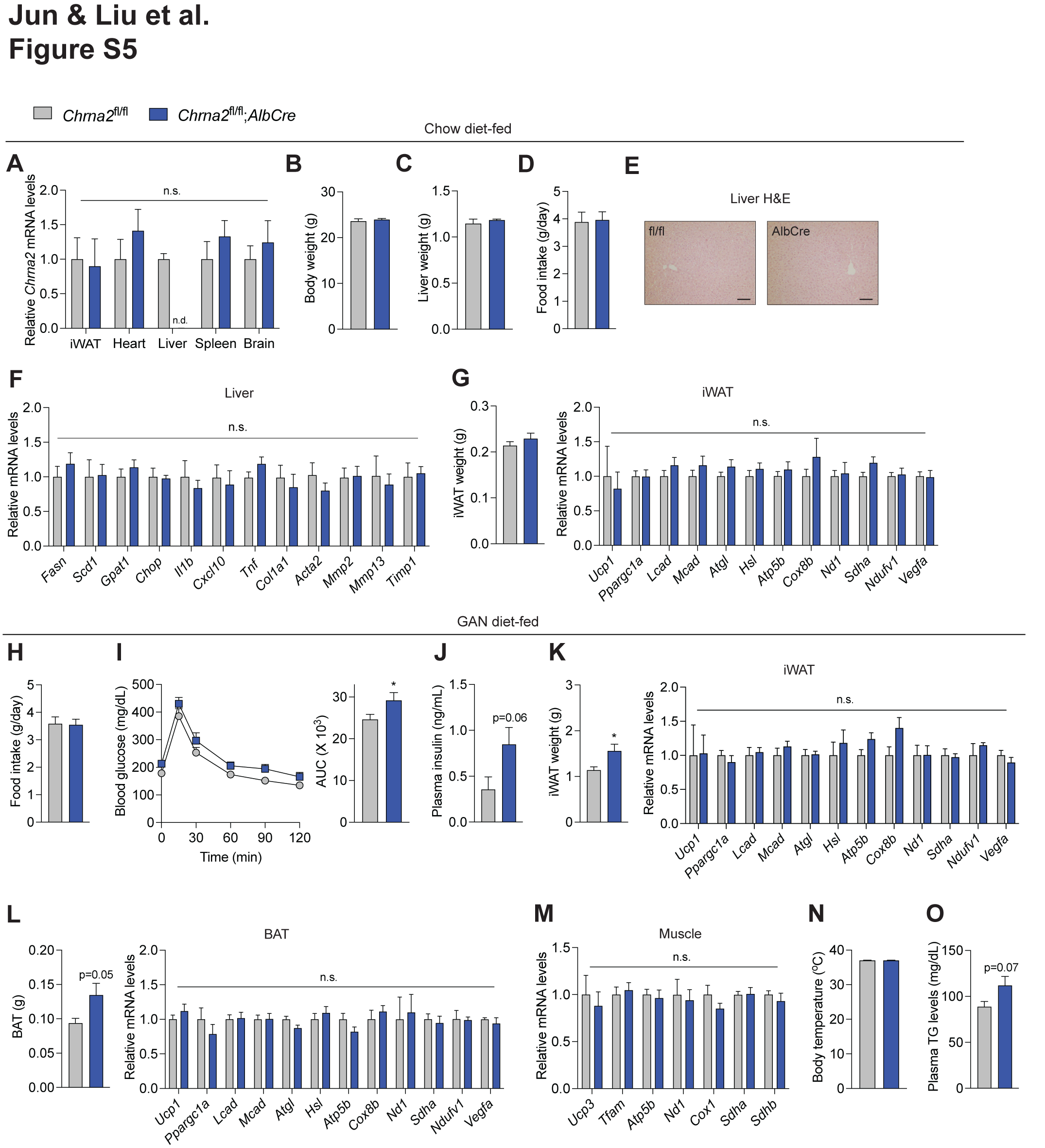

Supplement: S5 Fig — (A–G) Control Chrna2fl/fl and Chrna2fl/fl;AlbCre mice fed a chow diet. (A) qPCR analyses of Chrna2 mRNA expression in various tissues (n = 4 per group). (B) Body weight (fl/fl, n = 5; Cre, n = 4). (C) Liver weight (fl/fl, n = 5; Cre, n = 4). (D) Daily food intake (n = 8 per group). (E) HE staining of liver sections. Scale bar, 100 μm. Representative images are shown. (F) qPCR analyses of MASH pathogenic genes in livers (n = 8 per group). (G) Left, inguinal white adipose tissue (iWAT) mass (fl/fl, n = 10; Cre, n = 6). Right, qPCR analyses of adaptive thermogenic genes in iWAT (fl/fl, n = 10; Cre, n = 6). (H–O) Control Chrna2fl/fl and Chrna2fl/fl;AlbCre mice fed a Gubra-Amylin NASH (GAN). (H) Daily food intake (fl/fl, n = 10; Cre, n = 9). (I) Oral glucose tolerance test (fl/fl, n = 12; Cre, n = 9). AUC, area under the curve. (J) Plasma insulin levels (n = 6 per group). (K) Left, inguinal white adipose tissue (iWAT) mass (n = 6 per group). Right, qPCR analyses of adaptive thermogenic genes in iWAT (n = 6 per group). (L) Left, brown adipose tissue (BAT) mass (n = 6 per group). Right, qPCR analyses of adaptive thermogenic genes in BAT (n = 6 per group). (M) qPCR analyses of non-shivering thermogenic genes in muscles (n = 6 per group). (N) Core body temperature at room temperature (fl/fl, n = 12; Cre, n = 9). (O) Plasma triglyceride levels (TG; n = 6 per group). The data underlying the graphs in this figure can be found in S9 Data. Mean ± SEM. n.d., not detected; n.s., not significant. *p < 0.05 by an unpaired two-sample Student’s t test or Mann–Whitney U test. (TIF) [file pbio.3002728.s005.tif]

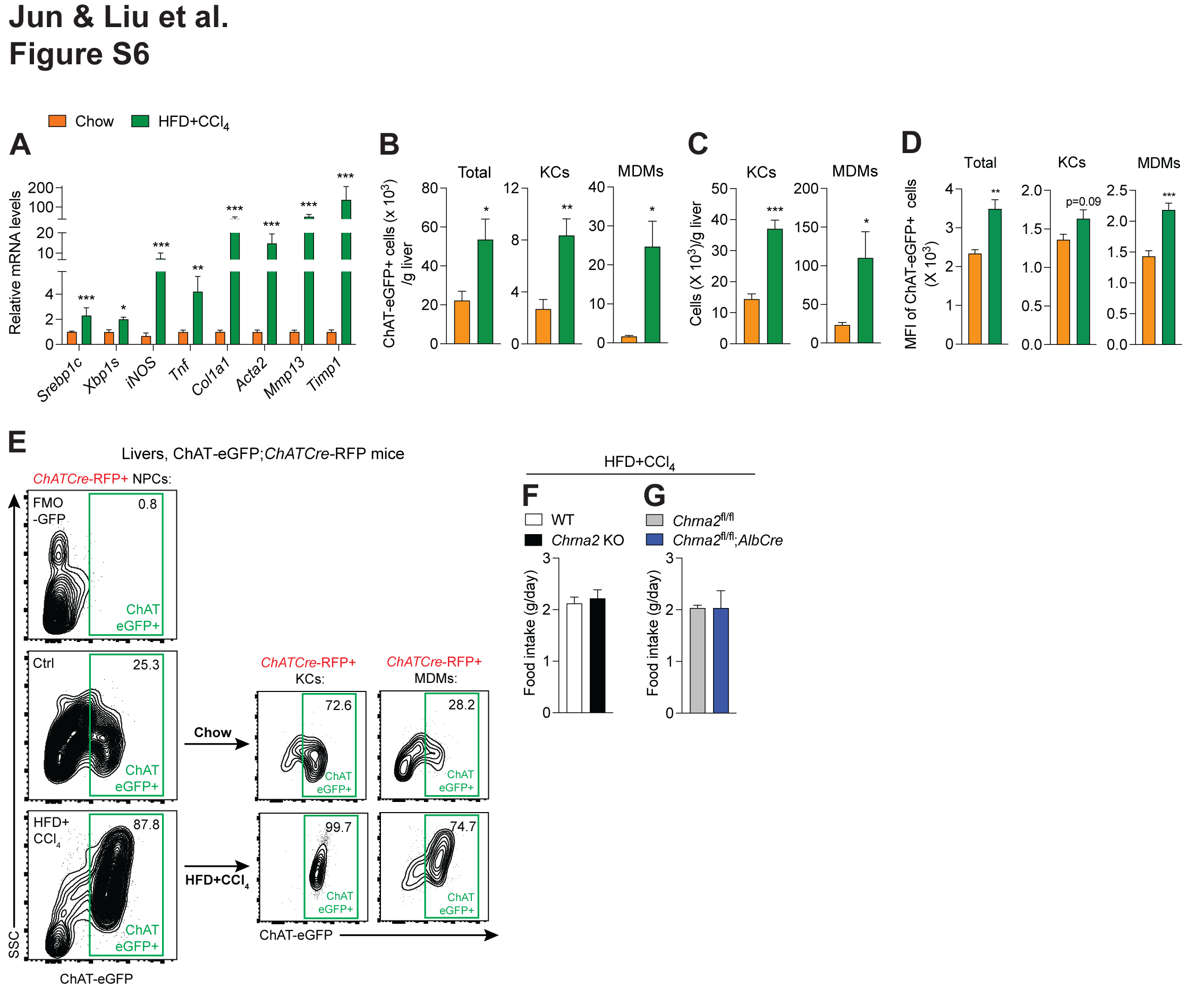

Supplement: S6 Fig — (A–G) Mice were challenged with high-fat diet feeding combined with carbon tetrachloride (HFD+CCl4) administration for MASH development or control (chow+vehicle) treatment. (A) qPCR analyses of MASH pathogenic genes in liver tissues of wild-type (WT) mice (Ctrl, n = 9; HFD+CCl4, n = 3). (B) Flow cytometric analyses of the abundance of total cells, Kupffer cells (KCs), and monocyte-derived macrophages (MDMs) that express ChAT in liver non-parenchymal cells (NPCs) from ChATBAC-eGFP mice. Cell numbers were normalized with liver weights (n = 4 per group). (C) Flow cytometric analyses of normalized abundance of total KCs and MDMs with liver weights from WT mice (n = 4 per group). (D) Flow cytometric analyses of GFP median fluorescence intensity (MFI) for total cells, KCs and MDMs that express ChAT-eGFP in liver NPCs from ChATBAC-eGFP mice (Ctrl, n = 4; HFD+CCl4, n = 4). (E) Flow cytometric analysis of RFP+ CD45+ ChAT-eGFP+ cells in liver NPCs from ChAT-eGPF;ChATCre-RFP double reporter mice (GFP marks cells actively expressing ChAT). (F) Daily food intake of WT and Chrna2 KO mice (WT, n = 16; KO, n = 13). (G) Daily food intake of control Chrna2fl/fl and Chrna2fl/fl;AlbCre mice (n = 6 per group). The data underlying the graphs in this figure can be found in S10 Data. Mean ± SEM. *p < 0.05, **p < 0.01, **p < 0.005 by an unpaired two-sample Student’s t test or Mann–Whitney U test. (TIF) [file pbio.3002728.s006.tif]

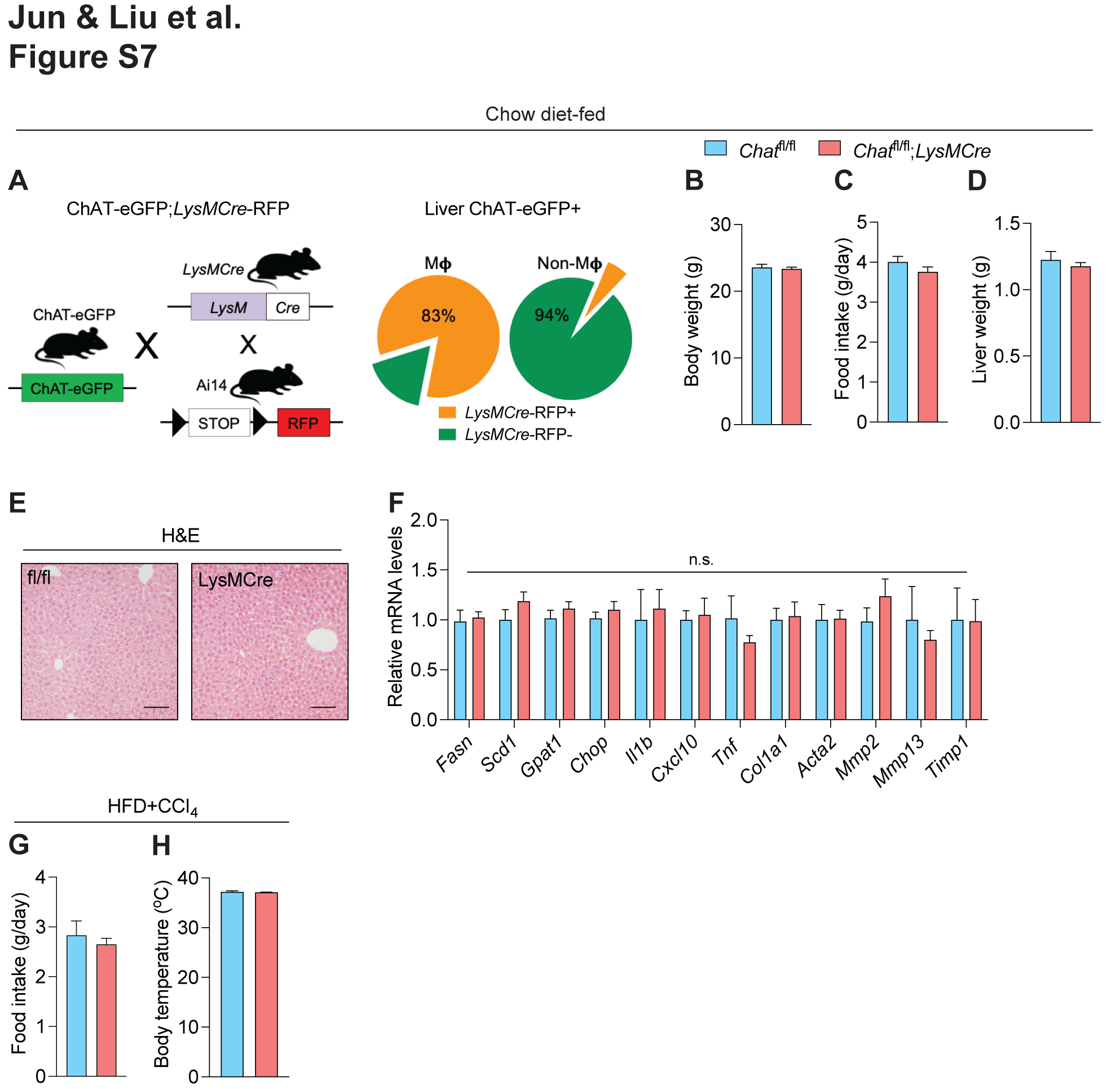

Supplement: S7 Fig — (A) Left, schematic diagram illustrating the generation of ChAT-eGFP;LysMCre-RFP mice by crossing ChAT-eGFP, LysMCre, and Ai14 animals. Right, flow cytometric analyses of RFP+ macrophages (MΦ) in hepatic ChAT-eGFP+ cells of ChAT-eGFP;LysMCre-RFP mice. (B–F) Chatfl/fl and Chatfl/fl;LysMCre mice at the basal condition. (B) Body weight (fl/fl, n = 6; Cre, n = 8). (C) Daily food intake (fl/fl, n = 6; Cre, n = 8). (D) Liver weight (fl/fl, n = 6; Cre, n = 8). (E) HE of liver sections. Scale bars, 50 μm. Representative images are shown. (F) qPCR analyses of MASH-related molecular markers (fl/fl, n = 6; Cre, n = 8). (G) Daily food intake and (H) core body temperature of Chatfl/fl and Chatfl/fl;LysMCre mice with HFD+CCl4-induced MASH (fl/fl, n = 7; Cre, n = 6). The data underlying the graphs in this figure can be found in S11 Data. Mean ± SEM. n.s., not significant by an unpaired two-sample Student’s t test or Mann–Whitney U test. (TIF) [file pbio.3002728.s007.tif]

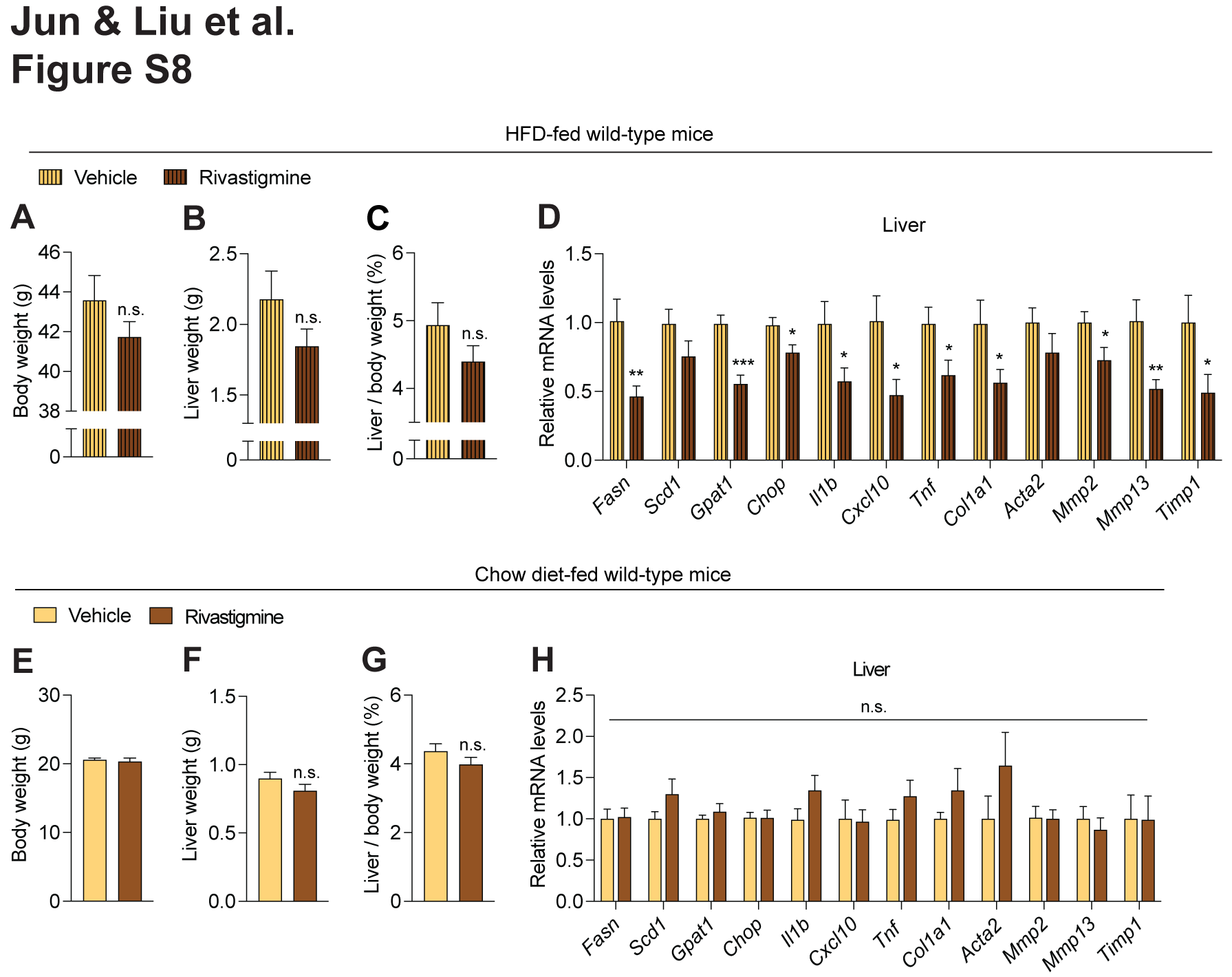

Supplement: S8 Fig — (A–D) Wild-type (WT) mice fed a high-fat diet (HFD) were treated with vehicle (Veh) or rivastigmine (Riva, 1 mg/kg body weight/day) for 2 weeks. (A) Body weight (Veh, n = 10; Riva, n = 11). (B) Liver weight (Veh, n = 10; Riva, n = 11). (C) Liver per body weight ratio (Veh, n = 10; Riva, n = 11). (D) qPCR analyses of MASH pathogenic genes in the liver (Veh, n = 10; Riva, n = 11). (E–H) WT mice fed a chow diet were treated with Veh or Riva (1 mg/kg body weight/day) for 2 weeks. (E) Body weight (n = 5 per group). (F) Liver weight (n = 5 per group). (G) Liver per body weight ratio (n = 5 per group). (H) qPCR analyses of MASH pathogenic genes in the liver (Veh, n = 8; Riva, n = 9). The data underlying the graphs in this figure can be found in S12 Data. Mean ± SEM. n.s., not significant. *p < 0.05, **p < 0.01, ***p < 0.005 by an unpaired two-sample Student’s t test or Mann–Whitney U test. (TIF) [file pbio.3002728.s008.tif]

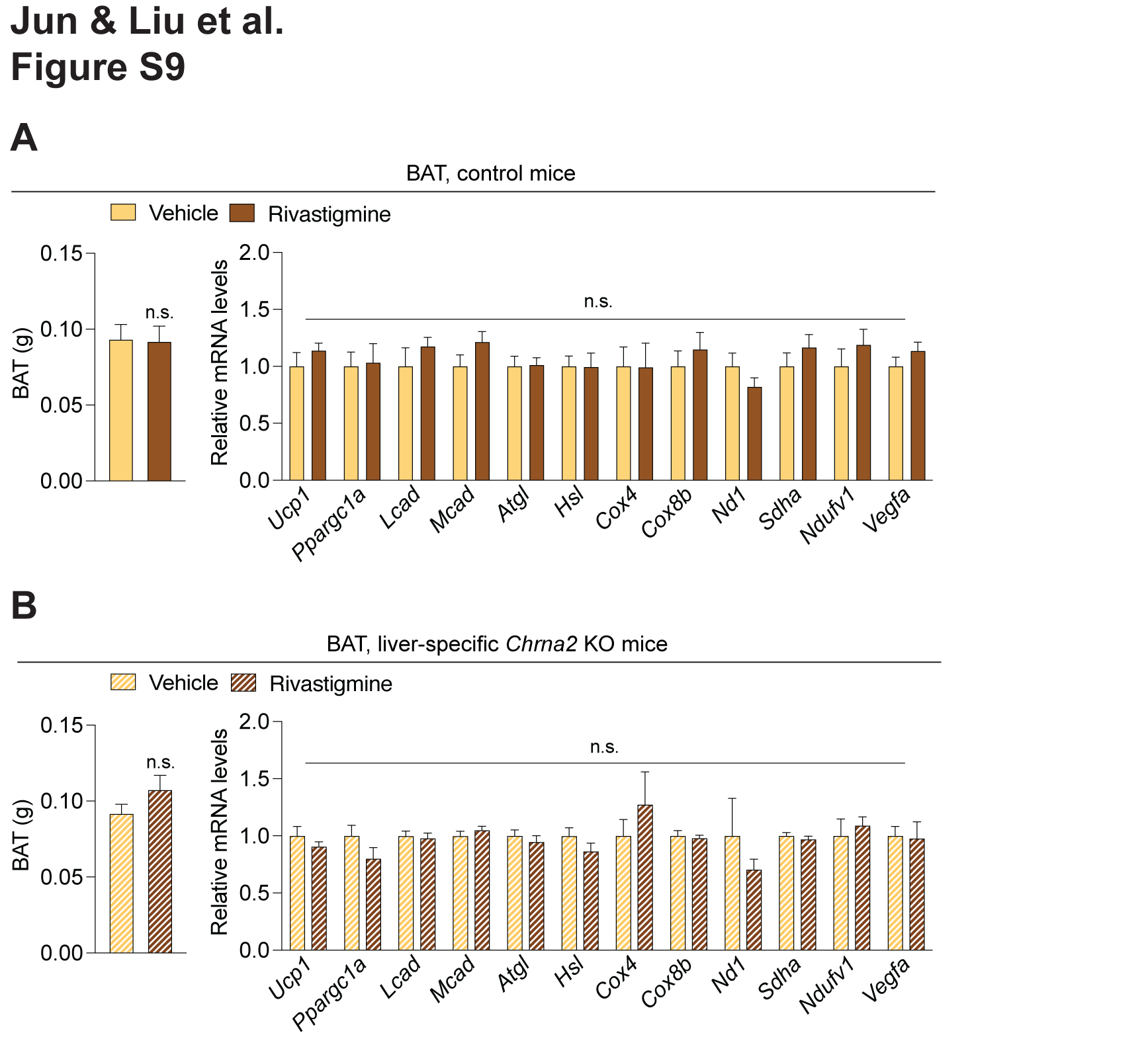

Supplement: S9 Fig — Gubra-Amylin NASH (GAN) diet-fed mice were treated with vehicle (Veh) or rivastigmine (Riva, 1 mg/kg body weight/day) for 2 weeks. (A) Control mice. Left, brown adipose tissue (BAT) mass (n = 10 per group). Right, qPCR analyses of adaptive thermogenic genes in BAT (n = 10 per group). (B) Liver-specific Chrna2 KO mice. Left, BAT mass (n = 9 per group). Right, qPCR analyses of adaptive thermogenic genes in BAT (n = 9 per group). The data underlying the graphs in this figure can be found in S13 Data. Mean ± SEM. n.s., not significant by an unpaired two-sample Student’s t test or Mann–Whitney U test. (TIF) [file pbio.3002728.s009.tif]

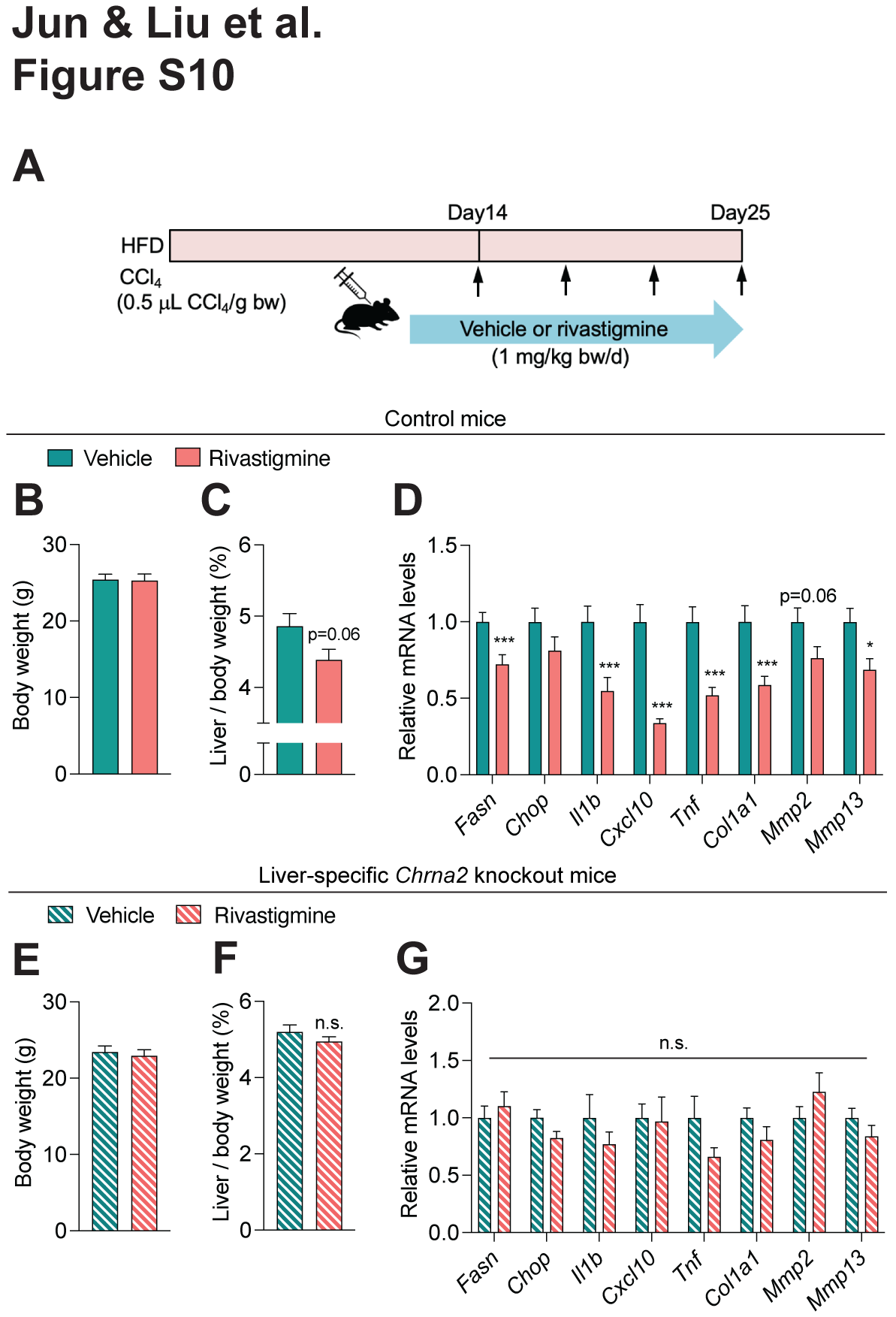

Supplement: S10 Fig — (A) Schematic diagram illustrating the experimental outline. Mice with HFD+CCl4-induced MASH were treated with vehicle (Veh) or rivastigmine (Riva, 1 mg/kg body weight/day) for the last 2 weeks on HFD+CCl4 diet. (B–D) Control mice. (B) Body weight (Veh, n = 17; Riva, n = 14). (C) Liver per body weight ratio (Veh, n = 17; Riva, n = 14). (D) qPCR analyses of MASH pathogenic genes in livers (Veh, n = 17; Riva, n = 14). (E–G) Liver-specific Chrna2 KO mice. (E) Body weight (Veh, n = 15; Riva, n = 13). (F) Liver per body weight ratio (Veh, n = 15; Riva, n = 13). (G) qPCR analyses of MASH pathogenic genes in livers (Veh, n = 15; Riva, n = 13). The data underlying the graphs in this figure can be found in S14 Data. Mean ± SEM. n.s., not significant. *p < 0.05, ***p < 0.005 by an unpaired two-sample Student’s t test or Mann–Whitney U test. (TIF) [file pbio.3002728.s010.tif]

Fig. 1D

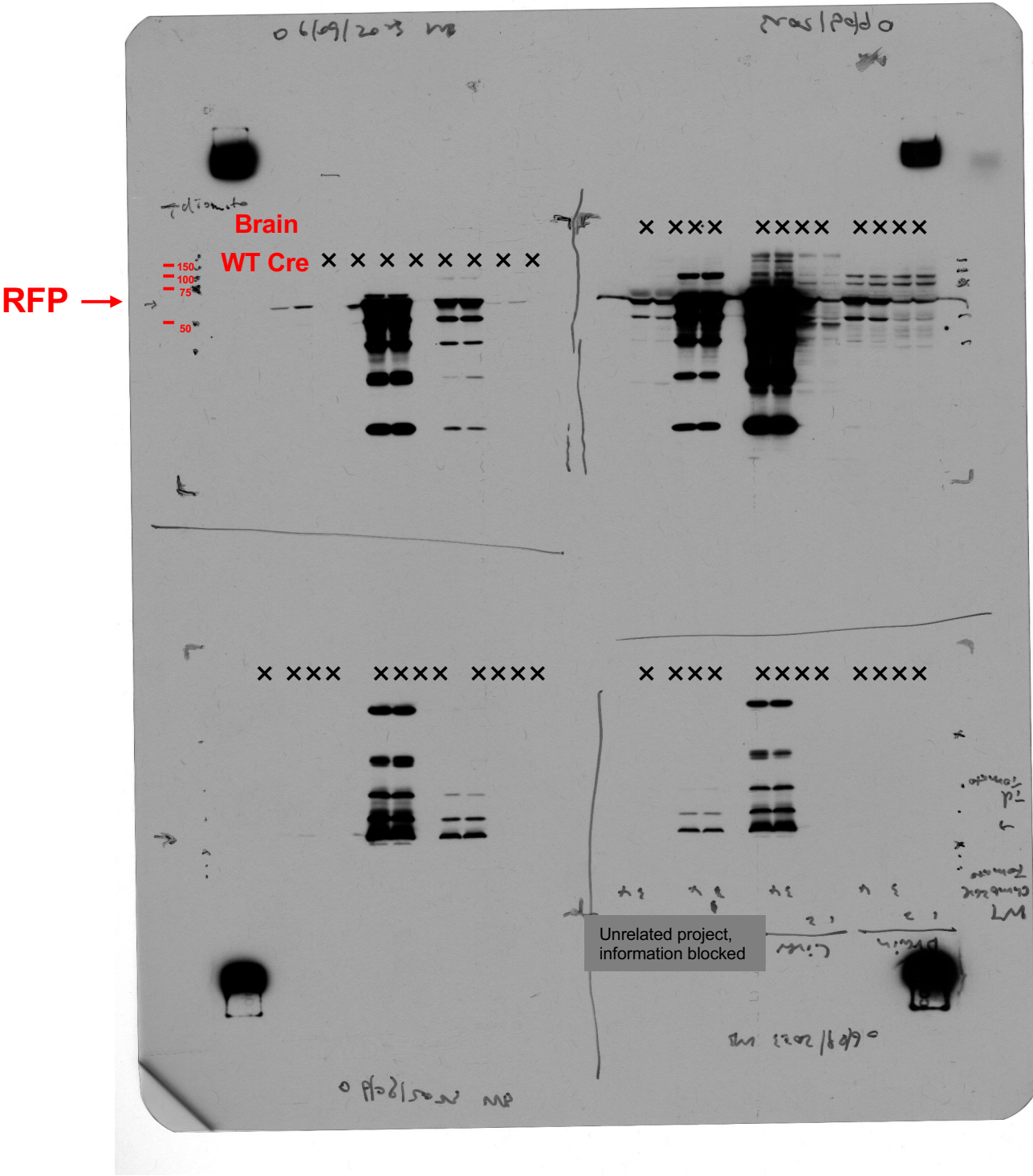

**RFP →**

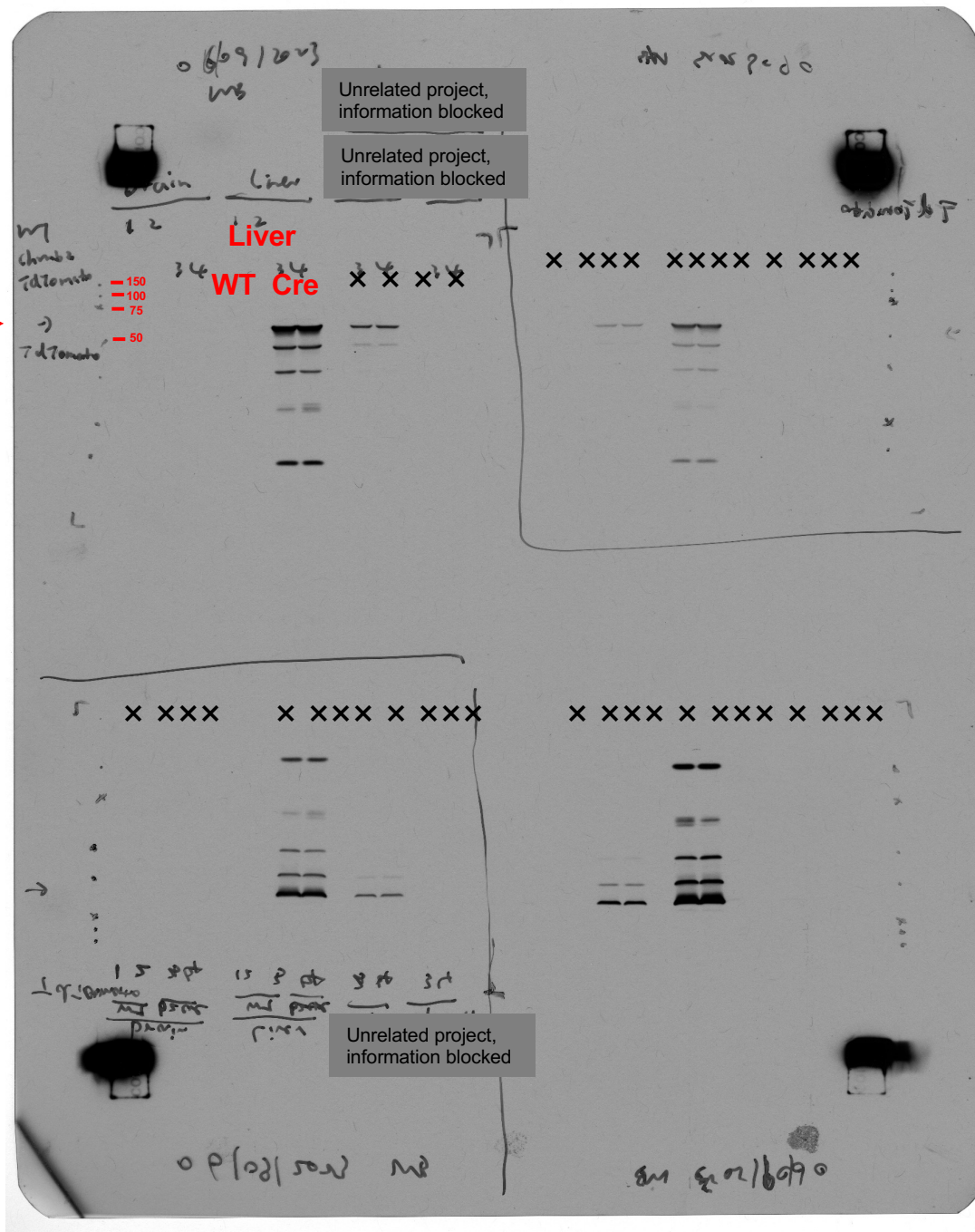

Fig. 1D

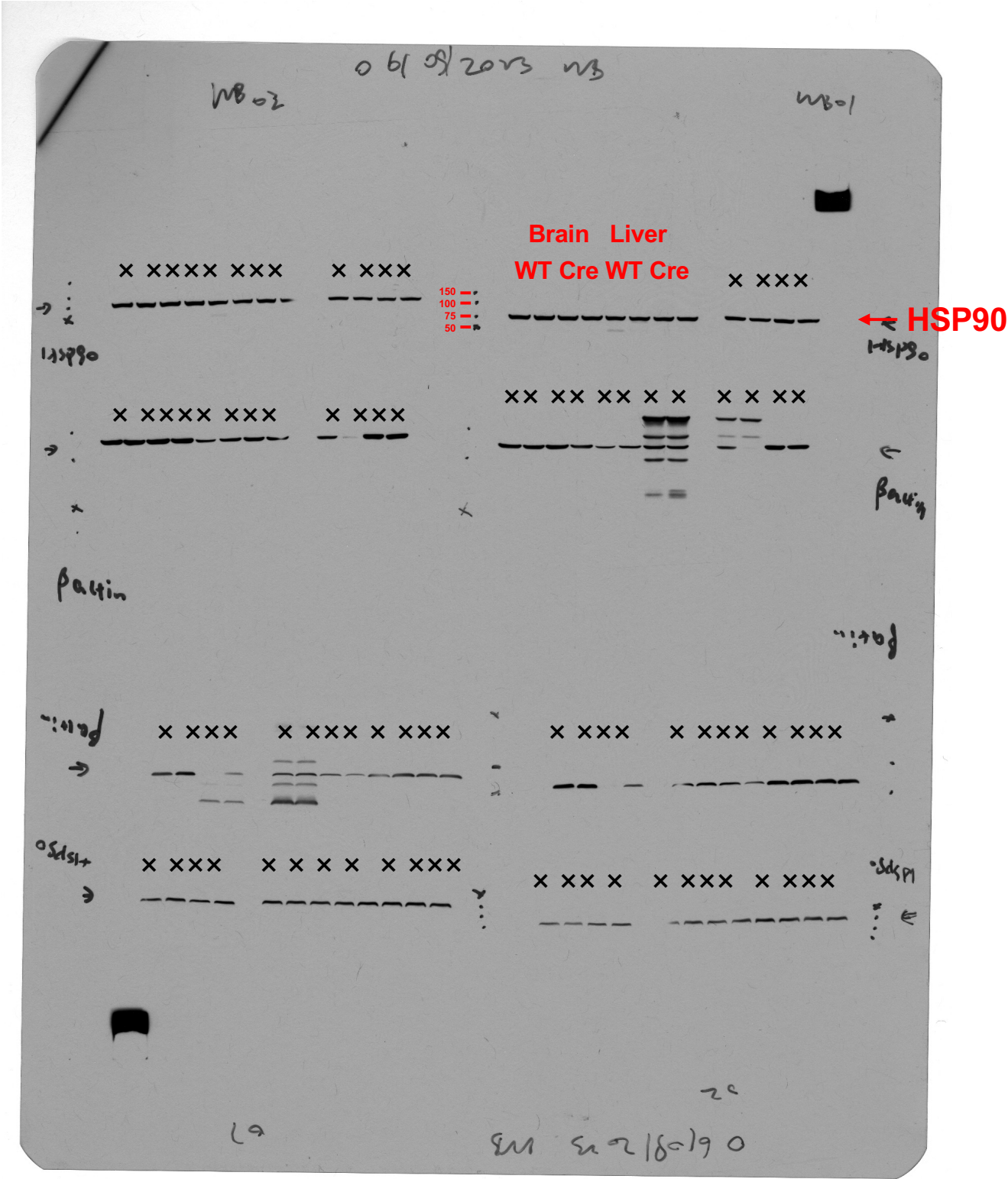

Fig. 1L

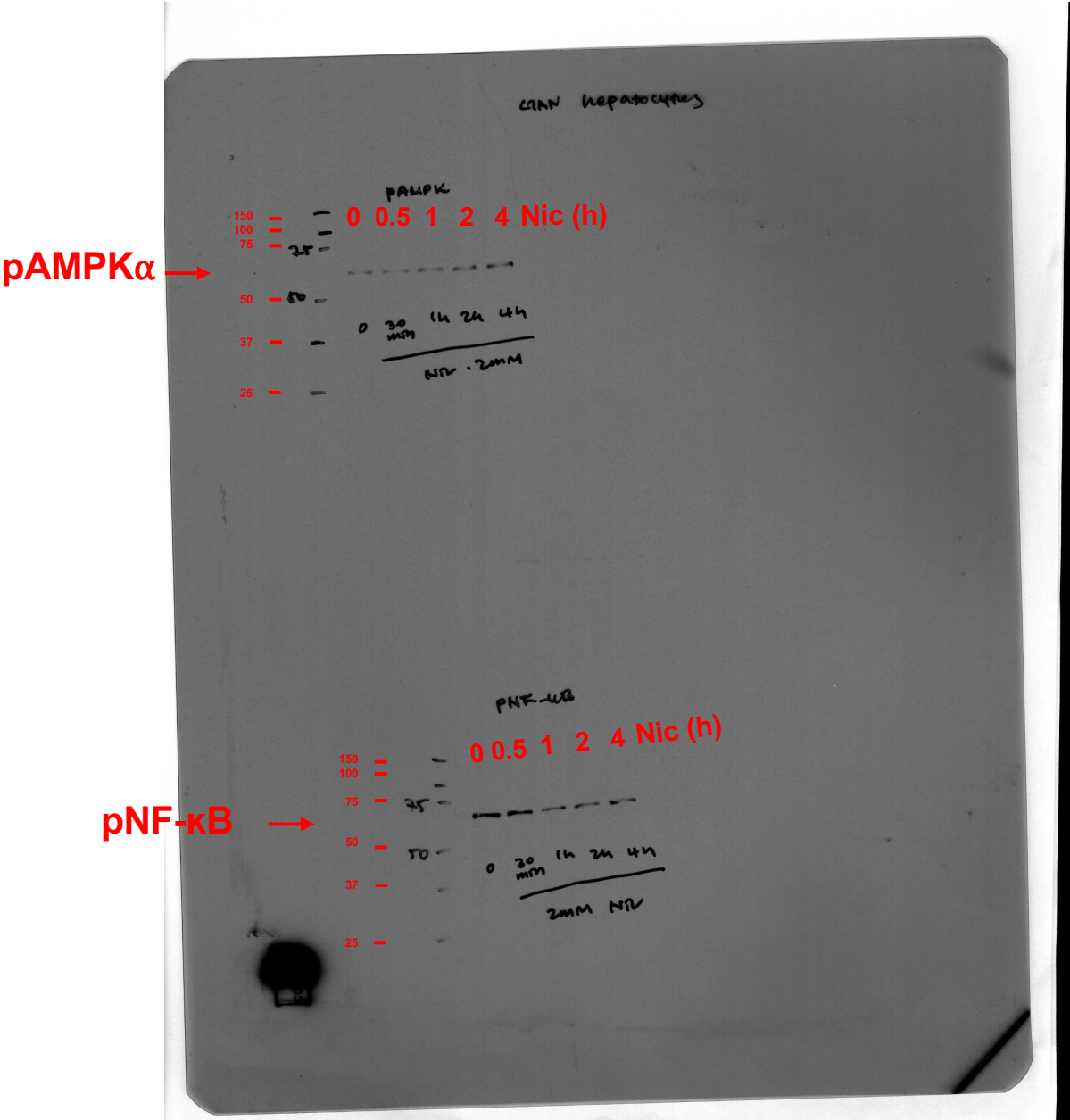

Fig. 1L

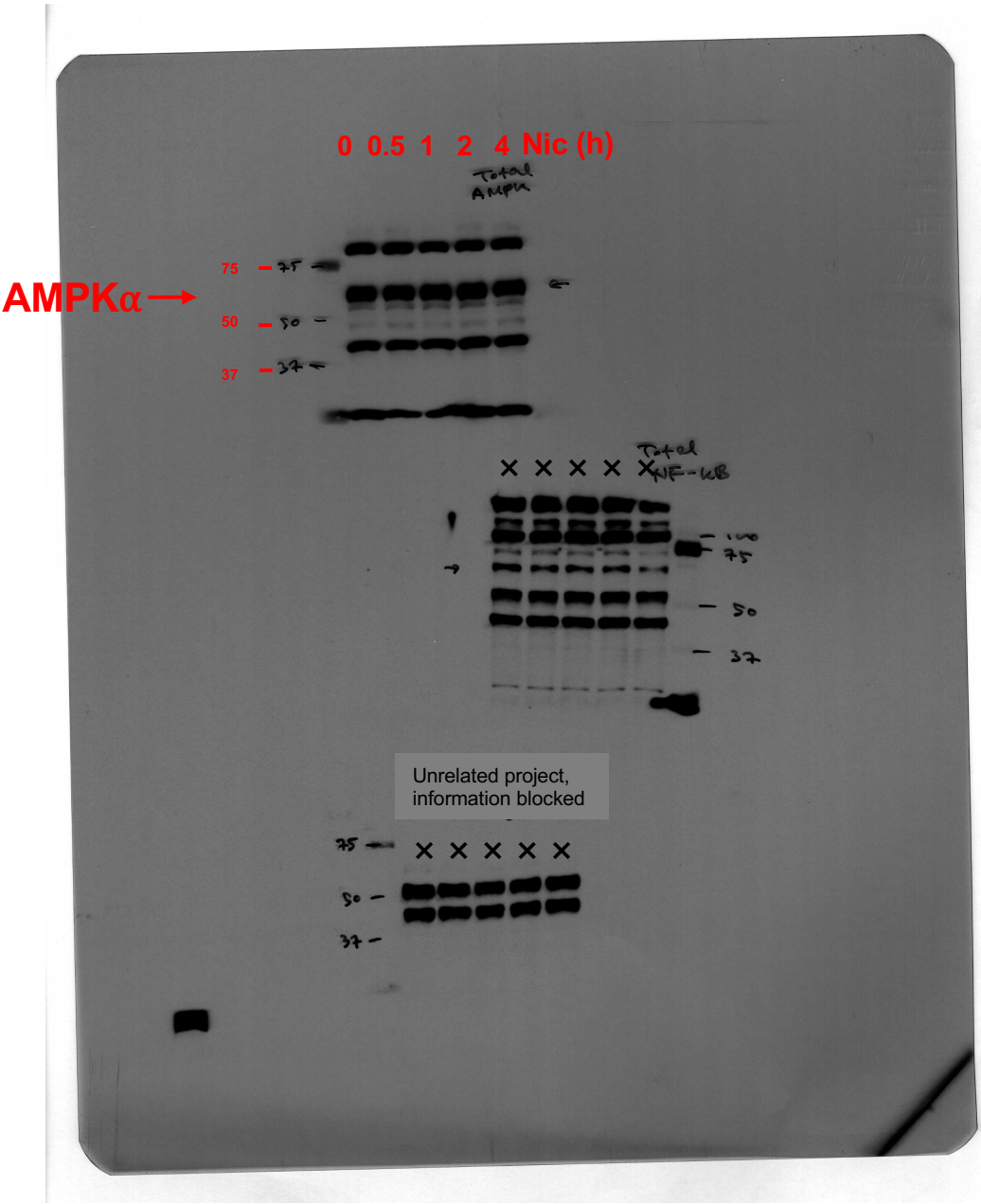

Fig. 1L

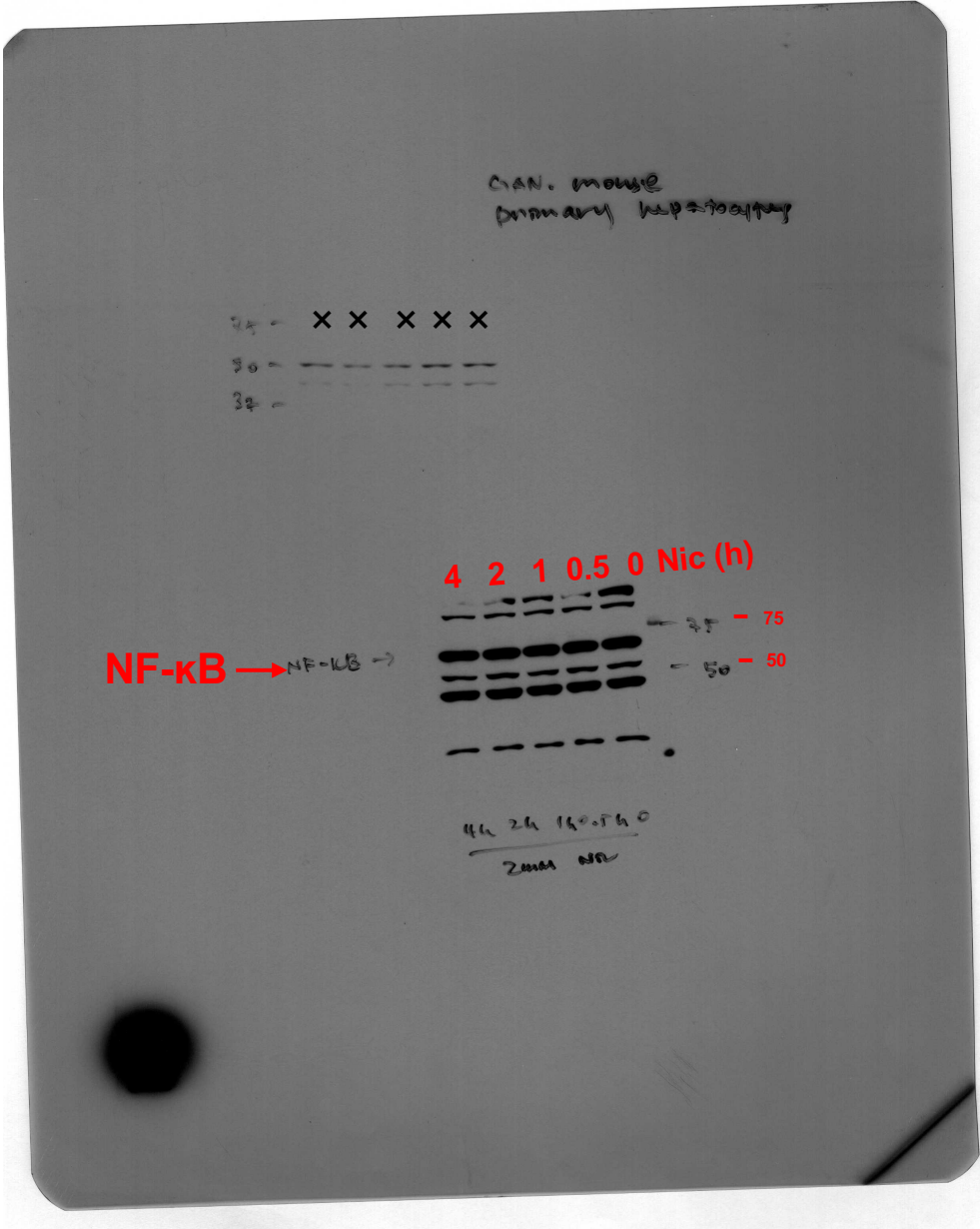

Fig. 1L

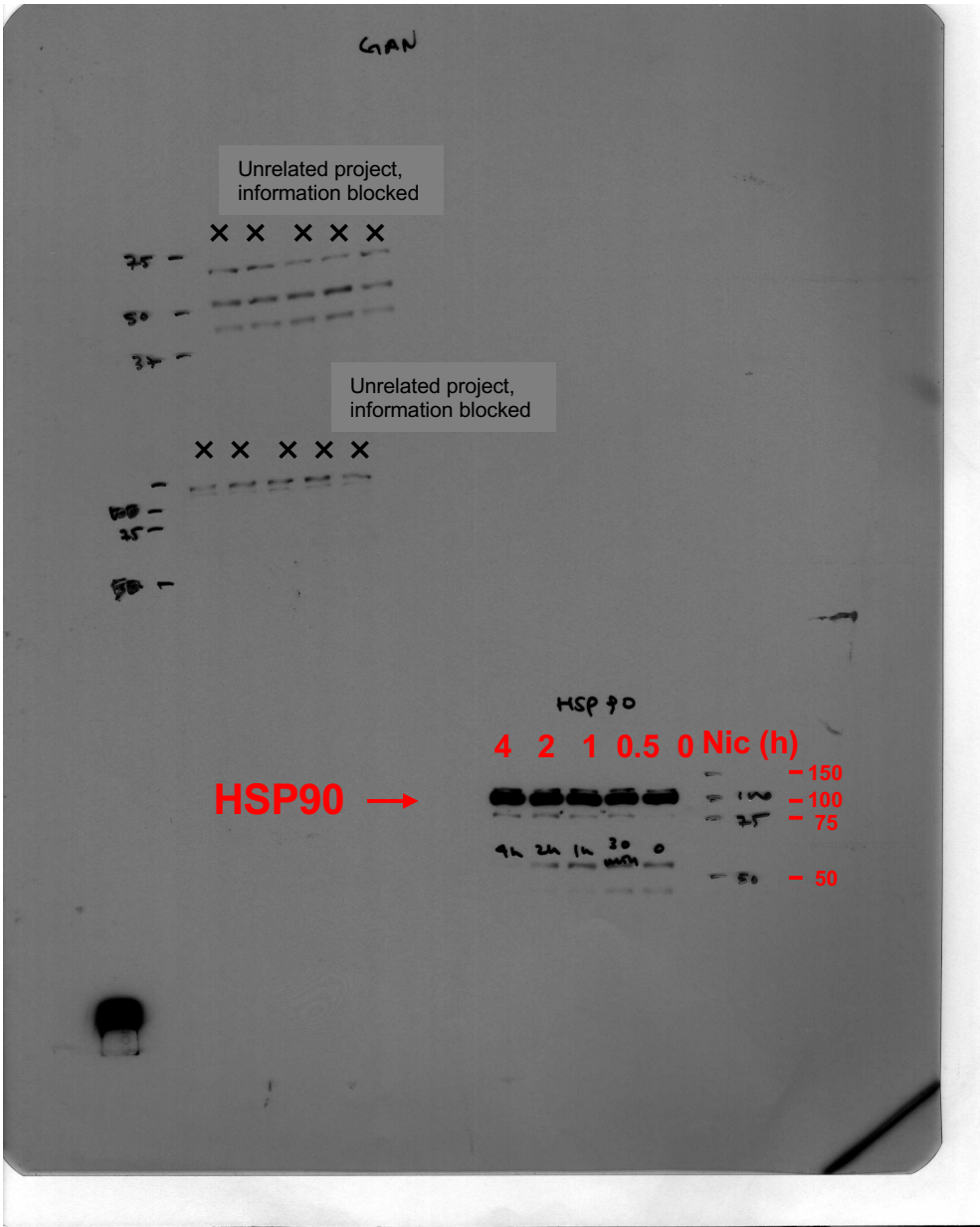

Fig. 1L

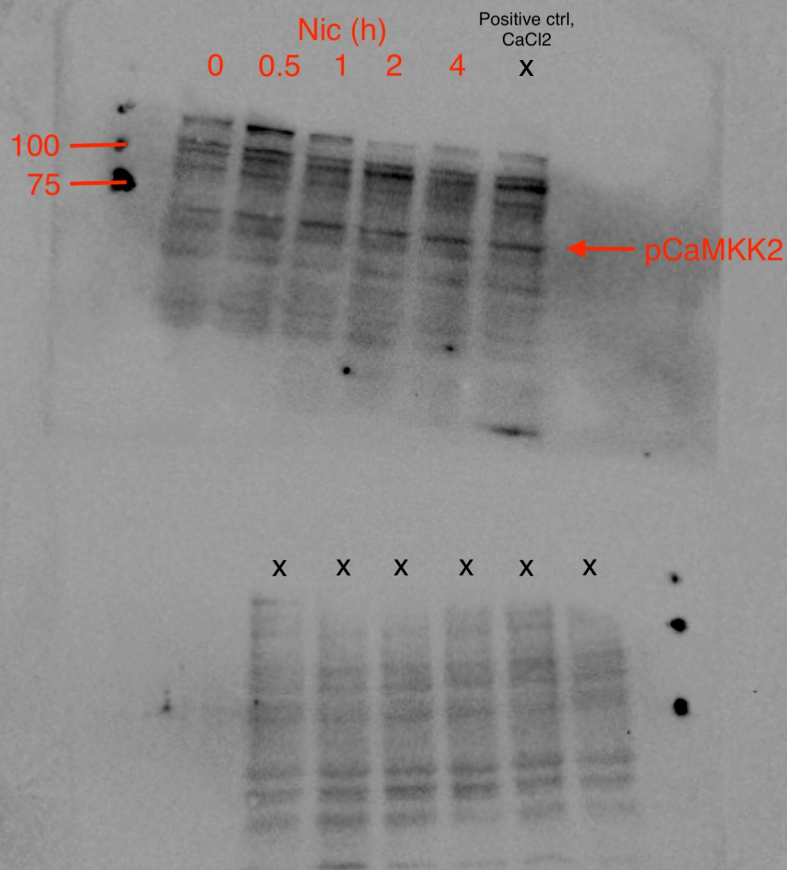

Supplement: S1 Raw Images — Source images underlying the graphs in Fig 1. (PDF) [file pbio.3002728.s028.pdf]

Fig. S1N

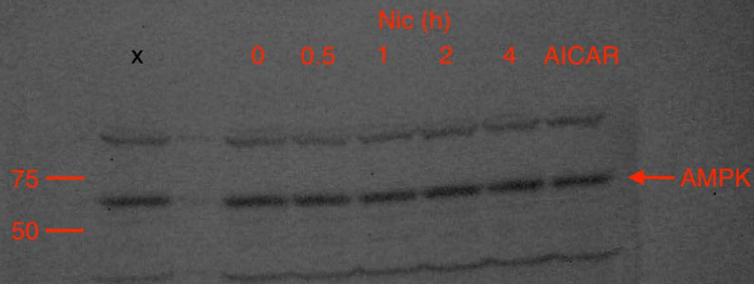

Fig. S1N

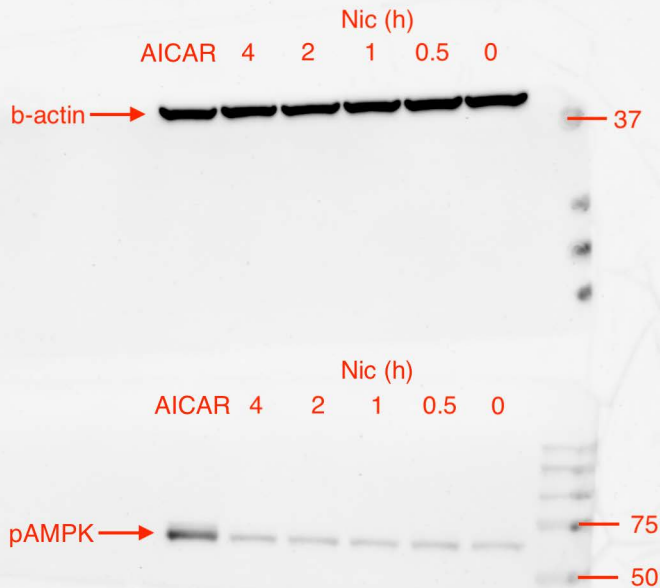

Supplement: S2 Raw Images — Source images underlying the graphs in S1 Fig. (PDF) [file pbio.3002728.s029.pdf]

Fig. S3C

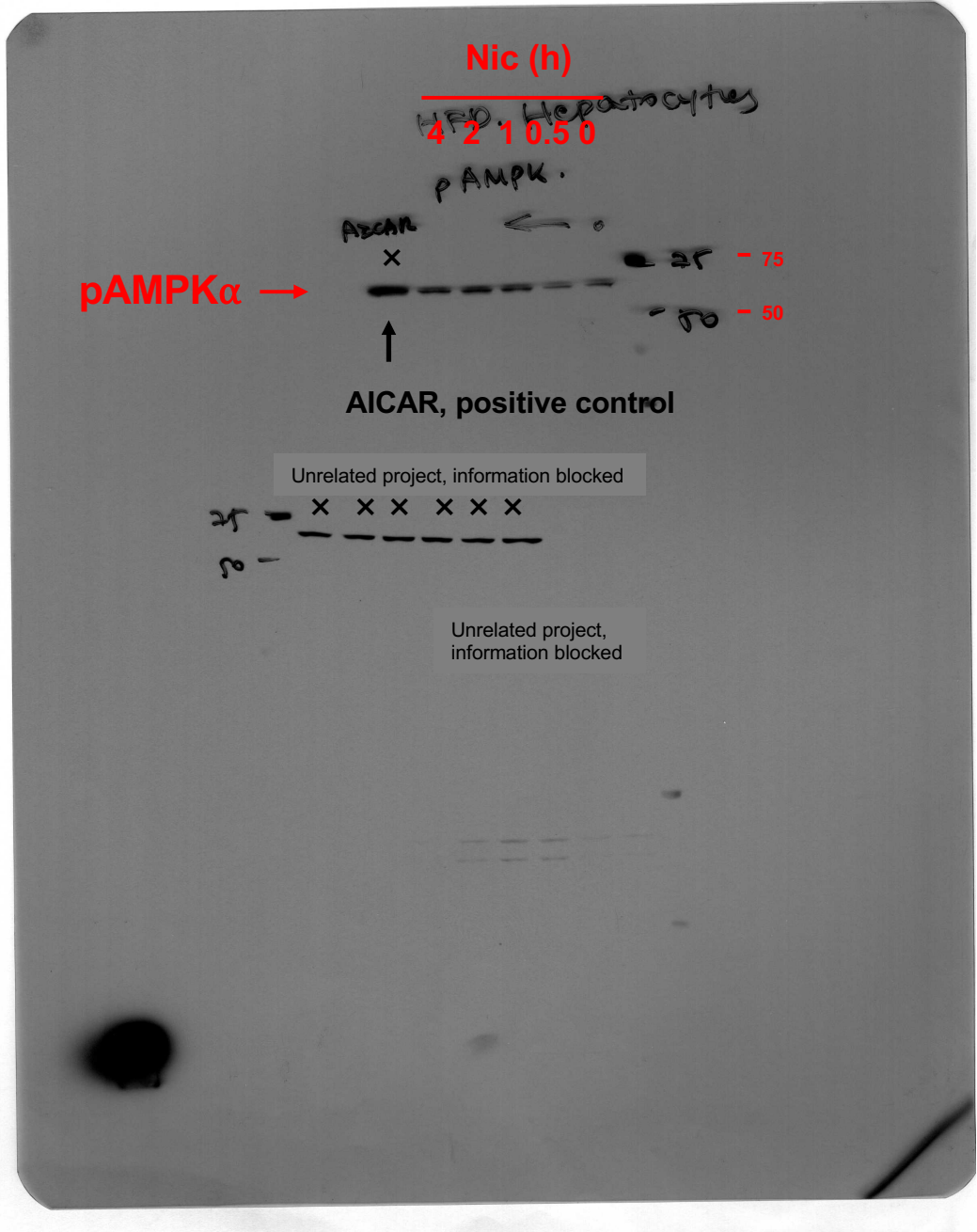

Fig. S3C

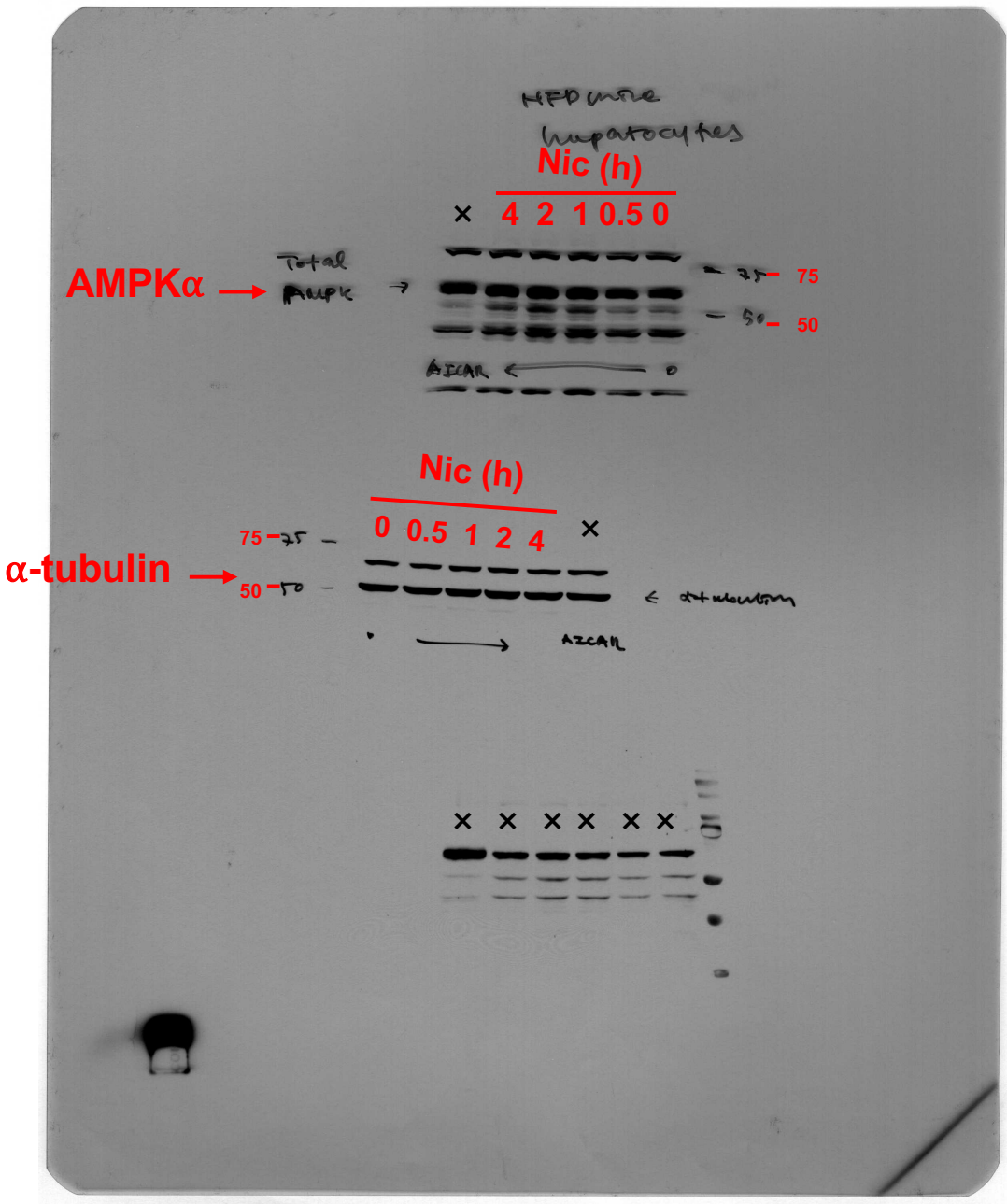

Supplement: S3 Raw Images — Source images underlying the graphs in S3 Fig. (PDF) [file pbio.3002728.s030.pdf]

Fig. S4H

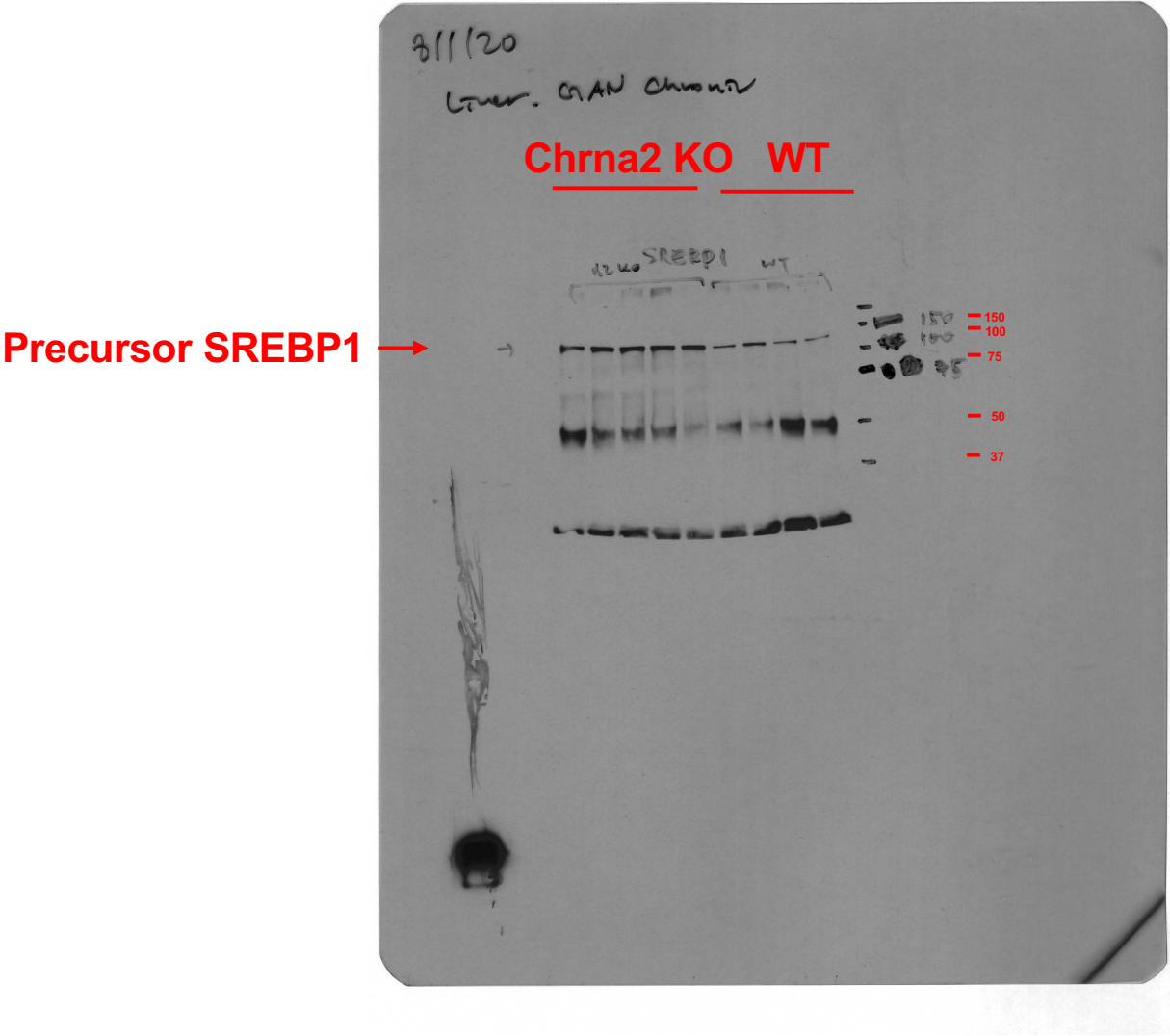

Fig. S4H

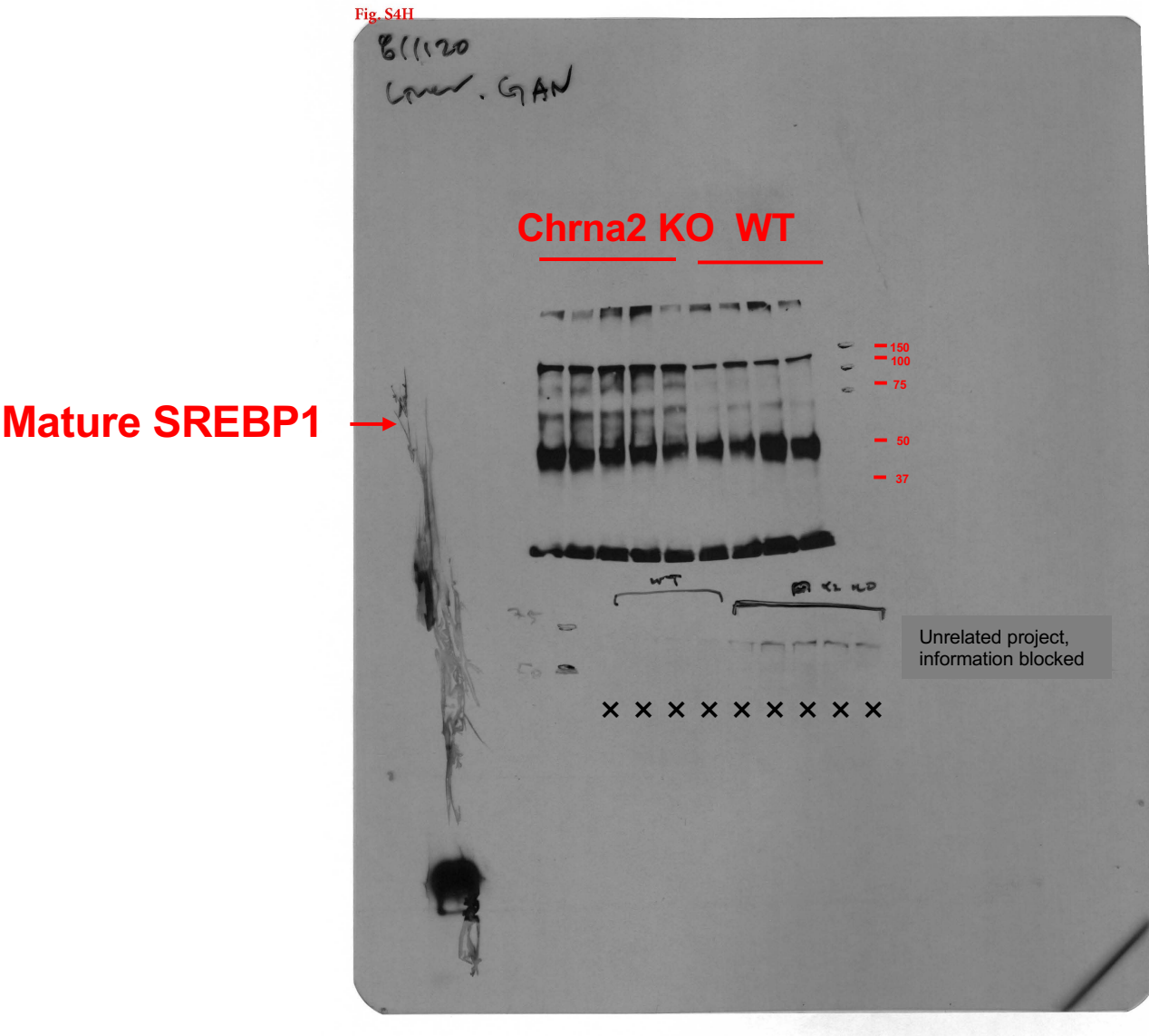

Fig. S4H

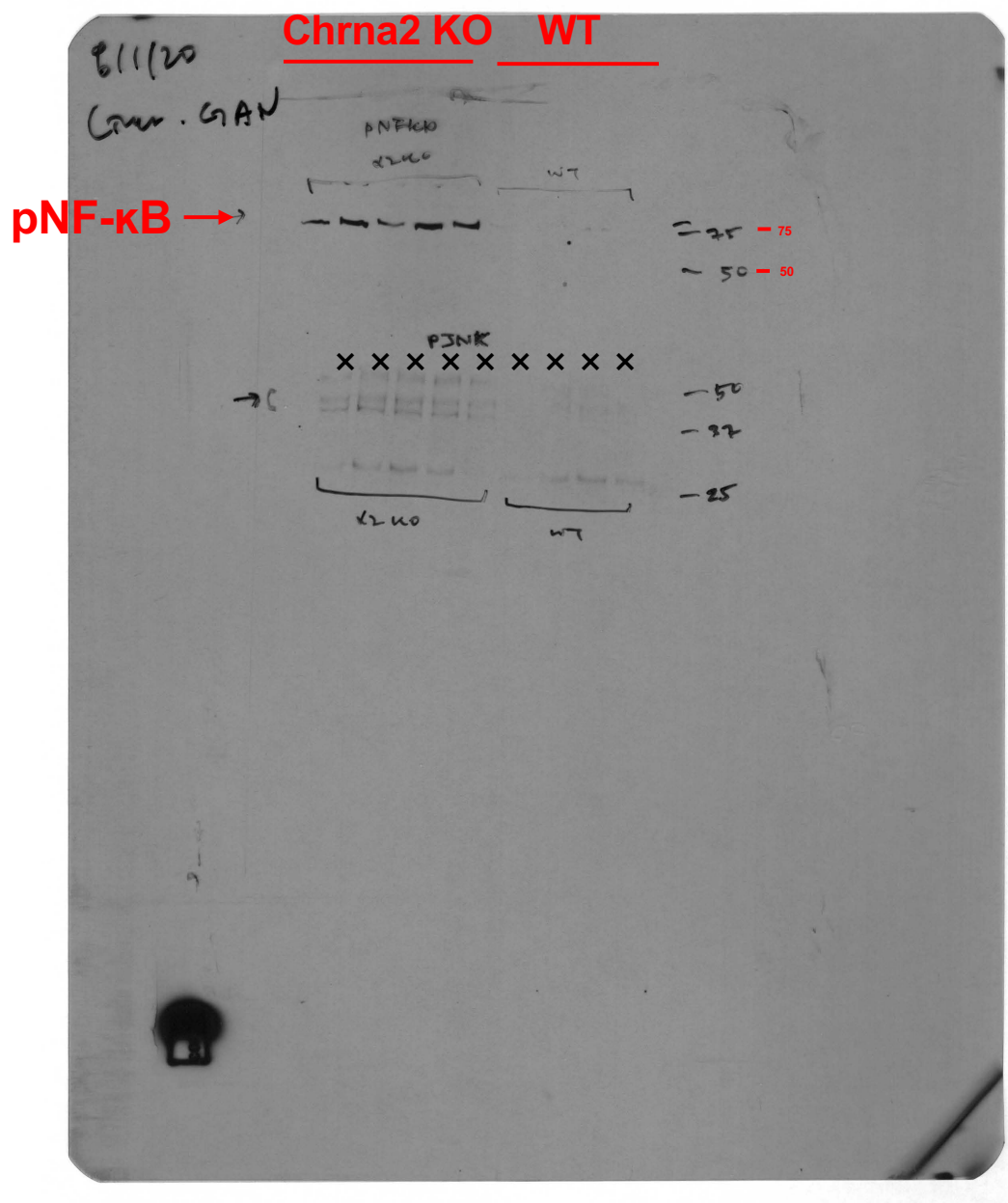

Fig. S4H

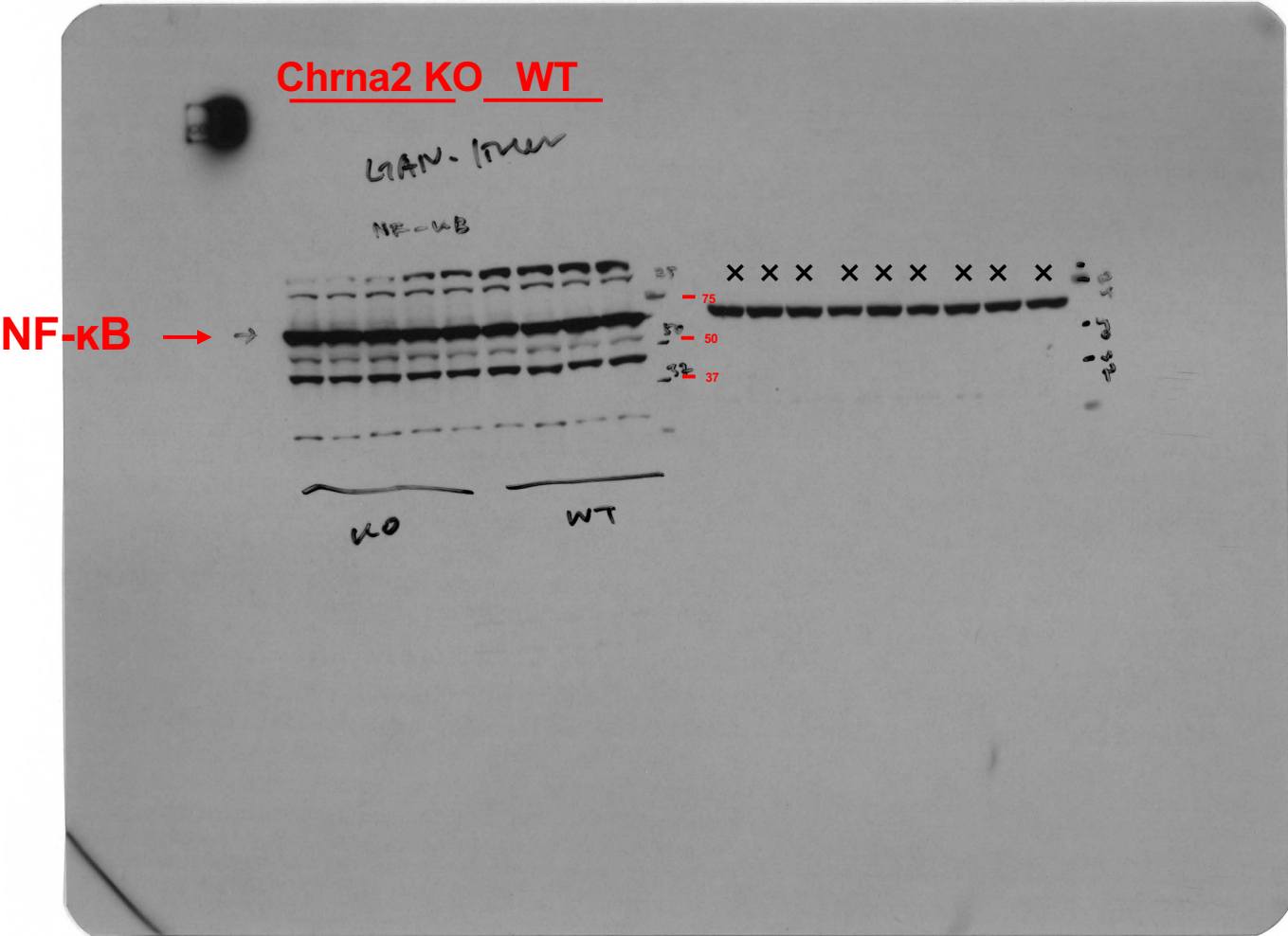

Fig. S4H

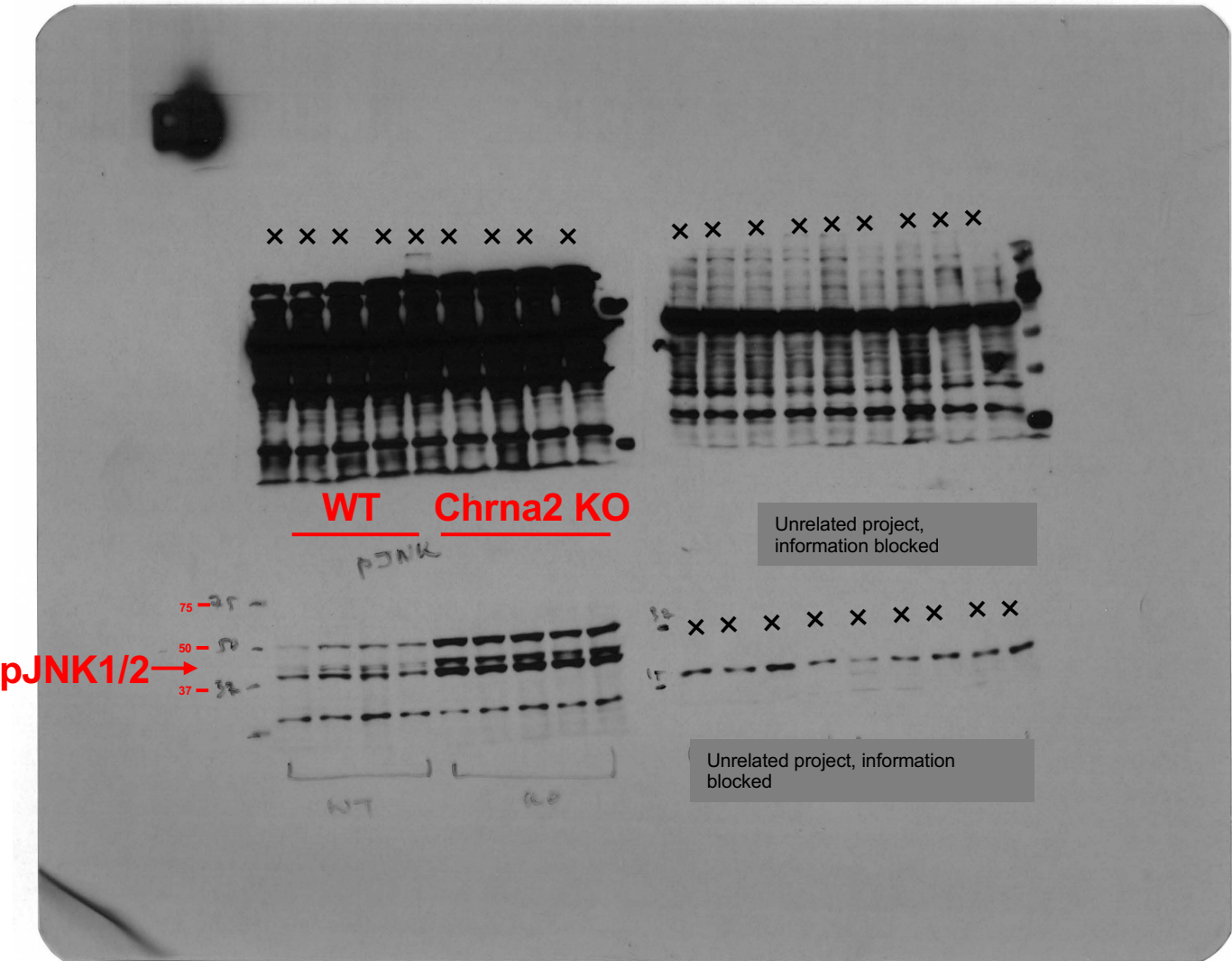

**Fig. S4H**

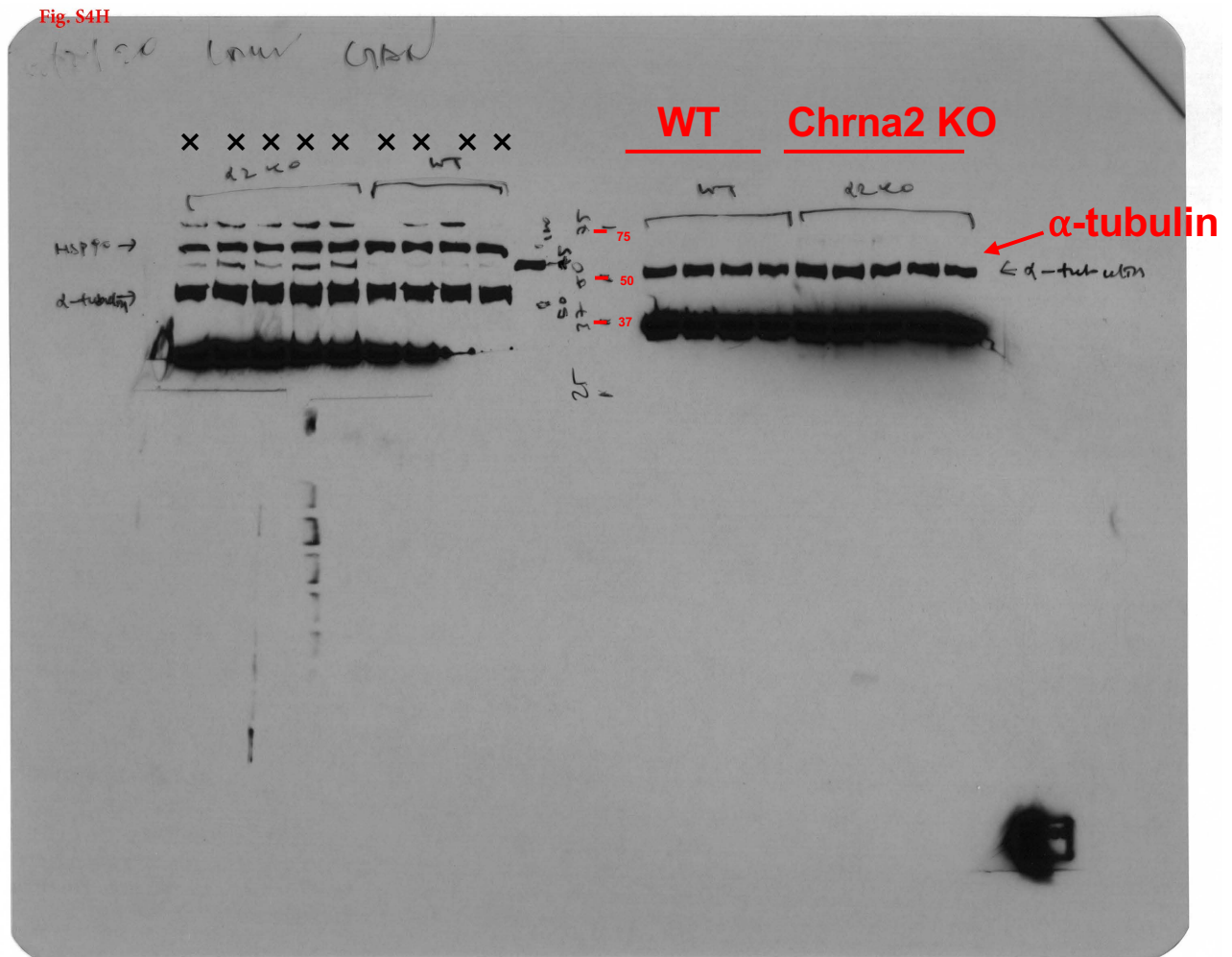

**Fig. S4H**

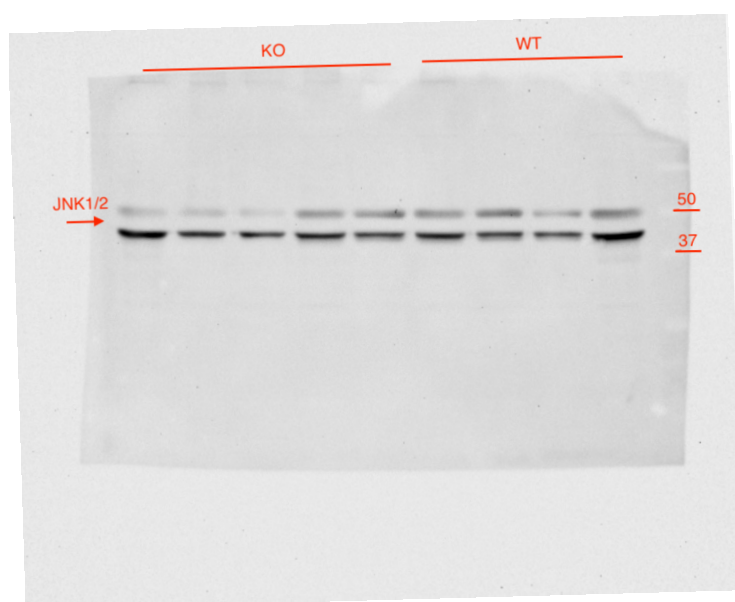

Supplement: S4 Raw Images — Source images underlying the graphs in S4 Fig. (PDF) [file pbio.3002728.s031.pdf]
