# Supplementary material for: Signaling through the nicotinic acetylcholine receptor in the liver protects against the development of metabolic dysfunction-associated steatohepatitis
Source: PLoS Biol. 2024 Jul 19;22(7):e3002728. doi: 10.1371/journal.pbio.3002728 (PMC11290650; doi:10.1371/journal.pbio.3002728)
Supplement: S1 Table — Relative frequencies of ChAT-eGFP+ hepatic non-parenchymal cell types. (DOCX) [file pbio.3002728.s011.docx]

**Table S1. Related to Fig 1.** Relative frequencies of ChAT-eGFP^+^ hepatic non-parenchymal cell types.

| Population gate in Fig S2. | Cell type* | Flow cytometry marker identification scheme | Frequency  ± SEM (%) |
| --- | --- | --- | --- |
| i | CD45^-^ | CD45^-^ | 1.4 ± 0.22 |
|  | CD45^+^ |  |  |
| ii | MDMs | CD45^+^ CD11b^hi^ F4/80^lo^ | 6.2 ± 1.14 |
| iii | KCs | CD45^+^ CD11b^lo^ F4/80^hi^ | 9.5 ± 1.29 |
| iv | γδ T cells | CD45^+^ CD11b^-^ F4/80^-^ CD3^+^ CD19^-^ TCRαβ^-^ TCRγδ^+^ | 3.3 ± 1.34 |
| v | CD8^+^ αβ T cells | CD45^+^ CD11b^-^ F4/80^-^ CD3^+^ CD19^-^ TCRαβ^+^ TCRγδ^-^ CD8^+^ CD4^-^ | 1.1 ± 0.22 |
| vi | CD4^+^ αβ T cells | CD45^+^ CD11b^-^ F4/80^-^ CD3^+^ CD19^-^ TCRαβ^+^ TCRγδ^-^ CD8^-^ CD4^+^ | 17.7 ± 1.91 |
| vii | CD4^-^ CD8^-^ (DN) αβ T cells | CD45^+^ CD11b^-^ F4/80^-^ CD3^+^ CD19^-^ TCRαβ^+^ TCRγδ^-^ CD8^-^ CD4^-^ | 2.1 ± 0.65 |
| viii | B220^+^ B cells | CD45^+^ CD11b^-^ F4/80^-^ CD3^-^ CD19^+^ B220^+^ | 19.7 ± 1.53 |
| ix | B220^-^ B cells | CD45^+^ CD11b^-^ F4/80^-^ CD3^-^ CD19^+^ B220^-^ | 34.0 ± 4.46 |
|  | Other CD45^+^ |  | 5.0 ± 2.66 |

*No significant presence of ChAT-eGFP^+^ neutrophils (CD45^+^ CD11b^+^ Ly6G^+^) was detected in the liver.
