# Supplementary material for: Signaling through the nicotinic acetylcholine receptor in the liver protects against the development of metabolic dysfunction-associated steatohepatitis
Source: PLoS Biol. 2024 Jul 19;22(7):e3002728. doi: 10.1371/journal.pbio.3002728 (PMC11290650; doi:10.1371/journal.pbio.3002728)
Supplement: S2 Table — (DOCX) [file pbio.3002728.s012.docx]

**Table S2.** qPCR primer sequences.

| **Gene** | **Forward** | **Reverse** |  |
| --- | --- | --- | --- |
| ***Human*** | | |  |
| Cholinergic receptor nicotinic alpha 2 subunit (*CHRNA2*) | AGGCTCGCATACCGAGACT | TCACCACGTCTGAAGTGTTGG |  |
| TATA-box binding protein (*TBP*) | CCACTCACAGACTCTCACAAC | CTGCGGTACAATCCCAGAACT |  |
| ***Mouse*** | | |  |
| Acetyl-CoA carboxylase alpha (*Acc1*) | GCCTCAGGAGGATTTGCTGT | AGGATCTACCCAGGCCACAT |  |
| Acyl-Coenzyme A dehydrogenase, long-chain (*Lcad*) | TCTTTTCCTCGGAGCATGACA | GACCTCTCTACTCACTTCTCCAG |  |
| Acyl-Coenzyme A dehydrogenase, medium chain (*Mcad*) | AGGGTTTAGTTTTGAGTTGACGG | CCCCGCTTTTGTCATATTCCG |  |
| Actin alpha 2, smooth muscle (*Acta2*) | ACTGGGACGACATGGAAAAG | GTTCAGTGGTGCCTCTGTCA |  |
| Activating transcription factor 4 (*Atf4)* | ATGGCCGGCTATGGATGAT | CGAAGTCAAACTCTTTCAGATCCATT |  |
| ATP synthase, H+ transporting mitochondrial F1 complex, beta subunit (*Atp5b*) | GGTTCATCCTGCCAGAGACTA | AATCCCTCATCGAACTGGACG |  |
| C-C motif chemokine ligand 2 (*Ccl2*) | TGACCCCAAGAAGGAATGGG | ACCTTAGGGCAGATGCAGTT |  |
| Choline acetyltransferase (*Chat*) | TGCCAGTCAACTCTAGCCCT | CTGCAAACCTTAGCTGGTCATT |  |
| Cholinergic receptor, nicotinic, alpha polypeptide 1 (muscle) (*Chrna1*) | ACCAATGTACGTCTGAAACAGC | TTTTCCGAGGGGATGTGAATTTT |  |
| Cholinergic receptor, nicotinic, alpha polypeptide 10 (*Chrna10*) | ATGGATGAACGGAACCAAGTG | GTCCCAATGTAGGTAGGCGT |  |
| Cholinergic receptor, nicotinic, alpha polypeptide 2 (neuronal) (*Chrna2*) | GCCACCGGAACCTATAACAGC | CCGGCGGATAACGAAGTAGTA |  |
| Cholinergic receptor, nicotinic, alpha polypeptide 2 (neuronal) (*Chrna2*) (for *Chrna2*^fl/fl^;*AlbCre*) | CCATCCTGCTTTCCAGTTCT | GATAGTCCAAAGCGGACGAT |  |
| Cholinergic receptor, nicotinic, alpha polypeptide 3 (*Chrna3*) | TGGAAACCCTCTGACTACCAA | GGCGTTGTTGTAAAGCACGA |  |
| Cholinergic receptor, nicotinic, alpha polypeptide 4 (*Chrna4*) | TCGTCTAGAGCCCGTTCTGT | GGCCGAGACCACTTGTTGTA |  |
| Cholinergic receptor, nicotinic, alpha polypeptide 5 (*Chrna5*) | GTTGCCTGAGCTATCCTCTGC | GACGAACCCACTTTTCATAGTCT |  |
| Cholinergic receptor, nicotinic, alpha polypeptide 6 (*Chrna6*) | TTGCTCACTACAACCGCTTCA | GCGTGATTGCCAATTCAAAATGC |  |
| Cholinergic receptor, nicotinic, alpha polypeptide 7 (*Chrna7*) | AGTTTTAACCACCAACATTTGGC | TTTTCACTCCGGGGTACTCAG |  |
| Cholinergic receptor, nicotinic, alpha polypeptide 9 (*Chrna9*) | CAGGTCACGCTCTCCCAGATA | GTACGCATCGTGCCAAGTTT |  |
| Cholinergic receptor, nicotinic, beta polypeptide 1 (muscle) (*Chrnb1*) | ACTACAGGTTAAGCTGGGACC | GTCCAGGGCAACGTCGAAAT |  |
| Cholinergic receptor, nicotinic, beta polypeptide 2 (neuronal) (*Chrnb2*) | AGGGGTTTTGGGTACTGACAC | GGATCAGCTTGTTATAGCGGGAA |  |
| Cholinergic receptor, nicotinic, beta polypeptide 3 (*Chrnb3*) | TGCTGACGGACGTTTTGAGG | GAGGAGTCCAACTGACGGT |  |
| Cholinergic receptor, nicotinic, beta polypeptide 4 (*Chrnb4*) | GCTATGTGGACGTGACCTATG | CGACGTGATGAGCACACAAG |  |
| Collagen type I alpha 1 chain (*Col1a1*) | TGACTGGAAGAGCGGAGAGT | GTTCGGGCTGATGTACCAGT |  |
| Cytochrome c oxidase subunit I (*Cox1*) | CTACTATTCGGAGCCTGAGC | GCATGGGCAGTTACGATAAC |  |
| Cytochrome c oxidase subunit IV isoform 1 (*Cox4*) | ACCAAGCGAATGCTGGACAT | GGCGGAGAAGCCCTGAA |  |
| Cytochrome c oxidase subunit VIIIb (*Cox8b*) | GCGAAGTTCACAGTGGTTCC | GAACCATGAAGCCAACGACT |  |
| C-X-C motif chemokine ligand 10 (*Cxcl10*) | CCAAGTGCTGCCGTCATTTTC | GGCTCGCAGGGATGATTTCAA |  |
| C-X-C motif chemokine ligand 2 (*Cxcl2*) | CGGTCAAAAAGTTTGCCTTG | TCCAGGTCAGTTAGCCTTGC |  |
| DNA damage inducible transcript 3 (*Chop*) | CTGCCTTTCACCTTGGAGAC | CGTTTCCTGGGGATGAGATA |  |
| DnaJ heat shock protein family (Hsp40) member B9 (*Erdj4*) | TCAGAGAGATTGCAGAAGCG | GACTCCCATTGCCTCTTTGT |  |
| Fatty acid synthase (*Fasn*) | GGAGGTGGTGATAGCCGGTAT | TGGGTAATCCATAGAGCCCAG |  |
| Glycerol-3-phosphate acyltransferase, mitochondrial (*Gpat1*) | CAACACCATCCCCGACATC | GTGACCTTCGATTATGCGATCA |  |
| Lipase, hormone-sensitive (*Hsl*) | TGAGATGCCACTCACCTCTG | GCCTAGTGCCTTCTGGTCTG |  |
| Interleukin 1 beta (*Il1b*) | GCAACTGTTCCTGAACTCAACT | ATCTTTTGGGGTCCGTCAACT |  |
| Keratin 18 (*Ck18*) | CAGCCAGCGTCTATGCAGG | CCTTCTCGGTCTGGATTCCAC |  |
| Matrix metallopeptidase 13 (*Mmp13*) | CTTCTGGCACACGCTTTTC | ATGCTTAGGGTTGGGGTCTT |  |
| Matrix metallopeptidase 2 (*Mmp2*) | GGACAAGTGGTCCGCGTAAA | CCGACCGTTGAACAGGAAGG |  |
| Monoglyceride lipase (*Mgll*) | TGATTTCACCTCTGGTCCTTG | GTCAACCTCCGACTTGTTCC |  |
| NADH dehydrogenase (ubiquinone) flavoprotein 1 (*Ndufv1*) | TTTCTCGGCGGGTTGGTTC | GGTTGGTAAAGATCCGGTCTTC |  |
| NADH dehydrogenase subunit 1 (*Nd1*) | TCCGAGCATCTTATCCACGC | GTATGGTGGTACTCCCGCTG |  |
| Nitric oxide synthase 2 (*iNOS*) | GTTCTCAGCCCAACAATACAAGA | GTGGACGGGTCGATGTCAC |  |
| Patatin-like phospholipase domain containing 2 (*Atgl*) | AACACCAGCATCCAGTTCAA | GGTTCAGTAGGCCATTCCTC |  |
| Peroxisome proliferator-activated receptor gamma, coactivator 1 alpha (*Ppargc1a*) | AGCCGTGACCACTGACAACGAG | GCTGCATGGTTCTGAGTGCTAAG |  |
| Protein tyrosine phosphatase receptor type C (*Cd45*) | GAACATGCTGCCAATGGTTCT | TGTCCCACATGACTCCTTTCC |  |
| Red fluorescent protein (RFP) | | GCGTTACATAACTTACGGTAAATGGCCC | GGGCGTACTTCGCATATGATACACTTGATG |
| Stearoyl-Coenzyme A desaturase 1 (*Scd1)* | TTCTTGCGATACACTCTGGTGC | CGGGATTGAATGTTCTTGTCGT |  |
| Sterol regulatory element binding transcription factor 1 (*Srebp1c*) | GGAGCCATGGATTGCACATT | GGCCCGGGAAGTCACTGT |  |
| Succinate dehydrogenase complex, subunit A, flavoprotein (Fp) (*Sdha*) | GGAACACTCCAAAAACAGACCT | CCACCACTGGGTATTGAGTAGAA |  |
| Succinate dehydrogenase complex, subunit B, iron sulfur (Ip) (*Sdhb*) | AATTTGCCATTTACCGATGGGA | AGCATCCAACACCATAGGTCC |  |
| Transcription factor A, mitochondrial (Tfam) | ATTCCGAAGTGTTTTTCCAGCA | TCTGAAAGTTTTGCATCTGGGT |  |
| TATA-box binding protein (*Tbp*) | CCCCTTGTACCCTTCACCAAT | GAAGCTGCGGTACAATTCCAG |  |
| TIMP metallopeptidase inhibitor 1 (*Timp1*) | GGGTTCCCCAGAAATCAACGA | ACCGGATATCTGCGGCATTT |  |
| Tumor necrosis factor *(Tnf)* | CCCTCACACTCAGATCATCTTCT | GCTACGACGTGGGCTACAG |  |
| Ubiquinol cytochrome c reductase core protein 2 (*Uqcrc2*) | AAAGTTGCCCCGAAGGTTAAA | GAGCATAGTTTTCCAGAGAAGCA |  |
| Uncoupling protein 1 (*Ucp1*) | CTTTGCCTCACTCAGGATTGG | ACTGCCACACCTCCAGTCATT |  |
| Uncoupling protein 3 (*Ucp3*) | GGATTTGTGCCCTCCTTTCTG | AGATTCCCGCAGTACCTGGACT |  |
| Vascular endothelial growth factor A (*Vegfa*) | GCACATAGAGAGAATGAGCTTCC | CTCCGCTCTGAACAAGGCT |  |
| X-box binding protein 1*,* spliced form *(Xbp1s*) | GAGTCCGCAGCAGGTG | GTGTCAGAGTCCATGGGA |  |
