# Supplementary material for: Signaling through the nicotinic acetylcholine receptor in the liver protects against the development of metabolic dysfunction-associated steatohepatitis
Source: PLoS Biol. 2024 Jul 19;22(7):e3002728. doi: 10.1371/journal.pbio.3002728 (PMC11290650; doi:10.1371/journal.pbio.3002728)
Supplement: S3 Table — (DOCX) [file pbio.3002728.s013.docx]

**Table S3.** Antibodies used in the study.

| **Antibodies** | **Source** | **Cat#** |
| --- | --- | --- |
| Anti-rabbit phospho-AMPKα^T172^ | Cell Signaling | 2531S |
| Anti-rabbit AMPKα | Cell Signaling | 2532 |
| Anti-rabbit phosphor-CaMKK2^S511^ | Cell Signaling | 12818S |
| Anti-rabbit phospho-JNK1/2^T183/Y185^ | Cell Signaling | 9251 |
| Anti-rabbit JNK1/2 | Cell Signaling | 9252 |
| Anti-rabbit phospho-NF-κB p65^S536^ | Cell Signaling | 3033 |
| Anti-rabbit NF-κB p65 | Cell Signaling | 8242 |
| Anti-rabbit TdTomato | Cell Signaling | 20163 |
| Anti-rabbit SREBP1 | Santa Cruz | sc-13551 |
| Anti-rabbit α-Tubulin | Cell Signaling | 2144 |
| Anti-rabbit HSP90 | Cell Signaling | 4874 |
| Anti-rabbit β-Actin | Cell Signaling | 8457 |
| APC/Cy7 anti-mouse CD3 clone 17A2 | Biolegend | 100221 |
| BV510 anti-mouse Ly6G clone RB6-8C5 | Biolegend | 108457 |
| APC/Cy7 anti-mouse Ly6G clone 1A8 | Biolegend | 127623 |
| BV605 anti-mouse CD11b clone M1/70 | Biolegend | 101237 |
| PE/Cy7 anti-mouse CD19 clone 6D5 | Biolegend | 115520 |
| BV650 anti-mouse CD45 clone 30-F11 | Biolegend | 103151 |
| APC-R700 anti-mouse F4/80 clone T45-2342 | BD Horizon | 565787 |
| BV605 anti-mouse CD4 clone GK1.5 | Biolegend | 100451 |
| BV421 anti-mouse CD8a clone 53-6.7 | Biolegend | 100737 |
| AF700 anti-mouse B220 clone RA3-6B2 | Biolegend | 103232 |
| BV510 anti-mouse TCRβ clone H57-597 | Biolegend | 109233 |
| PE anti-mouse TCRγ/δ clone UC7-13D5 | Biolegend | 107507 |
| TruStain FcX PLUS (anti-mouse CD16/32) clone S17011E | Biolegend | 156604 |
